# Synthesis and Fungicidal Activity of Hydrated Geranylated Phenols against *Botrytis cinerea*

**DOI:** 10.3390/molecules26226815

**Published:** 2021-11-11

**Authors:** Mauricio Soto, Ana Estevez-Braun, Ángel Amesty, Julia Kluepfel, Susana Restrepo, Katy Diaz, Luis Espinoza, Andrés F. Olea, Lautaro Taborga

**Affiliations:** 1 Departamento de Química, Universidad Técnica Federico Santa María, Av. España No. 1680, Valparaíso 2340000, Chile; mauricio.sotoc.13@sansano.usm.cl (M.S.); susana.restrepo@usm.cl (S.R.); katy.diaz@usm.cl (K.D.); luis.espinozac@usm.cl (L.E.); 2 Instituto Universitario de Bio-Orgánica Antonio González (CIBICAN), Departamento de Química Orgánica, Universidad de La Laguna, Av. Astrofísico Fco, Sánchez 2, 38206 La Laguna, Spain; aestebra@ull.edu.es (A.E.-B.); aarnesty@ull.es (Á.A.); 3 Department of Chemistry, Technical University of Munich, Lichtenberg Str. 4, 85748 Garching, Germany; julia.kluepfel@tum.de; 4 Grupo de Química y Bioquímica Aplicada en Biotecnología, Instituto de Ciencias Químicas Aplicadas, Facultad de Ingeniería, Universidad Autónoma de Chile, El Llano Subercaseaux 2801, Santiago 8900000, Chile

**Keywords:** *Botrytis cinerea*, hydrated geranylated phenols, docking, synthesis, fungicidal

## Abstract

*Botrytis cinerea* is a ubiquitous fungus that affects hundreds of plants, resulting in economic losses to the horticulture and fruit industry. The search for new antifungal agents is a matter of current interest. Thus, in this work a series of geranylated phenols in which the side alkyl chain has been hydrated have been synthesized, and their activity against *B. cinerea* has been evaluated. The coupling of phenol and geraniol has been accomplished under microwave irradiation obtaining the highest reaction yields in the shortest reaction times. Hydration of the side chain was carried out in dioxane with *p*-toluenesulfonic acid polymer-bound as the catalyst. All synthesized compounds were tested against *B. cinerea* using the growth inhibition assay and EC_50_ values were determined. The results show that activity depends on the number and nature of functional groups in the phenol ring and hydration degree of the geranyl chain. The most active compound is 1,4-dihydroquinone with one hydroxyl group attached at the end of the alkyl chain. Results from a molecular docking study suggest that hydroxyl groups in the phenol ring and alkyl chain are important in the binding of compounds to the active site, and that the experimental antifungal activity correlates with the number of H-bond that can be formed in the binding site.

## 1. Introduction

Geranylated phenols and their hydrated derivatives are an important class of secondary metabolites found mainly in marine organisms. They have been isolated from brown algaes (fucales), sponges [1,2], coral [3,4] and Ascidian (*Aplidium*) [5,6,7,8].

**Figure 1:**
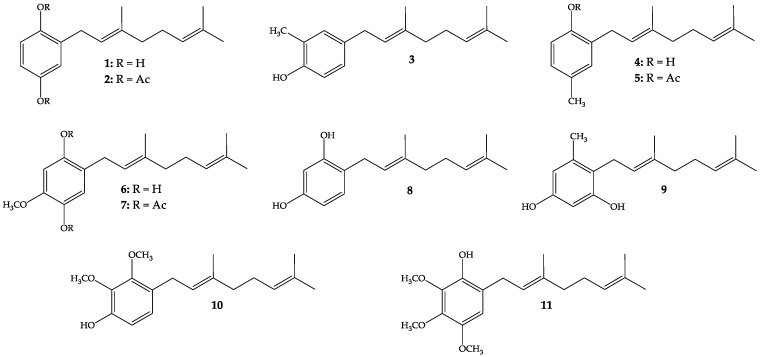
Geranylated phenols with antifungal activity.

De Rosa et al. [1] have shown that biological activity of 2-prenyl-1,4-hydroquinones depends on the size of the prenyl chain, and the optimal length is in the range of five to fifteen carbon atoms. In previous work, we reported the synthesis of a series of geranylated phenols with different chemical substituents on the aromatic ring (see Figure 1). These compounds exhibited interesting biological activities, such as in vitro cytotoxicity on cancer cell lines [9,10], mycelial growth inhibition of *Botrytis cinerea* [11,12,13,14] and antifungal activity against *Phytophthora cinnamomi* [15]. The results indicate that the activity is enhanced mainly by the presence of −OH, –OCH_3_ and –OAc functions in the aromatic ring [11,12,13].

**Figure 2:**
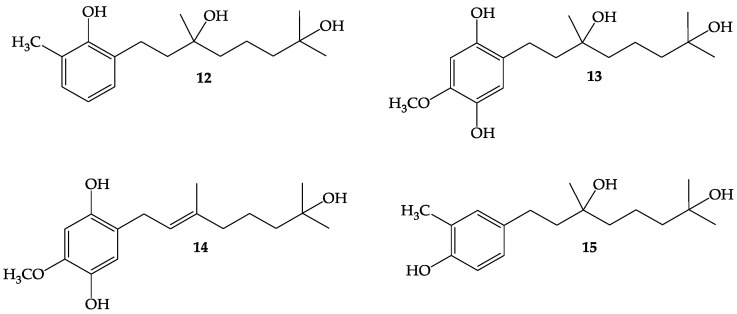
Geranylated phenols with hydrated side chain.

On the other hand, the effect of chemical modification of the side chain on antifungal activity has received much less attention, i.e., it has been shown that the hydrated derivative of 2-allylphenol exhibits antifungal activity against *P. aphanidermatum*, *V. mali*, *B. cinerea* and *R. cereals* [16], whereas some hydrated derivatives of geranylated phenols (Figure 2) inhibit the mycelial growth of *B. cinerea* [14].

Thus, the aim of this work is to carry out a systematic study of the effect of ring substitution and hydration of side chain on the activity of geranylated phenols against *B. cinerea*. Therefore, a series of geranylated phenols with and without hydroxyl groups in the side chain were synthesized, and their mycelial growth inhibition activity on *B. cinerea* was determined. The obtained data were used to establish a structure–activity relationship, whereas a molecular docking study was made to visualize the main interactions of these molecules into the active site.

## 2. Results

To establish a structure–activity relationship, between mycelial-growth inhibition of *B. cinerea* and chemical substitution on phenol ring and hydration in the side alkyl chain, four series of geranylated phenols have been synthesized. Antifungal activities against *B. cinerea* of all synthesized compounds were evaluated.

### 2.1. Synthesis

**Figure 3:**
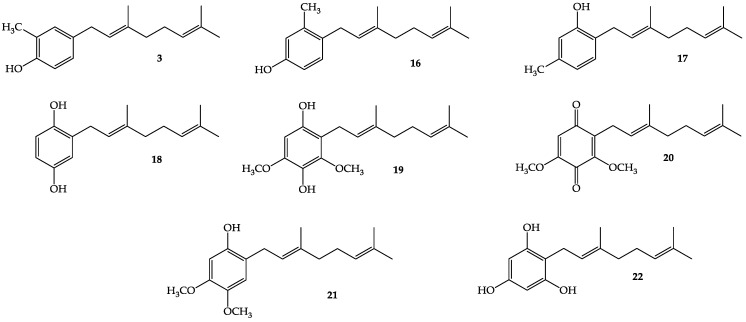
Series of geranylated phenols synthesized by microwave irradiation using acetonitrile as solvent. Compounds **19** and **20** are new.

The synthesis of geranylated phenols is well described and generally it proceeds by direct coupling of geraniol with the proper phenol in the presence of BF_3_·OEt_2_ as a catalyst and dioxane as solvent [1,9,14,17,18,19]. Alternatively, BF_3_·OEt_2_/AgNO_3_ has been used as catalyst and acetonitrile as solvent [11,12]. The time reaction varies between 24 and 72 h and the yields are in the range of 3 to 12%. In this work, this coupling reaction was carried out under microwave irradiation to obtain a series of geranylated phenols with different substituents on the phenolic ring (see Figure 3).

**Scheme 1:**
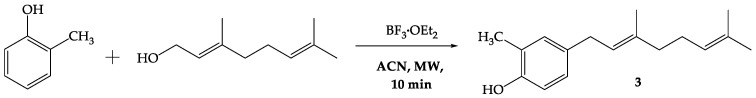
Synthesis of geranylated phenol 3 under microwave irradiation.

Compounds **3**, **18**, **16**, **17**, **21** and **22** have been described previously, whereas derivatives **19** and **20** are new compounds. Interestingly, coupling reactions under microwave irradiation proceed with higher yields (21–32%) and reduced reaction time (10 min). For example, Scheme 1 shows the coupling reaction between o-cresol and geraniol giving compound **3**. Under microwave irradiation, three is obtained with 22% yield after 10 min, while in absence of irradiation the reaction yield is 3% after 48 h of reaction [14].

**Figure 4:**
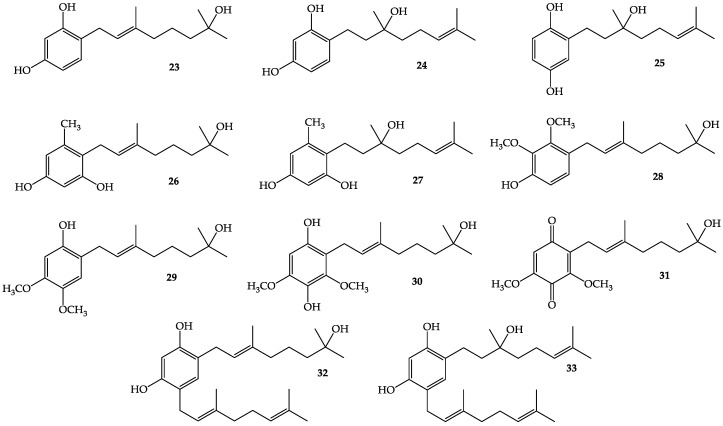
Synthesized geranylated phenols with monohydrated side chain.

**Figure 5:**
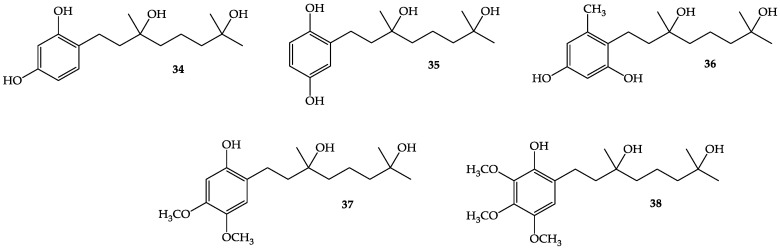
Geranylated phenols with di-hydrated side chain.

On the other hand, there are just a few reports of hydration reaction of the side chain in geranylated phenols. Hydrated geranylated orcinol derivatives were obtained by a coupling reaction of geraniol and orcinol in the presence of oxalic acid (1%) at 80 °C [20]. No reaction yields were reported. Compounds **13** and **14** were synthesized by electrophilic addition of geraniol in dioxane and using BF_3_ as catalyst in the presence of water [14]. Herein, geranylated phenols were hydrated in dioxane containing water and using *p*-toluenesulfonic acid bound to a polymer matrix as catalyst. In this way, two series of new mono- and di-hydrated geranylated phenols (Figure 4 and Figure 5, respectively) were obtained.

**Scheme 2:**
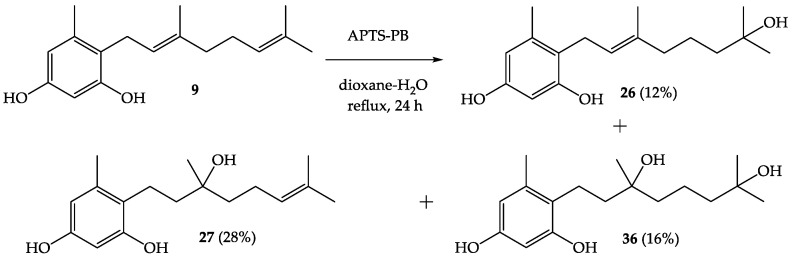
Synthesis of geranylated orcinol derivatives with mono-hydrated (**26**, **27**) and di-hydrated side chain (**36**).

The reaction conditions and products of hydration of 4-geranylated orcinol are shown in Scheme 2.

The product of this reaction is a mixture of hydrated compounds carrying one (**26** and **27**) or two hydroxyl groups (**36**). As can be seen, the product reaction is a mixture of hydrated compounds carrying one or two hydroxyl groups.

Structural Determination of Geranylated Orcinol Derivatives **26**, **27** and **36**

**Figure 6:**
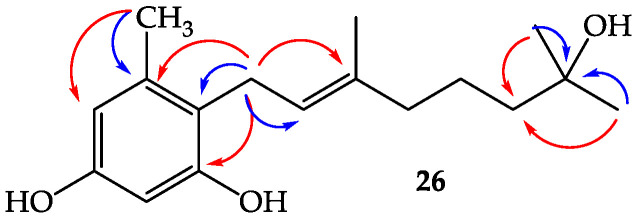
Main heteronuclear correlations 2D HMBC at ^2^JCH (blue) and ^3^JCH (red) observed for compound **26**.

The chemical structure of hydrated geranylated phenols was established mainly by ^1^H, ^13^C (Figures S5, S6 and S15, Supplementary Material) and 2D NMR spectroscopy. The disappearance of double bonds in the side alkyl chain was verified in the ^1^H-NMR spectrum by signals appearing at *δ* = 3.28 (2H, *d*, *J* = 6,8 Hz); *δ* = 5.13 (1H, m); *δ* = 1.40 (1H, m) assigned to H-1′, H-2′ and H-6′, respectively. These assignments were made by comparison with NMR spectra previously published for the respective geranylated phenol, and by 2D HMBC spectra. Figure 6 shows heteronuclear correlations at ^2^JCH (blue) and ^3^JCH (red) observed for compound **26**, i.e., coupling at ^3^JCH (red) between H-1′ and C-3′ (*δ*_C_ = 137.9 ppm), C-5 (*δ*_C_ = 138.5 ppm) and C-3 (*δ*_C_ = 155.3 ppm) and coupling at ^2^JCH (blue) between H-1′ and C-4 (*δ*_C_ = 117.9 ppm) and C-2′ (*δ*_C_ = 122.4 ppm).

**Figure 7:**
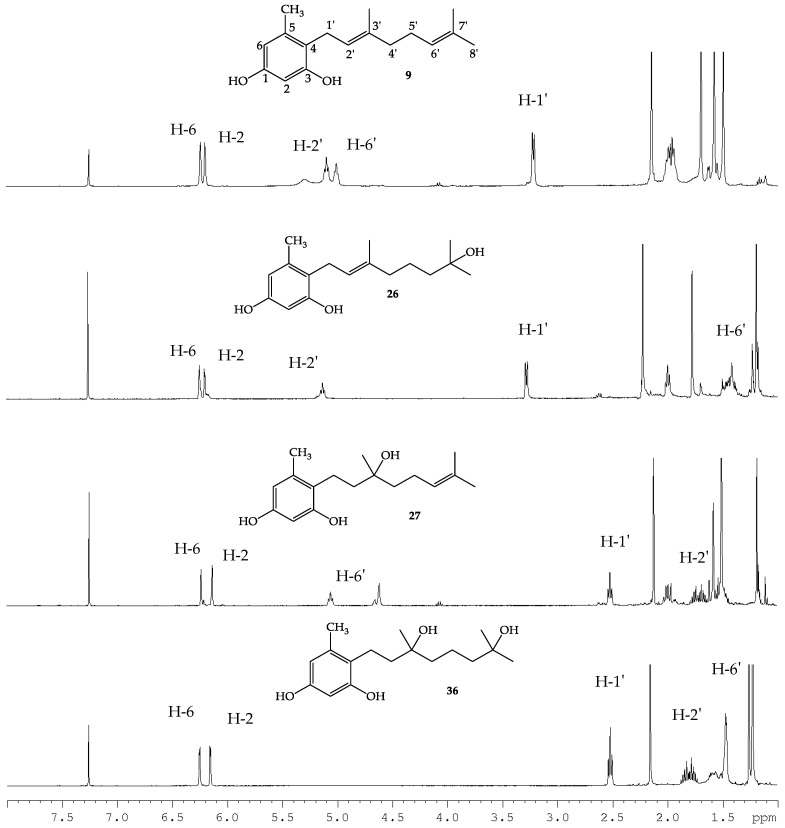
Comparison between ^1^H-NMR spectra of geranylated orcinol derivatives **9**, **26**, **27** and **36**.

Figure 7 shows ^1^H-NMR spectra of geranylated orcinol and mono-hydrated and dehydrated derivatives obtained as products of reactions described in Scheme 1 and Scheme 2. Signals due to H atoms involved in the hydration reaction are clearly depicted to demonstrate the attaching of hydroxyl groups to one or both double bonds.

The main signals of orcinol in the ^1^H-NMR spectrum appear at *δ* = 3.28 (2H, *d*, *J* = 6.7 Hz), *δ* = 5.14 (1H, m); 5.04 (1H, m) assigned to H-1′; H-2′ and H-6′, respectively. In the mono-hydrated compound **26**, these signals appear with the same chemical shift, excepting H-6′ that is shifted to the lower field, *δ* = 1.80 (2H, m), due to the interaction with the hydroxyl group. A similar effect is observed for H-2′ in compound **27**, whereas in the spectra of compound **36** signals corresponding to H-1′; H-2′ and H-6′ appear at *δ* = 2.52 (2H, t, *J* = 6.68 Hz); 1.80 (2H, m) y 1.47 (2H, m), respectively.

### 2.2. In Vitro Antifungal Activity against B. cinerea

**Figure 8:**
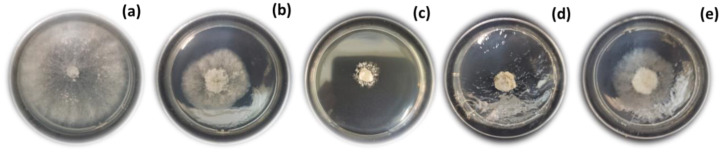
In vitro effect of geranylated phenols with one hydroxyl group in the side chain (**30**) on mycelial growth of *Botrytis cinerea* at different concentrations: (**a**) 0 µg/mL (Negative control); (**b**) 10 µg/mL; (**c**) 150 µg/mL; (**d**) 250 µg/mL; (**e**) BC-1000 at 150 µg/mL, used as positive control.

The inhibition of mycelial growth of *B. cinerea* by all geranylated phenols, with and without hydrated side chain, was evaluated by measuring colony diameters in the presence and absence of tested compounds at different concentrations. Typical results obtained for derivative **30** are shown in Figure 8. The mycelial growth in the presence of three different concentrations was measured and compared with those observed in the absence of any growth inhibitor (negative control) and in presence of a commercial fungicide (BC-1000, positive control).

From these results the percentage of inhibition was calculated and plotted as a function of concentration for each compound. Finally, fitting data to the response–activity equation provides the EC_50_ values that are tabulated in Table 1, Table 2 and Table 3

The biological activity of geranylated phenols with no hydroxyl functions in the side chain (Figure 3) are listed in Table 1.

The data in Table 1 indicate that the highest activity against *B. cinerea* is exhibited by compounds **17**, **18** and **19**. In all of them the geranyl chain is in the *ortho* position to the phenol group. Changing the hydroxyl group by a methyl group or moving the geranyl chain to the *para* position (compounds **16** and **3**, respectively), reduces the inhibition activity. In a previous work it was shown that 2-geranylhydroquinone (compound **18**) was the most active of a series of geranylated phenols with two or three hydroxyl groups, and that the activity decreases slightly by acetylation of hydroxyl groups [11]. Herein, compound **19**, with two methoxy groups, exhibits almost the same activity, suggesting that methoxy groups have no effect on activity.

On the other hand, EC_50_ values obtained for geranylated phenols with hydrated side chain are listed in Table 2 and Table 3.

The data show that the effect of side chain hydration on bioactivity depends strongly on the structure of geranylated phenols. For example, the position of –OH in monohydrated compounds **23** and **24** has almost no effect on EC_50_ values, whereas compound 27 (–OH in C-3′) is three times more active than 26 (–OH in C-7′). On the other hand, hydration in the C-3′ slightly diminishes the bioactivity from 84 (compound **18**) to 108 (compound **25**), whereas hydration in C-7′of compound **19** leads to compound **30** which is the most active geranyl derivative reported in this work. This structural change induces a five-fold change in the EC_50_ value. Finally, addition of a second hydroxyl group decreases the activity at a level that some of them are completely inactive (compares EC_50_ values of compounds **23**, **24** and **34**; **26**, **27** and **36**). The exception is compound **35** that exhibits higher activity than **25**, i.e., EC_50_ values of 54 and 108, respectively.

To get a better insight in the way that growth inhibition activity depends on the chemical structure of the geranylated phenols, and specially the hydration of the side alkyl chain, we have performed a docking molecular study.

### 2.3. Molecular Docking Study

Recently, it has been shown that the antifungal activity of a variety of compounds is mainly due to inhibition of succinate dehydrogenase (SDH) which is a component of the mitochondrial respiratory chain and the tricarboxylic acid cycle [21,22,23]. Thus, the interactions of antifungal compounds with the ubiquinone binding site have been evaluated to understand the experimental activity shown by different series of related compounds [24,25,26,27,28]. In this work, we have performed molecular docking studies of geranylated phenols carrying one (**23**–**33**), two (**34**–**38**) or no (**14**, **18**, **19**) hydroxyl groups in the side alkyl chain. Molecular coupling of these compounds to a single crystal structure of SDH (PDB code 2FBW) was carried out by using Glide software. The obtained data indicate that binding energies of these compounds to the allosteric binding site of SDH are in the range of −9.2 to −5.4 kcal mol^−1^ (Table 4). Interestingly, the most active compounds (lowest EC_50_ values) give the highest docking scores. For example, the most active geranylated phenols with one –OH in the geranyl chain are compounds **30**, **32** and **27**, which give the highest binding energies, namely −9.2 to −7.6. The same trend is obtained for di-hydrated compounds, **37** and **35**, and compounds with no –OH in the geranyl chain, **19** and **18**. In a previous study, we evaluated the antifungal activity of a series of geranylated phenols, geranylated quinones and hydrated-geranyl phenols. The results indicated that compound **14** was the most active compound and at least two times more active than the dihydrated derivative **13** [14]. Therefore, **14** was included in the molecular docking study and a docking score of −8.9 kcal mol^−1^ was obtained, which is very close to that obtained for compound **30**.

**Figure 9:**
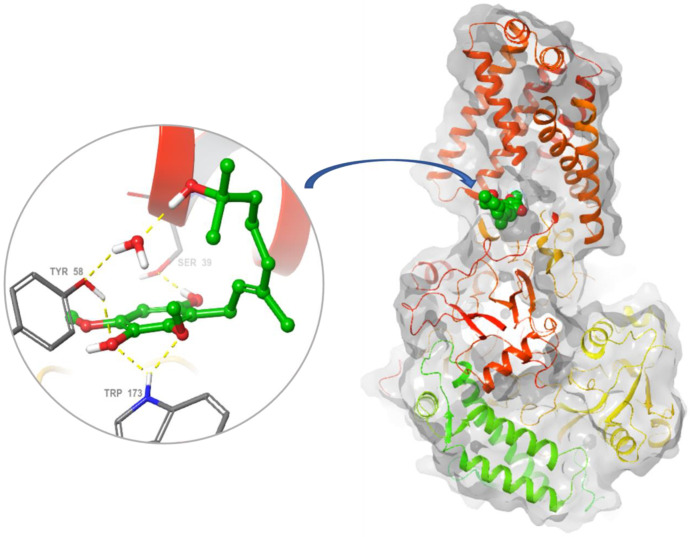
Predicted binding mode of **30** into the allosteric site of enzyme SDH (PDB 2FBW). Hydrogen bonds are represented as yellow dotted lines. (Image created using Maestro 11.8).

In the predicted pose of **30**, the most active compound, five H-bonds were identified. Two of them are formed by the hydroxyl group in position 4 with Tyr 58 and Trp 173 at 1.98 Å and 2.17 Å, respectively; the others are formed by interaction between the hydroxyl group in position 1 and Ser 39 at 1.91 Å; the methoxy group at position 5 and Trp 173 at 2.05 Å; the hydroxyl group at C7′ and Tyr 58. The latter is mediated by a water molecule located at 1.84 Å from the hydroxyl group and 2.18 Å from the Tyr 58 residue (see Figure 9). On the other hand, the side alkyl chain participates in van der Waals interactions with Pro 169, Ile 27, Ile 40, Ile 218, Trp 32, Trp 172, Hie 216, Arg 43 and Met 36.

**Figure 10:**
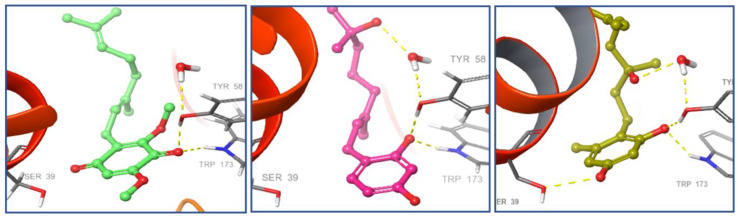
Predicted binding mode of **20**, **23** and **27** into the allosteric site of enzyme SDH (PDB 2FBW). (Image created using Maestro 11.8).

It seems that hydroxyl groups in positions 1 and 4 and in the alkyl side chain play a main role in the binding of compounds to the active site, and consequently in the antifungal activity [29]. For example, compounds **14** and **29** differ only in the nature of substituent at position 4, i.e., –OH in compound **14** and –OCH_3_ in **29** and the docking scores are completely different: −8.9 and −6.5, respectively. To address the binding mode, three compounds exhibiting different antifungal activities were tested and the interactions with amino acid residues in the SDH active site were determined (Figure 10).

The results indicate that the experimental antifungal activity correlates with the number of H-bonds formed, i.e., compound **20** (low activity, EC_50_ = 224 µg/mL) forms two H-bonds, Tyr 58 and Trp 173; compound **23** (medium activity, EC_50_ = 130 µg/mL) forms three H-bonds, Trp 173 and two with Tyr 58; compound **27** (high activity, EC_50_ = 59 µg/mL) forms four H-bonds, Ser 39, Trp 173 and two with Tyr 58. Thus, it can be concluded that functional groups with H-bond forming atoms, both in the aromatic ring and side alkyl chain, favor the binding of compounds to the allosteric site of SDH and consequently enhance their antifungal activity.

## 3. Materials and Methods

### 3.1. Chemistry

All chemicals were obtained from Merck (Darmstad, Germany) or Aldrich (St. Louis, MO, USA) and used without further purification. The ^1^H and ^13^C NMR spectra were recorded on a Bruker Avance 400 Digital NMR spectrometer, operating at 400.1 MHz for ^1^H and 100.6 MHz for ^13^C. Samples were prepared in CDCl_3_ and chemical shifts are reported in δ ppm, whereas coupling constants (J) are given in Hz. All signals were referenced to the residual peak of CHCl_3_ at δ = 7.26 ppm and δ = 77.00 ppm for ^1^H and ^13^C, respectively. Low-resolution mass spectra were obtained on a VG Micromass ZAB-2F and high-resolution mass spectra on a VG Micromass ZAB-2F at 70 eV. Pure compounds were isolated from product reaction by column chromatography (CC), using silica gel (Merck 200–300 mesh). On the other hand, the degree of reaction was tested by thin layer chromatography (TLC) and using silica gel plates (HF-254). Spots were detected on TLC by heating after spraying with H_2_SO_4_ (25% in H_2_O). Microwave syntheses were carried out in a microwave reactor (Monowave 200, Anton Parr, Graz, Austria) using sealed glass microwave containers with polyether ether ketone (PEEK) lids (capacity 10 or 30 mL).

### 3.2. Synthesis

#### 3.2.1. Microwave-Assisted Electrophilic Aromatic Substitution Reaction

Coupling of geraniol and substituted phenols was carried out in presence of BF_3_·OEt_2_ as catalyst, acetonitrile as solvent and under microwave radiation. In a typical reaction, a phenol is dissolved in acetonitrile and then an excess of geraniol is added under constant stirring. Once the mixture is homogeneous, it is located into a microwave reactor at 75 °C and 40 W of power is applied for 10 min. At the end of this period, the power is switched off and the temperature decreased to 40 °C. The reacted mixture is slowly added to water (20 mL) and then extracted with ethyl acetate (EtOAc). The organic phase was washed with aqueous NaHCO_3_ solution (10%, 20 mL) and water (3 times, 20 mL), dried over MgSO_4_ and concentrated by reduced pressure distillation. Pure compounds were obtained by column chromatography using hexane/EtOAc mixtures of increasing polarity.

##### (E)-4-(3,7-dimethylocta-2,6-dien-1-yl)-6-methylphenol (**3**)

Compound **3** was obtained by microwave-assisted coupling reaction of *o*-cresol (1 g, 9.3 mmol) and geraniol (3.0 g, 19.0 mmol). Compound **3** (497 mg, 22% yield) was obtained as a brown viscous oil. Spectroscopic data obtained for **3** were consistent with those previously reported [14].

##### (E)-2-(3,7-dimethylocta-2,6-dien-1-yl)benzene-1,4-diol (**18**)

Compound **18** was obtained by a microwave-assisted electrophilic aromatic substitution reaction, from hydroquinone (1 g, 9.0 mmol) and geraniol (2.2 g, 14.2 mmol). The compound **18** was obtained as a brown viscous oil (470 mg, 21% yield). Spectroscopic data of **18** were consistent with those previously reported [9].

##### (E)-2-(3,7-dimethylocta-2,6-dien-1-yl)benzene-1,3,5-triol (**22**)

Compound **22** was obtained by a microwave-assisted electrophilic aromatic substitution reaction, from phloroglucinol (1 g, 7.9 mmol) and geraniol (3 g, 19.5 mmol), as a brown viscous oil (416 mg, 20% yield). Spectroscopic data for 22 were consistent with those previously reported [11].

##### (E)-4-(3,7-dimethylocta-2,6-dien-1-yl)-3-methylphenol (**16**) and (E)-2-(3,7-dimethylocta-2,6-dien-1-yl)-5-methylphenol (**17**)

Microwave-assisted electrophilic aromatic substitution reaction of *m*-cresol (1 g, 9.3 mmol) and geraniol (3.0 g, 19.0 mmol) gives a reaction product from which two fractions were obtained by C.C. Fraction I: Compound **16** (226 mg, 10% yield) obtained as a brown viscous oil and Fraction II: Compound **17** (497 mg, 22% yield) obtained as a brown viscous oil. Spectroscopic data recorded for compounds **16** and **17** were consistent with those previously reported [30].

##### (E)-2-(3,7-dimethylocta-2,6-dien-1-yl)-4,5-dimethoxyphenol (**21**)

Compound **21** was obtained by microwave-assisted electrophilic aromatic substitution reaction, from 3,4-dimethoxyphenol (2 g, 12.9 mmol) and geraniol (4.5 g, 29.2 mmol). Compound **21** was obtained as a brown viscous oil (827 mg, 22% yield), and its spectroscopic data were consistent with those previously reported [30].

##### (E)-2-(3,7-dimethylocta-2,6-dien-1-yl)-3,5-dimethoxybenzene-1,4-diol (**19**)

Compound **19** was obtained by a microwave-assisted electrophilic aromatic substitution reaction, from 2,6-dimethoxyhydroquinone (1 g, 5.9 mmol) and geraniol (2.2 g, 14.2 mmol), as a brown viscous oil (396 mg, 21% yield). Compound **19**: ^1^H NMR (400 MHz, CDCl_3_) (Figure S1, Supplementary Material): δ 5.93 (s, 1H, H-6); 5.04 (m, 2H, H-2′ y H-6′), 3.96 (s, 3H, CH_3_O-C3); 3.79 (s, 3H, CH_3_O-C5); 3.15 (*d*, *J* = 7.3 Hz, 2H, H-1′); 2.03 (m, 2H, H-5′); 1.95 (m, 2H, H-4′); 1.72 (s, 3H, CH_3_-C3′); 1.65 (s, 3H, H-8′); 1.57 (s, 3H, CH_3_-C7′). ^13^C NMR (100 MHz, (CDCl_3_) (Figure S1, Supplementary Material): δ 157.2 (C-1); 154.1 (C-3); 150.2 (C-5); 137.3 (C-3′); 132.9 (C-7′); 131.4 (C-6′) 124.1 (C-2′); 128.3 (C-4); 119.7 (C-2); 106.9 (C-6); 60.9 (CH_3_O-C3); 56.4 (CH_3_O-C5); 39.7 (C-4′); 26.5 (C-5′); 25.7 (C-8′); 22.5 (C-1′); 17.6 (CH_3_-C7′); 16.1 (CH_3_-C3′). EIMS *m*/*z*: 306 [M^+^] (12), 69 (57), 118 (30), 123 (27), 169 (29), 177 (41), 182 (65), 221 (100), 235 (47), 261 (45), 304 (35); HR-EIMS [M^+^] found 306.1841, calcd for C_18_H_26_O_4_ 306.1831 (Figure S18, Supplementary Material).

#### 3.2.2. Hydration Reaction

Hydration reactions were performed by dissolving geranylated phenols in dioxane (20–40 mL) containing water (5–7 mL) and using *p*-toluenesulfonic acid polymer-bound as catalyst PTSA-PB (macroporous polymer, 30–60 mesh, 2.0–3.0 mmol/g loading, Aldrich). The reaction mixture is refluxed with constant stirring until no reaction is detected by TLC (16 to 24 h). The crude reaction product was extracted with AcOEt and the organic phase was washed with aqueous NaHCO_3_ solution (10%, 10–20 mL) and water (2 times, 20 mL), dried over MgSO_4_ and concentrated by reduced pressure distillation. Pure compounds were obtained by column chromatography using hexane/EtOAc mixtures of increasing polarity.

##### 4-(3-hydroxy-3,7-dimethyloct-6-en-1-yl)benzene-1,3-diol (**24**); (E)-4-(7-hydroxy-3,7-dimethyloct-2-en-1-yl)benzene-1,3-diol (**23**) and 4-(3,7-dihydroxy-3,7-dimethyloctyl)benzene-1,3-diol (**34**)

Compounds **23**, **24** and **34** were obtained from 4-geranylresorcinol (300 mg, 1.2 mmol) and PTSA-PB (500 mg, ≈1.5 mmol) in dioxane (20 mL) with H_2_O (5 mL). Three fractions were obtained from C.C. Fraction I: Compound **24** (29 mg, 9% yield) obtained as a brown viscous oil. ^1^H NMR (400 MHz, CDCl_3_) (Figure S4, Supplementary Material): 6.88 (*d*, *J* = 8.6 Hz, 1H, H-5), 6.32 (m, 2H, H-2, H-6), 5.11 (m, 1H, H-6′), 2.61 (m, 2H, H-1′), 2.08 (m, 2H, H-5′), 1.80 (m, 2H, H-2′), 1.60 (m, 2H, H-4′) 1.67 (s, 3H, H-8′), 1.60 (s, 3H, CH_3_-C7′), 1.28 (s, 3H, CH_3_-C3′). ^13^C NMR (100 MHz, (CDCl_3_) (Figure S4, Supplementary Material): δ 155.3 (C-3), 155.1 (C-1), 131.6 (C-7′), 130.2 (C-5), 124.2 (C-6′), 119.5 (C-4), 106.9 (C-6), 104.4 (C-2), 75.4 (C-3′), 39.3 (C-4′), 30.6 (C-2′), 25.7 (C-8′), 24.0 (CH_3_-C3′), 22.3 (C-5′), 21.1 (C-1′), 17.6 (CH_3_-C5), 16.3 (CH_3_-C7′). EIMS *m*/*z*: 246 [M^+^-H_2_O] (23), 123 (100), 161 (31), 163 (11), 177 (8); HR-EIMS [M^+^ -H_2_O] found 246.1627, calcd for C_16_H_22_O_2_ 246.1620 (Figure S21, Supplementary Material). Fraction II: Compound **34** (35 mg, 10% yield) obtained as a brown viscous oil. ^1^H NMR (400 MHz, CDCl_3_) (Figure S13, Supplementary Material): δ 6.89 (*d*, *J* = 8.2 Hz, 1H, H-5), 6.34 (*d*, *J* = 8.2 Hz, 1H, H-2), 6.27 (s, 1H, H-6), 2.66 (m, 2H, H-1′), 1.78 (m, 4H, H-2′, H-4′), 1.54 (m, 4H, H-5′, H-6′), 1.27 (s, 3H, CH_3_-C3′), 1.23 (s, 6H, H-8′, CH_3_-C7′). ^13^C NMR (100 MHz, (CDCl_3_) (Figure S13, Supplementary Material): δ 155.2 (C-1), 154.8 (C-3), 130.2 (C-5), 113.4 (C-4), 107.5 (C-6), 103.9 (C-2), 76.4 (C-3′), 71.5 (C-7′), 44.3 (C-6′), 40.3 (C-4′), 31.1 (C-2′), 29.3 (CH_3_-C7′), 29.3 (C-8′), 24.1 (CH_3_-C3′), 21.4 (C-1′), 18.3 (C-5′). EIMS *m*/*z*: 264 [M^+^ -H_2_O] (15), 123 (100), 161 (12), 163 (14), 175 (9), 177 (5); HREIMS [M^+^ -H_2_O] found 264.1723, calcd for C_16_H_24_O_3_ 264.1725 (Figure S30, Supplementary Material). Fraction III: Compound **23** (26 mg, 8% yield) obtained as a brown viscous oil. ^1^H NMR (400 MHz, CDCl_3_) (Figure S3, Supplementary Material): δ 6.92 (*d*, *J* = 8.6 Hz, 1H, H-5), 6.34 (m, 2H, H-2, H-6), 5.29 (t, *J* = 7.3 Hz, 1H, H-2′), 3.28 (*d*, *J* = 7.2 Hz, 2H, H-1′), 2.03 (t, *J* = 6.9 Hz, 2H, H-6′), 1.74 (s, 3H, CH_3_-C3′), 1.47 (m, 4H, H-4′, H-5′), 1.21 (s, 6H, H-8′, CH_3_-C7′). ^13^C NMR (100 MHz, (CDCl_3_) (Figure S3, Supplementary Material): δ 155.4 (C-1), 155.4 (C-3), 137.8 (C-3′), 130.6 (C-5), 122.5 (C-2′), 119.1 (C-4), 107.5 (C-6), 103.3 (C-2), 71.4 (C-7′), 43.2 (C-6′), 39.9 (C-4′), 29.2 (C-8′ y CH_3_-C7′), 29.1 (C-1′), 22.4 (C-5′), 16.1 (CH_3_-C3′). EIMS *m*/*z* (%): 264 [M]^+^ (3), 123 (100), 161 (25), 163 (15), 175 (18), 177 (12), 246 (15); HREIMS [M]^+^ found 264.1724, calcd for C_16_H_24_O_3_ 264.1725 (Figure S20, Supplementary Material).

##### (E)-2-(3,7-dimethylocta-2,6-dien-1-yl)-3,5-dimethoxycyclohexa-2,5-diene-1,4-dione (**20**) and (E)-2-(7-hydroxy-3,7-dimethyloct-2-en-1-yl)-3,5-dimethoxycyclohexa-2,5-diene-1,4-dione (**31**)

Compounds **20** and **31** were obtained by hydration reaction of 3-geranyl-2,4,6-trimethoxyphenol (300 mg, 1.2 mmol) in dioxane (40 mL) with 7 mL of H_2_O (7 mL) and PTSA-PB (700 mg, 3.5 mmol). Two fractions were obtained from the C.C. Fraction I: Compound **20** (122 mg, 32% yield) obtained as a brown viscous oil. ^1^H NMR (400 MHz, CDCl_3_) (Figure S2, Supplementary Material): δ 5.83 (s, 1H, H-6); 5.05 (m, 2H, H-2′ y H-6′), 3.96 (s, 3H, CH_3_O-C3); 3.79 (s, 3H, CH_3_O-C5); 3.15 (*d*, *J* = 7.3 Hz, 2H, H-1′); 2.03 (m, 2H, H-5′); 1.96 (m, 2H, H-4′); 1.72 (s, 3H, CH_3_-C3′); 1.65 (s, 3H, H-8′); 1.57 (s, 3H, CH_3_-C7′). ^13^C NMR (100 MHz, (CDCl_3_) (Figure S2, Supplementary Material): δ 187.2 (C-1); 178.4 (C-4); 157.2 (C-5); 154.1 (C-3); 137.3 (C-3′); 132.9 (C-7′); 124.1 (C-6′); 119.7 (C-2′); 106.9 (C-6); 60.9 (CH_3_O-C3); 56.4 (CH_3_O-C5); 39.7 (C-4′); 26.5 (C-5′); 25.7 (C-8′); 22.5 (C-1′); 17.6 (CH_3_-C7′); 16.1 (CH_3_-C3′). EIMS *m*/*z*: 304 [M^+^] (32), 123 (34), 183 (85), 194 (17), 221 (100), 235 (32), 26 (33), 289 (15); HR-EIMS [M^+^] found 304.1682, calcd for C_18_H_24_O_4_ 304.1675 (Figure S19, Supplementary Material). Fraction II: Compound **31** (28 mg, 7% yield) obtained as a brown viscous oil. ^1^H NMR (400 MHz, CDCl_3_) (Figure S10, Supplementary Material): δ 5.83 (s, 1H, H-6), 5.05 (t, *J* = 6.8 Hz, 1H, H-2′), 3.96 (s, 3H, CH_3_O-C3), 3.79 (s, 3H, CH_3_O-C5), 3.16 (*d*, *J* = 7.2 Hz, 2H, H-1′), 1.95 (m, 1H, H-4′), 1.72 (s, 3H, CH_3_-C3′), 1.40 (m, 2H, H-6′), 1.23 (m, 2H, H-5′), 1.20 (s, 6H, H-8′ y CH_3_-C7′). ^13^C NMR (100 MHz, (CDCl_3_) (Figure S10, Supplementary Material): δ 187.2 (C-1), 178.5 (C-4), 157.3 (C-5), 154.1 (C-3), 137.2 (C-3′), 132.8 (C-2′), 119.9 (C-2), 106.9 (C-6), 71.0 (C-7′), 61.0 (CH_3_O-C3), 56.4 (CH_3_O-C5), 43.4 (C-6′), 40.0 (C-4′), 29.2 (C-8′ y CH_3_-C7′), 22.5 (C-1′ y C-5′), 16.1 (CH_3_-C3′). EIMS *m*/*z* [M^+^] 322 (9), 149 (100), 183 (63), 194 (15), 221 (67), 305 (14); HR-EIMS [M^+^] found 322.1783, calcd for C_18_H_26_O_5_ 322.1783 (Figure S27, Supplementary Material).

##### (E)-4-(7-hydroxy-3,7-dimethyloct-2-en-1-yl)-5-methylbenzene-1,3-diol (**26**), 4-(3-hydroxy-3,7-dimethyloct-6-en-1-yl)-5-methylbenzene-1,3-diol (**27**) and 4-(3,7-dihydroxy-3,7-dimethyloctyl)-5-methylbenzene-1,3-diol (**36**)

Compounds **26, 27** and **36** were obtained from geranylorcinol (200 mg, 0.8 mmol) and PTSA-PB (400 mg, 2.0 mmol) in dioxane (30 mL) with H_2_O (5 mL), following the standard procedure described above for the hydration reaction. Three fractions were obtained by C.C. Fraction I: Compound **27** (60 mg, 28% yield) obtained as a brown viscous oil. ^1^H NMR (400 MHz, CDCl_3_) (Figure S6, Supplementary Material): δ 6.26 (s, 1H, H-6), 6.16 (s, 1H, H-2), 5.10 (m, 1H, H-6′), 4.66 (s, 1H, OH), 2.60 (m, 2H, H-1′), 2.20 (s, 3H, CH_3_-C5), 2.08 (m, 2H, H-5′), 1.80 (m, 2H, H-2′), 1.60 (m, 2H, H-4′), 1.67 (s, 3H, H-8′), 1.60 (s, 3H, CH_3_-C7′), 1.28 (s, 3H, CH_3_-C3′). ^13^C NMR (100 MHz, (CDCl_3_) (Figure S6, Supplementary Material): δ 154.7 (C-3), 153.6 (C-1), 137.4 (C-5), 131.6 (C-7′), 124.2 (C-6′), 110.4 (C-4), 106.9 (C-6), 105.4 (C-2), 75.7 (C-3′), 39.3 (C-4′), 30.4 (C-2′), 25.7 (C-8′), 24.0 (CH_3_-C3′), 22.3 (C-5′), 21.1 (C-1′), 17.6 (CH_3_-C5), 16.3 (CH_3_-C7′). EIMS *m*/*z* 260 [M^+^] (31), 123 (11), 137 (100), 175 (26), 177 (12); HR-EIMS [M^+^-H_2_O] found 260.1786, calcd for C_17_H_24_O_2_ 260.1776 (Figure S23, Supplementary Material). Fraction II: Compound **36** (36 mg, 16% yield) obtained as a brown viscous oil. ^1^H NMR (400 MHz, CDCl_3_) (Figure S15, Supplementary Material): δ 6.25 (*d*, *J* = 2.1 Hz, 1H, H-6), 6.15 (*d*, *J* = 2.1 Hz, 1H, H-2), 2.52 (t, *J* = 6.8 Hz, 2H, H-1′), 2.15 (s, 3H, CH_3_-C5), 1.80 (m, 2H, H-2′), 1.59 (m, 2H, H-4′), 1.52 (m, 4H, H-5′, H-6′), 1.26 (s, 3H, CH_3_-C3′), 1.22 (s, 6H, H-8′, CH_3_-C7′). ^13^C NMR (100 MHz, (CDCl_3_) (Figure S15, Supplementary Material): δ 154.6 (C-3), 154.4 (C-1), 138.2 (C-5), 112.2 (C-4), 108.8 (C-6), 101.5 (C-2), 77.7 (C-3′), 71.2 (C-7′), 44.1 (C-6′), 39.9 (C-4′), 31.1 (C-2′), 29.3 (CH_3_-C7′), 29.2 (C-8′), 23.9 (CH_3_-C3′), 19.4 (C-1′), 19.3 (CH_3_-C5), 18.3 (C-5′). EIMS *m*/*z* 278 [M^+^-H_2_O] (10), 137 (100), 175 (17), 177 (9), 260 (10), 278 (10); HR-EIMS [M^+^-H_2_O] found 278.1877, calcd for C_17_H_26_O_3_ 278.1882 (Figure S32, Supplementary Material). Fraction III: Compound **26** (26 mg, 12% yield) obtained as a brown viscous oil. ^1^H NMR (400 MHz, CDCl_3_) (Figure S5, Supplementary Material): δ 6.26 (*d*, *J* = 2.3 Hz, 1H, H-6), 6.21 (*d*, *J* = 2.3 Hz, 1H, H-2), 5.13 (t, *J* = 6.8 Hz, 1H, H-2′), 3.28 (*d*, *J* = 6.8 Hz, 2H, H-1′), 2.22 (s, 3H, CH_3_-C5), 2.00 (m, 2H, H-4′), 1.77 (s, 3H, CH_3_-C3′), 1.45 (m, 4H, H-5′ y H-6′), 1.21 (s, 6H, H-8′ y CH_3_-C7′). ^13^C NMR (100 MHz, (CDCl_3_) (Figure S5, Supplementary Material): δ 155.3 (C-3), 154.3 (C-1), 138.5 (C-5), 137.0 (C-3′), 122.4 (C-2′), 117.9 (C-4), 109.6 (C-6), 100.9 (C-2), 71.3 (C-7′), 43.3 (C-6′), 39.9 (C-4′), 29.2 (C-8′ y CH_3_-C7′), 25.0 (C-1′), 22.4 (C-5′), 20.1 (CH_3_-C5), 16.1 (CH_3_-C3′). EIMS *m*/*z* 260 [M^+^] (20), 123 (15), 137 (100), 175 (31), 177 (14); HR-EIMS [M^+^-H_2_O] found 260.1780, calcd for C_17_H_24_O_2_ 260.1776 (Figure S22, Supplementary Material).

##### 4-((E)-3,7-dimethylocta-2,6-dien-1-yl)-6-((e)-7-hydroxy-3,7-dimethyloct-2-en-1-yl)benzene-1,3-diol (**32**) and (E)-4-(3,7-dimethylocta-2,6-dien-1-yl)-6-(3-hydroxy-3,7-dimethyloct-6-en-1-yl)benzene-1,3-diol (**33**)

Compounds **32** and **33** were obtained from 4,6-digeranylresorcinol (1 g, 2.6 mmol) and PTSA-PB (2 g ≈ 5.5 mmol) in dioxane (50 mL) with H_2_O (8 mL), following the standard procedure described above for the hydration reaction. Two fractions were obtained by C.C. Fraction I: Compound **33** (32 mg, 3% yield) obtained as a brown viscous oil. ^1^H NMR (400 MHz, CDCl_3_) (Figure S12, Supplementary Material): δ 6.74 (s, 1H, H-5), 6.28 (s, 1H, H-2), 5.30 (t, *J* = 7.2 Hz, 1H, H-2″), 5.09 (m, 2H, H-6′, H-6″), 4.94 (s, 1H, OH), 3.26 (*d*, *J* = 7.2 Hz, 2H, H-1″), 2.65 (t, *J* = 6.8 Hz, 2H, H-1′), 2.07 (m, 8H, H-4′, H-5′, H-4″, H-5″), 1.76 (s, 3H, CH_3_-C-3″), 1.68 (s, 3H, H-8″), 1.67 (s, 3H, CH_3_-C-7″), 1.60 (s, 6H, H-8′, CH_3_-C-7′), 1.27 (s, 3H, CH_3_-C-3′). ^13^C NMR (100 MHz, (CDCl_3_) (Figure S12, Supplementary Material): δ 153.5 (C-3), 153.2 (C-1), 137.8 (C-3″), 131.9 (C-7″), 131.6 (C-7′), 130.1 (C-5), 124.3 (C-6″), 123.9 (C-6′), 122.5 (C-2′), 118.4 (C-6), 113.1 (C-4), 104.3 (C-2), 75.9 (C-3′), 39.7 (C-4′), 39.5 (C-4″), 31.2 (C-2″), 29.1 (C-1′), 26.4 (C-5′), 25.7 (C-8″), 24.2 (C-1″), 22.3 (CH_3_-C-3″), 21.4 (C-5″), 17.7 (CH_3_-C-7″), 17.6(CH_3_-C-7′), 16.1(CH_3_-C-3′). EIMS *m*/*z* 382 [M^+^-H_2_O] (71), 123 (15), 137 (47), 189 (33), 191 (46), 259 (100), 297 (67); HR-EIMS [M^+^-H_2_O] found 382.2905, calcd for C_26_H_38_O_2_ 382.2872 (Figure S29, Supplementary Material). Fraction II: Compound **32** (32 mg, 3% yield) obtained as a brown viscous oil. ^1^H-NMR (400 MHz, CDCl_3_) (Figure S11, Supplementary Material): δ = 6.78 (s, 1H, H-5), 6.33 (s, 1H, H-2), 5.30 (m, 2H, H-2′ and H-2″), 5.07 (m, 2H, H-6′ and H-6″), 3.27 (*d*, *J* = 7.2 Hz, 4H, H-1′ and H-1″), 2.06 (m, 6H, H-4′, H-5′ and H-4″), 1.76 (s, 6H, CH_3_-C-3′ and CH_3_-C-3″), 1.68 (s, 3H, H-8′), 1.60 (s, 3H, CH_3_-C-7′), 1.21 (s, 6H, H-8″ and CH_3_-C-7″). ^13^C NMR (100 MHz, (CDCl_3_) (Figure S11, Supplementary Material): δ 155.7 (C-1 and C-3), 136.9 (C-3″), 134.8 (C-3′), 130.5 (C-7′), 126.6 (C-5), 124.1 (C-6′), 123.7 (C-2″), 122.6 (C-2′), 117.8 (C-6 and C-4), 105.6 (C-2), 70.5 (C-7″), 43.5 (C-6″), 40.2 (C-4″), 39.7 (C-4’), 29.3 (C-8″ and CH_3_-C-7″), 27.3 (C-1′ and C-1″), 26.1 (C-5′), 25.4 (C-8′), 22.6 (C-5″), 17.4 (CH_3_-C-7′), 16.3 (CH_3_-C-3″), 15.7 (CH_3_-C-3′). EIMS *m*/*z* 400 [M^+^] (4), 137 (20), 148 (100), 177 (29), 189 (17), 191 (21), 259 (37), 297 (62), 382 (15); HR-EIMS [M^+^ -H_2_O] found 382.2869, calcd for C_26_H_38_O_2_ 382.2872 (Figure S28, Supplementary Material).

##### (E)-4-(7-hydroxy-3,7-dimethyloct-2-en-1-yl)-2,3-dimethoxyphenol (**28**)

Compound **28** was obtained by hydration of 4-geranyl-2,3-dimethoxyphenol (250 mg, 0.9 mmol) in dioxane (30 mL) with H_2_O (5 mL) in presence of PTSA-PB (500 mg ≈ 1.5 mmol). Compound **28** was obtained as a brown viscous oil (32 mg, 12% yield). ^1^H NMR (400 MHz, CDCl_3_) (Figure S7, Supplementary Material): δ 6.76 (*d*, *J* = 8.3 Hz, 1H, H-5), 6.64 (*d*, *J* = 8.3 Hz, 1H, H-6), 5.72 (s, 1H, OH), 5.25 (t, *J* = 7.2 Hz, 1H, H-2′), 3.91 (s, 3H, CH_3_O-C2), 3.83 (s, 3H, CH_3_O-C3), 3.26 (*d*, *J* = 7.3 Hz, 2H, H-1′), 2.02 (t, *J* = 6.8 Hz, 2H, H-6′), 1.70 (s, 3H, CH_3_-C3′), 1.46 (m, 4H, H-4′, H-5′), 1.20 (s, 6H, H-8′ y CH_3_-C7′). ^13^C NMR (100 MHz, (CDCl_3_) (Figure S7, Supplementary Material): δ 150.7 (C-2), 147.8 (C-1), 139.9 (C-3), 135.6 (C-3′), 126.9 (C-4), 124.2 (C-5), 123.3 (C-2′), 110.2 (C-6), 71.1 (C-7′), 60.7 (CH_3_O-C2), 60.4 (CH_3_O-C3), 43.5 (C-6′), 40.1 (C-4′), 29.3 (C-8′ y CH_3_-C3′), 27.8 (C-1′), 22.6 (C-5′), 16.1 (CH_3_-C3′). EIMS *m*/*z* 308 [M^+^] (18), 167 (100), 207 (11), 219 (24), 290 (11); HR-EIMS [M^+^] found 308.1993, calcd for C_18_H_28_O_4_ 308.1988 (Figure S24, Supplementary Material).

##### (E)-2-(7-hydroxy-3,7-dimethyloct-2-en-1-yl)-4,5-dimethoxyphenol (**29**) and 8-(2-hydroxy-4,5-dimethoxyphenyl)-2,6-dimethyloctane-2,6-diol (**37**)

Compounds **29** and **37** were obtained by the hydration reaction described above, from 6-geranyl-4,5-dimethoxyphenol (300 mg, 1.0 mmol) in polyethylene glycol 400 (7 mL) with H_2_O (6 mL) and PTSA-PB (300 mg ≈ 1.0 mmol). Two fractions were obtained by C.C. Fraction I: Compound **29** (48 mg, 15% yield) obtained as a brown viscous oil. ^1^H NMR (400 MHz, CDCl_3_) (Figure S8, Supplementary Material): δ 6.62 (s, 1H, H-3), 6.45 (s, 1H, H-6), 5.31 (m, 1H, H-2′), 3.82 (s, 6H, CH_3_O-C4 y CH_3_O-C5), 3.29 (*d*, 2H, H-1′), 2.06 (m, 2H, H-4′), 1.77 (s, 3H, CH_3_-C3′) 1.50 (m, 4H, H-5′ y H-6′); 1.21 (s, 6H, CH_3_-C7′ y H-8′). ^13^C NMR (100 MHz, (CDCl_3_) (Figure S8, Supplementary Material): δ 148.3 (C-1), 148.3 (C-5), 142.9 (C-4), 138.3 (C-3′), 122.1 (C-2′), 117.3 (C-2), 113.7 (C-3), 101.2 (C-6), 70.1 (C-7′), 56.7 (CH_3_O-C5), 56.0 (CH_3_O-C4), 43.3 (C-6′), 40.0 (C-4′), 29.5 (C-1′), 29.3 (C-8′ y CH_3_-C7′), 22.5 (C5′), 16.2 (CH_3_-C3′). EIMS *m*/*z* 308 [M^+^] (8), 167 (100), 205 (57), 290 (19); HR-EIMS [M^+^] found 308.1982, calcd for C_18_H_28_O_4_ 308.1988 (Figure S25, Supplementary Material). Fraction II: Compound **37** (54 mg, 16% yield) obtained as a brown viscous oil. ^1^H NMR (400 MHz, CDCl_3_) (Figure S16, Supplementary Material): δ 6.54 (s, 1H, H-6′), 6.36 (s, 1H, H-3′), 3.81 (s, 6H, CH_3_O-C4′ y CH_3_O-C5′), 2.66 (m, 2H, H-8), 1.78 (m, 2H, H-7), 1.58 (m, 2H, H-5), 1.47 (m, 4H, H-3 y H-4), 1.26 (s, 3H, CH_3_-C6); 1.22 (s, 6H, CH_3_-C2 y H-1). ^13^C NMR (100 MHz, (CDCl_3_) (Figure S16, Supplementary Material): δ 148.4 (C-2′), 147.6 (C-4′), 142.6 (C-5′), 112.3 (C-1′), 111.4 (C-6′), 101.3 (C-3′), 75.8 (C-6), 71.0 (C-2), 56.5 (CH_3_O-C5′), 55,8 (CH_3_O-C4′), 44.2 (C-3), 40.2 (C-5), 31.1 (C-7), 29.4 (C-1), 29.3 (CH_3_-C2), 23.9 (C-8), 21.7 (CH_3_-C6), 18.4 (C4). EIMS *m*/*z* 308 (M^+^ -H_2_O, 21), 167 (100), 205 (8), 290 (8); HR-EIMS [M^+^ -H_2_O] found 308.1982, calcd for C_18_H_28_O_4_ 308.1988 (Figure S33, Supplementary Material).

##### (E)-2-(7-hydroxy-3,7-dimethyloct-2-en-1-yl)-3,5-dimethoxybenzene-1,4-diol (**30**)

Compound **30** was obtained by the hydration reaction described above, from 2-geranyl-3,5-dimethoxhydroquinone (200 mg, 0.7 mmol) in dioxane (30 mL) and H_2_O (5 mL) and PTSA-PB (300 mg ≈ 1.0 mmol). Compound **30** was obtained as a brown viscous oil (66 mg, 31% yield). ^1^H NMR (400 MHz, CDCl_3_) (Figure S9, Supplementary Material): δ 5.92 (s, 1H, H-6); 5.04 (m, 1H, H-2′), 3.97 (s, 3H, CH_3_O-C3); 3.78 (s, 3H, CH_3_O-C5); 3.15 (*d*, *J* = 7.2 Hz, 2H, H-1′); 1.94 (m, 2H, H-4′); 1.72 (s, 3H, CH_3_-C3′) 1.50 (m, 4H, H-5′ y H-6′); 1.20 (s, 6H, CH_3_-C7′ y H-8′). ^13^C NMR (100 MHz, (CDCl_3_) (Figure S9, Supplementary Material): δ 157.3 (C-1); 154.1 (C-3); 150.7 (C-5); 137.2 (C-3′); 132.7 (C-4); 120.0 (C-2′); 114.2 (C-2); 106.9 (C-6); 70.9 (C-7′); 60.9 (CH_3_O-C3); 56.3 (CH_3_O-C5); 43.4 (C-6′); 40.0 (C-4′); 29.2 (C-8′ y CH_3_-C7′); 22.5 (C-1′ and C-5′); 16.0 (CH_3_-C3′). EIMS *m*/*z* 324 [M^+^] (4), 109 (11), 123 (13), 139 (14), 153 (12), 170 (12), 183 (93), 194 (23), 207 (20), 221 (100), 233, (40), 248 (23), 261 (18), 304 (19), 307 (13), 322 (19); HR-EIMS [M^+^] found 324.1936, calcd for C_18_H_28_O_5_ 324.1937 (Figure S26, Supplementary Material).

##### 8-(2-hydroxy-3,4,5-trimethoxyphenyl)-2,6-dimethyloctane-2,6-diol (**38**)

Compound **38** was obtained by the hydration reaction described above, from 6-geranyl-2,3,4-trimethoxphenol (100 mg, 0.3 mmol) and PTSA-PB (200 mg ≈ 0.6 mmol) in dioxane (20 mL) with H_2_O (4 mL). Compound **38** was obtained as a brown viscous oil (40 mg, 36% yield). ^1^H NMR (400 MHz, CDCl_3_) (Figure S17, Supplementary Material): δ 6.34 (s, 1H, H-6′), 3.85 (s, 3H, CH_3_O-C4′), 3.85 (s, 3H, CH_3_O-C3′), 3.78 (s, 3H, CH_3_O-C5′), 2.70 (m, 2H, H-8), 1.78 (m, 2H, H-7), 1.63 (m, 2H, H-4), 1.48 (m, 4H, H-3 y H-5), 1.29 (s, 3H, CH_3_-C6), 1.21 (s, 6H, H-1, CH_3_-C2 y H-1). ^13^C NMR (100 MHz, (CDCl_3_) (Figure S17, Supplementary Material): δ 146.1 (C-5′), 142.6 (C-4′), 141.7 (C-2′), 141.5 (C-3′), 116.1 (C-1′), 107.2 (C-6′), 75.9 (C-6), 71.0 (C-2), 61.3 (CH_3_O-C4′), 60.9 (CH_3_O-C3′), 56.4 (CH_3_O-C5′), 44.2 (C-3), 40.4 (C-5), 31.1 (C-7), 29.4 (CH_3_-C2), 29.2 (C-1), 23.7 (CH_3_-C6), 22.3 (C-8), 19.5 (C-4). EIMS *m*/*z* 338 [M^+^ -H_2_O] (17), 197 (100), 320 (9); HR-EIMS [M^+^ -H_2_O] found 338.2107, calcd for C_19_H_30_O_5_ 338.2093 (Figure S34, Supplementary Material).

##### 2-(3-hydroxy-3,7-dimethyloct-6-en-1-yl)benzene-1,4-diol (**25**) and 2-(3,7-dihydroxy-3,7-dimethyloctyl)benzene-1,4-diol (**35**)

Compound **35** was obtained by the hydration reaction described above, from 2-geranylhydroquinone (300 mg, 1.2 mmol) in polyethylene glycol 400 (7 mL) and H_2_O (6 mL) and PTSA-PB (300 mg ≈ 1.0 mmol). Two fractions were obtained by C.C. Fraction I: Compound **25** was obtained as a brown viscous oil (39 mg, 12% yield). The spectroscopic data for **25** were consistent with those previously reported [31,32] Fraction II: Compound **35** was obtained as a brown viscous oil (35 mg, 10% yield). ^1^H NMR (400 MHz, CDCl_3_) (Figure S14, Supplementary Material): δ 6.65 (*d*, *J* = 8.6 Hz, 1H, H-6), 6.56 (m, 2H, H-3 y H-5), 2.70 (m, 2H, H-1′), 1.76 (m, 2H, H-2′), 1.58 (m, 2H, H-4′), 1.47 (m, 4H, H-5′ y H-6′), 1.25 (s, 3H, CH_3_-C3′); 1.22 (s, 6H, CH_3_-C7′ y H-8′). ^13^C NMR (100 MHz, (CDCl_3_) (Figure S14, Supplementary Material): δ 148.6 (C-4), 147.7 (C-1), 121.9 (C-2), 117.8 (C-3), 115.4 (C-6), 114.5 (C-5), 75.8 (C-3′), 71.3 (C-7′), 44.2 (C-6′), 40.1 (C-4′), 31.0 (C-2′), 29.4 (C-8′), 29.2 (CH_3_-C7′), 24.0 (C-1′), 22.3 (CH_3_-C3′), 18.4 (C5′). EIMS *m*/*z* 264 [M^+^ -H_2_O] (34), 123 (100), 161 (25), 163 (30), 175 (19), 246 (19); HR-EIMS [M^+^ -H_2_O] found 264.1720, calcd for C_16_H_24_O_3_ 264.1725 (Figure S31, Supplementary Material).

### 3.3. Antifungal Assays

Antifungal activity of all synthesized compounds was evaluated by determining mycelial growth inhibition of *B. cinerea* in the radial growth test. Assayed compounds in ethanol solution were added to potato dextrose agar (PDA) at 50 °C, reaching final concentrations in the range 10 to 250 ppm. The medium with dissolved geranylated phenols was poured into 50 mm diameter Petri dishes. After solidification, Petri dishes were inoculated with 4 mm diameter agar discs with thin mycelium of *B. cinerea*. Negative control containing only PDA culture medium, and positive control with BC-1000 at the same concentrations of tested compounds were prepared. Three replicates were made for each treatment. All dishes were incubated at 23 °C under 16 h light/8 h dark photoperiod. After 3 days, the mycelial growth diameter was measured and the percentage of mycelial inhibition (%I) was calculated. These values were plotted against fungicide concentration and fitted to a dose–response equation. This fitting gives EC_50_, the concentration at which mycelial growth is inhibited to 50% as compared to the negative control. Plotting of the data, fitting and EC_50_ calculation were carried out with Origin 8.0. Significant differences were evaluated with a two-way analysis of variance (Tukey’s test; *p* < 0.05).

### 3.4. Molecular Docking

The X-ray coordinates of SDH enzyme in complex with carboxin were obtained from the RCSB PDB database (PDB code 2FBW). PDB structures for docking were prepared using the Protein Preparation Wizard (Schrodinger, LLC, New York, NY, USA, 2018) that is accessible within the Maestro program (Maestro, version 11.8; Schrodinger, LLC: New York, NY, USA, 2018). Three-dimensional structures of ligands to be docked were generated and prepared using LigPrep as implemented in Maestro 11.5 (LigPrep, Schrodinger, LLC: New York, NY, USA, 2018). Docking of ligands was carried out using Glide program (Schrödinger, LLC, New York, NY, USA, 2021). Details used for sampling of group orientations, optimization of receptor, minimization on the ligand–protein complexes, docking of compounds and ligand poses have been given elsewhere [33,34]. Briefly, GlideScore—a modified version of ChemScore—was used to estimate binding affinity and rank ligands. Ligands were docked using the extra precision mode (Glide XP), whereas XP Pose Rank was used to select the best-docked pose for each ligand.

## 4. Conclusions

Geranylated phenols with different functional groups in the aromatic ring were synthesized by direct coupling of phenol and geraniol under microwave irradiation. Yields of 21–32% were obtained in reduced reaction time (10 min). Hydration of geranylated phenols, using *p*-toluenesulfonic acid bound to a polymer matrix as catalyst, leads to compounds with one or two hydroxyl groups in the side alkyl chain with yields ranging from 10 to 36%.

The mycelium growth inhibition of B. cinerea induced by all synthesized geranylated phenols has been studied and EC_50_ values have determined for all tested compounds. The data indicate that activity depends on the structure of the phenol ring and hydration degree of the side alkyl chain. The most active compound is compound 30, which has two hydroxyl groups in positions 1 and 4 and a mono hydrated side alkyl chain. A molecular docking study shows that binding energies of these compounds to the allosteric binding site of SDH are in the range of −9.2 to −5.4 kcal mol^−1^. The results suggest that hydroxyl groups in positions 1 and 4 and in the side alkyl chain are important in the binding of compounds to the active site, and that the experimental antifungal activity correlates with the number of H-bonds that can be formed in the binding site.

## Figures and Tables

**Table 1:** Effect of chemical structure on the growth inhibition activity of geranylated phenols with no hydroxyl groups in the side chain.

Geranylated Compounds	EC_50_, µg/mL ± SD ^a^
3	>300 (NA)
16	136 ± 1.56
17	89 ± 2.00
18	84 ± 1.84
19	78 ± 2.45
20	224 ± 4.56
21	181 ± 3.62
22	276 ± 5.80

^a^ SD, standard deviation.

**Table 2:** Effect of chemical structure on the growth inhibition activity of geranylated phenols with one hydroxyl group in the side chain. Compounds **23**, **26**, **28**–**32** are hydroxylated in C-7′, whereas **24**, **25**, **27**, **33** have the hydroxyl group in C-3′.

Geranylated Compounds	EC_50_, µg/mL ± SD ^a^
23	130 ± 2.25
24	134 ± 4.30
25	108 ± 1.70
26	162 ± 3.24
27	60 ± 1.20
28	>300
29	217 ± 6.54
30	17 ± 1.35
31	>300
32	48 ± 0.80
33	151 ± 3.20

^a^ SD, standard deviation.

**Table 3:** Effect of chemical structure on the growth inhibition activity of geranylated phenols with two hydroxyl groups in the side chain.

Geranylated Compounds	EC_50_, µg/mL ± SD ^a^
**34**	>300
**35**	54 ± 0.75
**36**	>300
**37**	217 ± 3.46
**38**	123 ± 2.65

^a^ SD, standard deviation.

**Table 4:** Binding energies, obtained from molecular docking studies and antifungal activities against *B. cinerea* (EC_50_ values) of geranylated phenols with one (14, 23–33), two (34–38) or none (18, 19, 21) hydroxyl groups in the side alkyl chain. CBE is used for comparison ^1^.

One Hydroxyl Group			Two Hydroxyl Group			No Hydroxyl Group		
**ID**	EC_50_, µg/mL	Docking Score	ID	EC_50_, µg/mL	Docking Score	ID	EC_50_, µg/mL	Docking Score
**30**	17	−9.2	**37**	217	−7.4	**19**	78	−8.1
**14**	-	−8.9	**35**	54	−7.0	**18**	84	−7,1
**32**	48	−8.8	**38**	123	−6.7	**21**	181	−6.9
**27**	60	−7.6	**34**	>300	−6.3	**20**	224	−5.4
**25**	108	−6.9	**36**	>300	−6.2			
**24**	134	−6.8						
**26**	162	−6.8						
**29**	217	−6.5						
**33**	151	−6.4						
**28**	>300	−6.1						
**23**	130	−6.0						
**31**	>300	−5.6						

^1^ CBE: 2-methyl-n-phenyl-5,6-dihydro-1,4-oxathiine-3-carboxamide, ligand crystalized in SDH (2FBW). Docking score: −7.2.

## Data Availability

The data presented in this study are available in the Supplementary Material.

## Supplementary Materials

The following are available online: Figure S1: NMR spectra of (E)-2-(3,7-dimethylocta-2,6-dien-1-yl)-3,5-dimethoxybenzene-1,4-diol (**19**); Figure S2: NMR spectra of (E)-2-(3,7-dimethylocta-2,6-dien-1-yl)-3,5-dimethoxycyclohexa-2,5-diene-1,4-dione (**20**); Figure S3: NMR spectra of (E)-4-(7-hydroxy-3,7-dimethyloct-2-en-1-yl)benzene-1,3-diol (**23**); Figure S4: NMR spectra of 4-(3-hydroxy-3,7-dimethyloct-6-en-1-yl)benzene-1,3-diol (**24**); Figure S5: NMR spectra of (E)-4-(7-hydroxy-3,7-dimethyloct-2-en-1-yl)-5-methylbenzene-1,3-diol (**26**); Figure S6: NMR spectra of 4-(3-hydroxy-3,7-dimethyloct-6-en-1-yl)-5-methylbenzene-1,3-diol (**27**); Figure S7: NMR spectra of (E)-4-(7-hydroxy-3,7-dimethyloct-2-en-1-yl)-2,3-dimethoxyphenol (**28**); Figure S8: NMR spectra of (E)-2-(7-hydroxy-3,7-dimethyloct-2-en-1-yl)-4,5-dimethoxyphenol (**29**); Figure S9: NMR spectra of (E)-2-(7-hydroxy-3,7-dimethyloct-2-en-1-yl)-3,5-dimethoxybenzene-1,4-diol (**30**); Figure S10: NMR spectra of (E)-2-(7-hydroxy-3,7-dimethyloct-2-en-1-yl)-3,5-dimethoxycyclohexa-2,5-diene-1,4-dione (**31**); Figure S11: NMR spectra of 4-((E)-3,7-dimethylocta-2,6-dien-1-yl)-6-((E)-7-hydroxy-3,7-dimethyloct-2-en-1-yl)benzene-1,3-diol (**32**); Figure S12: NMR spectra of (E)-4-(3,7-dimethylocta-2,6-dien-1-yl)-6-(3-hydroxy-3,7-dimethyloct-6-en-1-yl)benzene-1,3-diol (**33**); Figure S13: NMR spectra of (E)-4-(7-hydroxy-3,7-dimethyloct-2-en-1-yl)benzene-1,3-diol (**23**) and 4-(3,7-dihydroxy-3,7-dimethyloctyl)benzene-1,3-diol (**34**); Figure S14: NMR spectra of 2-(3,7-dihydroxy-3,7-dimethyloctyl)benzene-1,4-diol (**35**); Figure S15: NMR spectra of 4-(3,7-dihydroxy-3,7-dimethyloctyl)-5-methylbenzene-1,3-diol (**36**); Figure S16: NMR spectra of 8-(2-hydroxy-4,5-dimethoxyphenyl)-2,6-dimethyloctane-2,6-diol (**37**); Figure S17: NMR spectra of 8-(2-hydroxy-3,4,5-trimethoxyphenyl)-2,6-dimethyloctane-2,6-diol (**38**); Figure S18: HRMS (EI+) spectrum of (E)-2-(3,7-dimethylocta-2,6-dien-1-yl)-3,5-dimethoxybenzene-1,4-diol (**19**); Figure S19: HRMS (EI+) spectrum of (E)-2-(3,7-dimethylocta-2,6-dien-1-yl)-3,5-dimethoxycyclohexa-2,5-diene-1,4-dione (**20**); Figure S20: HRMS (EI+) spectrum of (E)-4-(7-hydroxy-3,7-dimethyloct-2-en-1-yl)benzene-1,3-diol (**23**); Figure S21: HRMS (EI+) spectrum of 4-(3-hydroxy-3,7-dimethyloct-6-en-1-yl)benzene-1,3-diol (**24**); Figure S22: HRMS (EI+) spectrum of (E)-4-(7-hydroxy-3,7-dimethyloct-2-en-1-yl)-5-methylbenzene-1,3-diol (**26**); Figure S23: HRMS (EI+) spectrum of 4-(3-hydroxy-3,7-dimethyloct-6-en-1-yl)-5-methylbenzene-1,3-diol (**27**); Figure S24: HRMS (EI+) spectrum of (E)-4-(7-hydroxy-3,7-dimethyloct-2-en-1-yl)-2,3-dimethoxyphenol (**28)**; Figure S25: HRMS (EI+) spectrum of (E)-2-(7-hydroxy-3,7-dimethyloct-2-en-1-yl)-4,5-dimethoxyphenol (**29**); Figure S26: HRMS (EI+) spectrum of (E)-2-(7-hydroxy-3,7-dimethyloct-2-en-1-yl)-3,5-dimethoxybenzene-1,4-diol (**30**); Figure S27: HRMS (EI+) spectrum of (E)-2-(7-hydroxy-3,7-dimethyloct-2-en-1-yl)-3,5-dimethoxycyclohexa-2,5-diene-1,4-dione (**31**); Figure S28: HRMS (EI+) spectrum of 4-((E)-3,7-dimethylocta-2,6-dien-1-yl)-6-((E)-7-hydroxy-3,7-dimethyloct-2-en-1-yl)benzene-1,3-diol (**32**); Figure S29: HRMS (EI+) spectrum of (E)-4-(3,7-dimethylocta-2,6-dien-1-yl)-6-(3-hydroxy-3,7-dimethyloct-6-en-1-yl)benzene-1,3-diol (**33**); Figure S30: HRMS (EI+) spectrum of 4-(3,7-dihydroxy-3,7-dimethyloctyl)benzene-1,3-diol (**34**); Figure S31: HRMS (EI+) spectrum of 2-(3,7-dihydroxy-3,7-dimethyloctyl)benzene-1,4-diol (**35**); Figure S32: HRMS (EI+) spectrum of 4-(3,7-dihydroxy-3,7-dimethyloctyl)-5-methylbenzene-1,3-diol (**36**); Figure S33: HRMS (EI+) spectrum of 8-(2-hydroxy-4,5-dimethoxyphenyl)-2,6-dimethyloctane-2,6-diol (**37**); Figure S34: HRMS (EI+) spectrum of 8-(2-hydroxy-3,4,5-trimethoxyphenyl)-2,6-dimethyloctane-2,6-diol (**38**).

## Sample Availability

Samples of the compounds are not available from the authors.
