# Supplementary material for: Synthesis and Fungicidal Activity of Hydrated Geranylated Phenols against Botrytis cinerea
Source: Molecules. 2021 Nov 11;26(22):6815. doi: 10.3390/molecules26226815 (PMC8620067; doi:10.3390/molecules26226815)
Supplement: Supplementary file 1 [file molecules-26-06815-s001.zip › molecules-1447647-supplementary.pdf]

molecules

MDPI

Type of the Paper (Article)

Synthesis and Fungicidal Activity of Hydrated Geranylated Phenols Against *Botrytis cinerea*

Mauricio Soto [1,2], Ana Estevez-Braun[2], Ángel Amesty[2], Julia Kluepfel[3], Susana Restrepo[1], Katy Díaz[1], Luis Espinoza[1], Andrés F. Olea [4,*] and Lautaro Taborga [1,*]

[1] Departamento de Química, Universidad Técnica Federico Santa María, Av. España No.1680, Valparaíso 2340000, Chile; mauricio.sotoc.13@sansano.usm.cl (M.S.); katy.diaz@usm.cl (K.D.) lautaro.taborga@usm.cl (L.T.)

[2] Instituto Universitario de Bio-Orgánica Antonio González (CIBICAN), Departamento de Química Orgánica, Universidad de La Laguna, Av. Astrofísico Fco. Sánchez 2. 38206, La Laguna, Tenerife, España

[3] Department of Chemistry, Technical University of Munich, Lichtenberg str. 4, 85748 Garching, Germany.

[4] Grupo de Química y Bioquímica Aplicada en Biotecnología, Instituto de Ciencias Químicas Aplicadas, Facultad de Ingeniería, Universidad Autónoma de Chile, El Llano Subercaseaux 2801, Santiago 8900000, Chile; andres.olea@uautonoma.cl

* Correspondence: andres.olea@uautonoma.cl; lautaro.taborga@usm.cl (L.T.); Tel.: +56-32-265-2840 (L.T.)

Received: date; Accepted: date; Published: date

Supplementary Materials: The following are available online at www.mdpi.com/xxx/s1, **Figure S1**: NMR spectra of (E)-2-(3,7-dimethylocta-2,6-dien-1-yl)-3,5-dimethoxybenzene-1,4-diol (**19**); **Figure S2**: NMR spectra of (E)-2-(3,7-dimethylocta-2,6-dien-1-yl)-3,5-dimethoxycyclohexa-2,5-diene-1,4-dione (**20**); **Figure S3:** NMR spectra of (E)-4-(7-hydroxy-3,7-dimethyloct-2-en-1-yl)benzene-1,3-diol (**23**); **Figure S4:** NMR spectra of 4-(3-hydroxy-3,7-dimethyloct-6-en-1-yl)benzene-1,3-diol (**24**); **Figure S5:** NMR spectra of (E)-4-(7-hydroxy-3,7-dimethyloct-2-en-1-yl)-5-methylbenzene-1,3-diol (**26**); **Figure S6:** NMR spectra of 4-(3-hydroxy-3,7-dimethyloct-6-en-1-yl)-5-methylbenzene-1,3-diol (**27**); **Figure S7:** NMR spectra of (E)-4-(7-hydroxy-3,7-dimethyloct-2-en-1-yl)-2,3-dimethoxyphenol (**28**); **Figure S8:** NMR spectra of (E)-2-(7-hydroxy-3,7-dimethyloct-2-en-1-yl)-4,5-dimethoxyphenol (**29**); **Figure S9:** NMR spectra of (E)-2-(7-hydroxy-3,7-dimethyloct-2-en-1-yl)-3,5-dimethoxybenzene-1,4-diol (**30**); **Figure S10:** NMR spectra of (E)-2-(7-hydroxy-3,7-dimethyloct-2-en-1-yl)-3,5-dimethoxycyclohexa-2,5-diene-1,4-dione (**31**); **Figure S11:** NMR spectra of 4-((E)-3,7-dimethylocta-2,6-dien-1-yl)-6-((E)-7-hydroxy-3,7-dimethyloct-2-en-1-yl)benzene-1,3-diol (**32**); **Figure S12:** NMR spectra of (E)-4-(3,7-dimethylocta-2,6-dien-1-yl)-6-(3-hydroxy-3,7-dimethyloct-6-en-1-yl)benzene-1,3-diol (**33**); **Figure S13:** NMR spectra of (E)-4-(7-hydroxy-3,7-dimethyloct-2-en-1-yl)benzene-1,3-diol (**23**); and 4-(3,7-dihydroxy-3,7-dimethyloctyl)benzene-1,3-diol (**34**); **Figure S14:** NMR spectra of 2-(3,7-dihydroxy-3,7-dimethyloctyl)benzene-1,4-diol (**35**); **Figure S15:** NMR spectra of 4-(3,7-dihydroxy-3,7-dimethyloctyl)-5-methylbenzene-1,3-diol (**36**); **Figure S16:** NMR spectra of 8-(2-hydroxy-4,5-dimethoxyphenyl)-2,6-dimethyloctane-2,6-diol (**37**); **Figure S17:** NMR spectra of 8-(2-hydroxy-3,4,5-trimethoxyphenyl)-2,6-dimethyloctane-2,6-diol (**38**); **Figure S18**: HRMS (EI+) spectrum of (E)-2-(3,7-dimethylocta-2,6-dien-1-yl)-3,5-dimethoxybenzene-1,4-diol (**19**); **Figure S19**: HRMS (EI+) spectrum of (E)-2-(3,7-dimethylocta-2,6-dien-1-yl)-3,5-dimethoxycyclohexa-2,5-diene-1,4-dione (**20**); **Figure S20**: HRMS (EI+) spectrum of (E)-4-(7-hydroxy-3,7-dimethyloct-2-en-1-yl)benzene-1,3-diol (**23**); **Figure S21**: HRMS (EI+) spectrum of 4-(3-hydroxy-3,7-dimethyloct-6-en-1-yl)benzene-1,3-diol (**24**); **Figure S22**: HRMS (EI+) spectrum of (E)-4-(7-hydroxy-3,7-dimethyloct-2-en-1-yl)-5-methylbenzene-1,3-diol (**26**); **Figure S23**: HRMS (EI+) spectrum of 4-(3-hydroxy-3,7-dimethyloct-6-en-1-yl)-5-methylbenzene-1,3-diol (**27**); **Figure S24**: HRMS (EI+) spectrum of (E)-4-(7-hydroxy-3,7-dimethyloct-2-en-1-yl)-2,3-dimethoxyphenol (**28**); **Figure S25**: HRMS (EI+) spectrum of (E)-2-(7-hydroxy-3,7-dimethyloct-2-en-1-yl)-4,5-dimethoxyphenol (**29**); **Figure S26**: HRMS (EI+)

spectrum of (E)-2-(7-hydroxy-3,7-dimethyloct-2-en-1-yl)-3,5-dimethoxybenzene-1,4-diol (**30**); **Figure S27**: HRMS (EI^+) spectrum of (E)-2-(7-hydroxy-3,7-dimethyloct-2-en-1-yl)-3,5-dimethoxycyclohexa-2,5-diene-1,4-dione (**31**); **Figure S28**: HRMS (EI^+) spectrum of 4-((E)-3,7-dimethylocta-2,6-dien-1-yl)-6-((E)-7-hydroxy-3,7-dimethyloct-2-en-1-yl)benzene-1,3-diol (**32**); **Figure S29**: HRMS (EI^+) spectrum of (E)-4-(3,7-dimethylocta-2,6-dien-1-yl)-6-(3-hydroxy-3,7-dimethyloct-6-en-1-yl)benzene-1,3-diol (**33**); **Figure S30**: HRMS (EI^+) spectrum of 4-(3,7-dihydroxy-3,7-dimethyloctyl)benzene-1,3-diol (**34**); **Figure S31**: HRMS (EI^+) spectrum of 2-(3,7-dihydroxy-3,7-dimethyloctyl)benzene-1,4-diol (**35**); **Figure S32**: HRMS (EI^+) spectrum of 4-(3,7-dihydroxy-3,7-dimethyloctyl)-5-methylbenzene-1,3-diol (**36**); **Figure S33**: HRMS (EI^+) spectrum of 8-(2-hydroxy-4,5-dimethoxyphenyl)-2,6-dimethyloctane-2,6-diol (**37**); **Figure S34**: HRMS (EI^+) spectrum of 8-(2-hydroxy-3,4,5-trimethoxyphenyl)-2,6-dimethyloctane-2,6-diol (**38**).

^1H-NMR

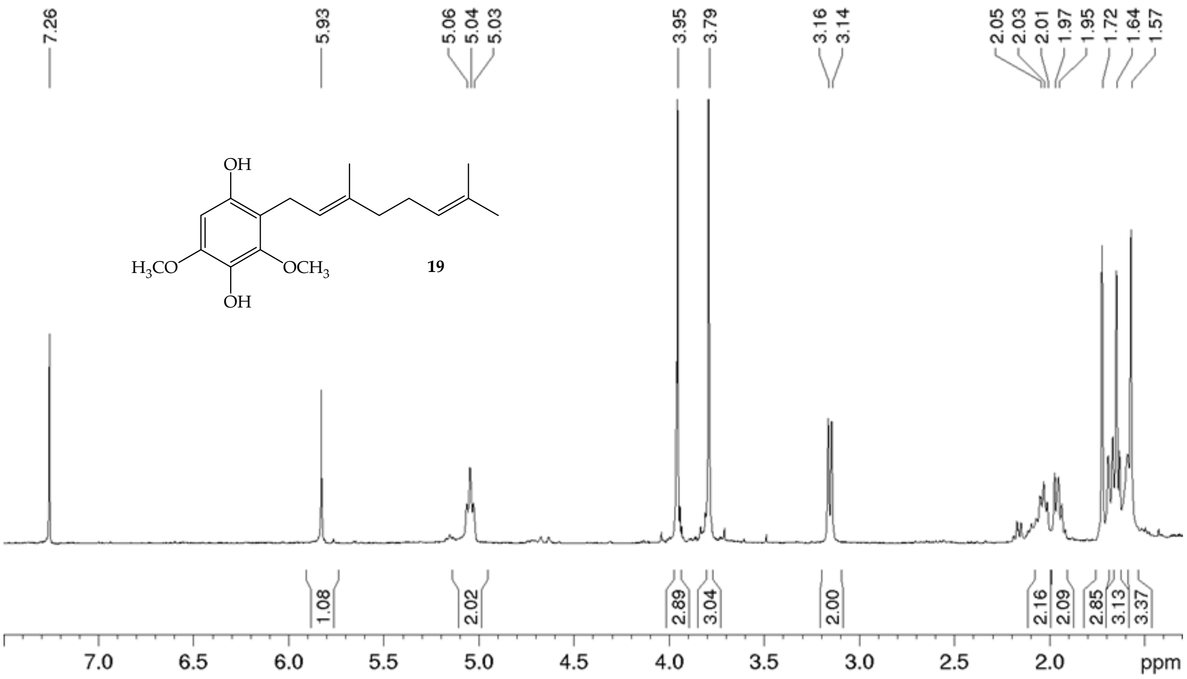

^{13}C-NMR

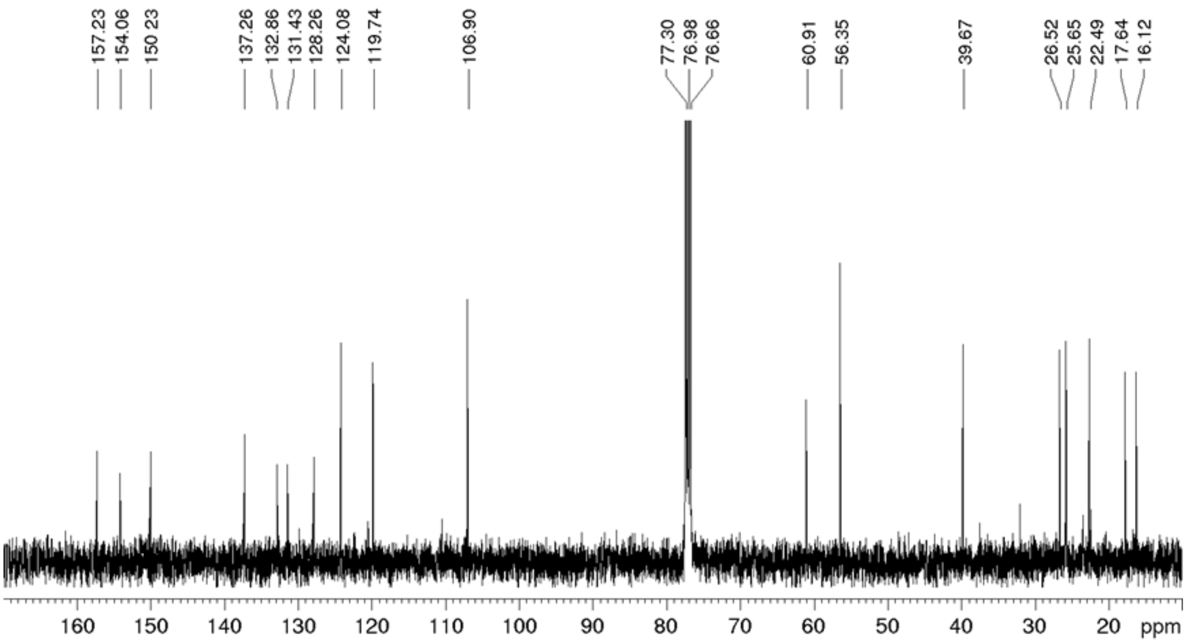

Figure S1: NMR spectra of (E)-2-(3,7-dimethylocta-2,6-dien-1-yl)-3,5-dimethoxybenzene-1,4-diol (**19**)

^{1}H-NMR

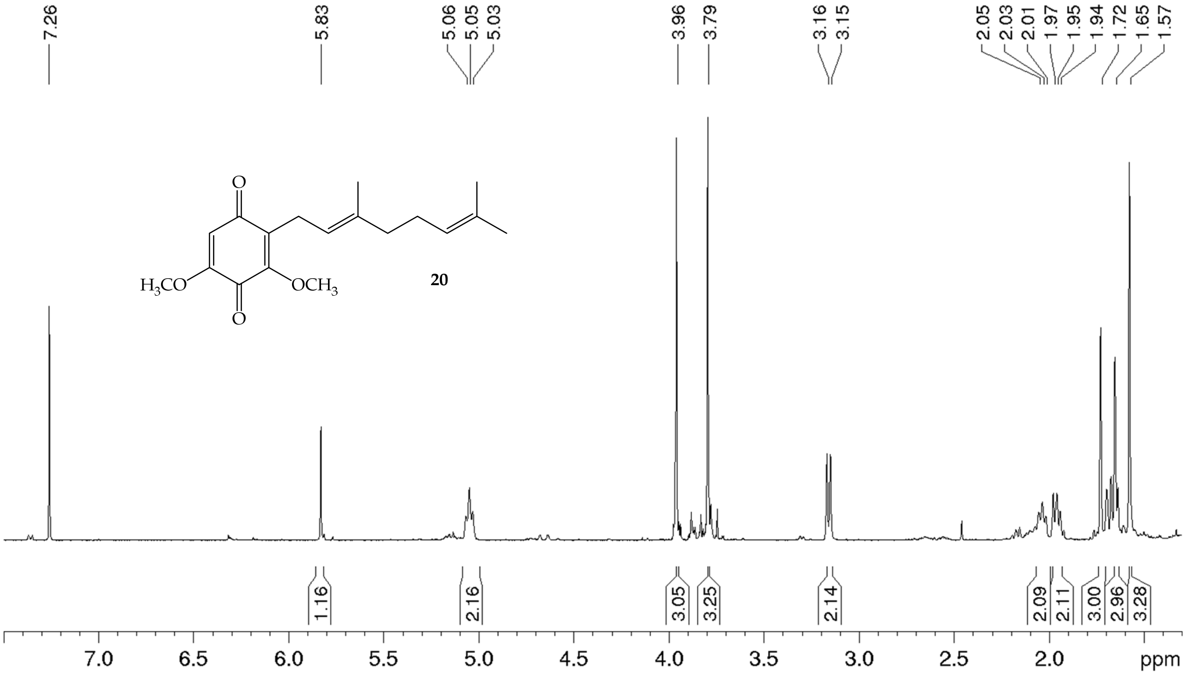

^{13}C-NMR

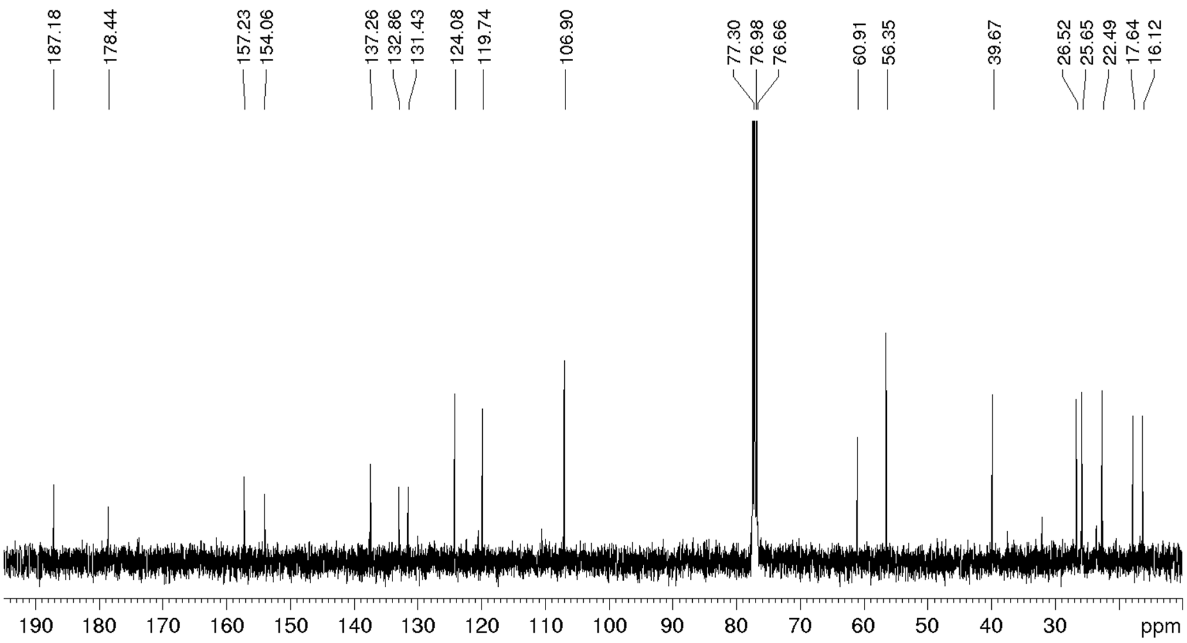

Figure S2: NMR spectra of (E)-2-(3,7-dimethylocta-2,6-dien-1-yl)-3,5-dimethoxycyclohexa-2,5-diene-1,4-dione (**20**)

^{1}H-NMR

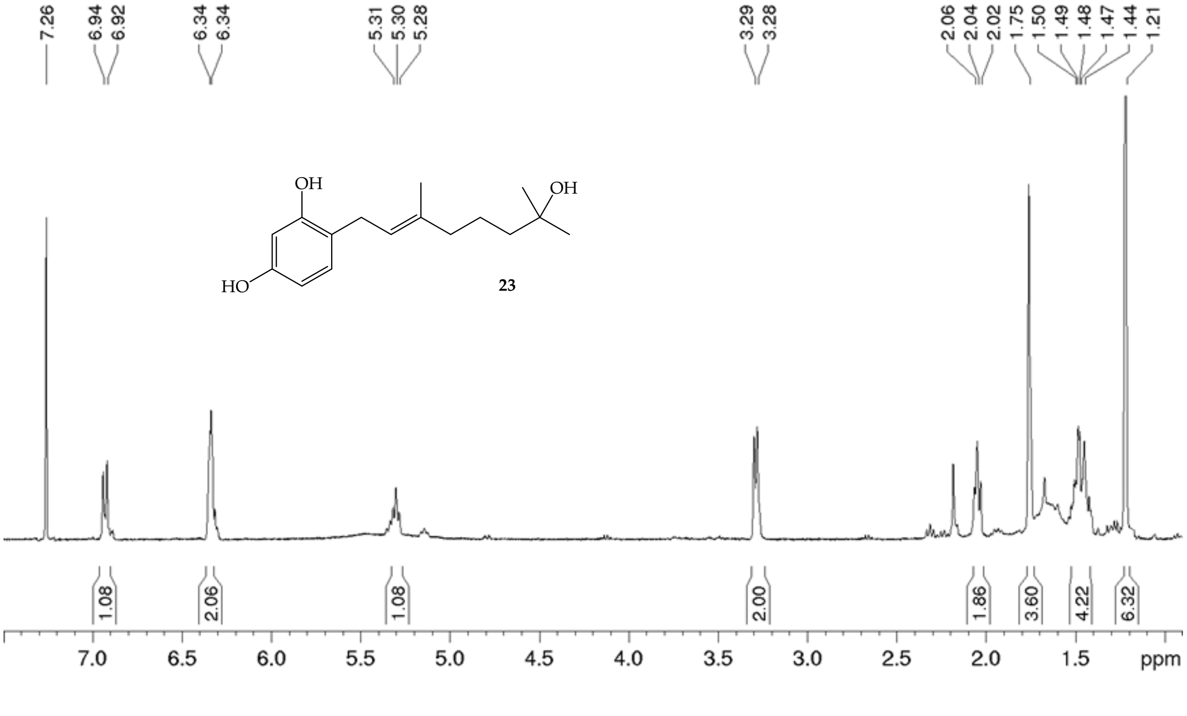

^{13}C-NMR

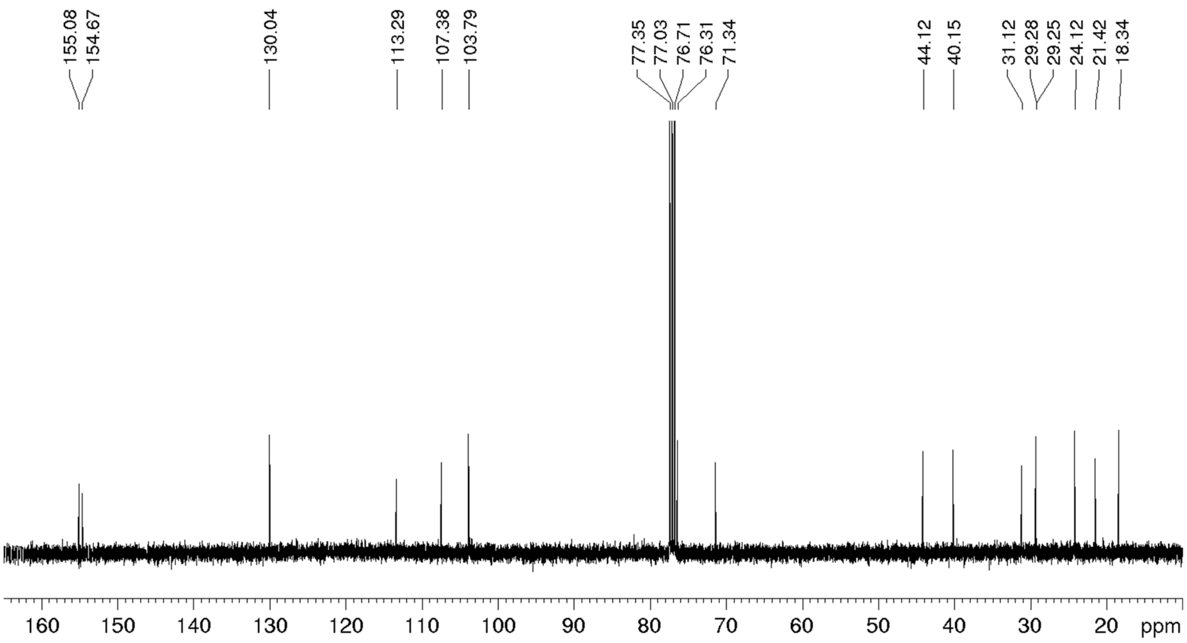

Figure S3: NMR spectra of (E)-4-(7-hydroxy-3,7-dimethyloct-2-en-1-yl)benzene-1,3-diol (**23**)

^{1}H-NMR

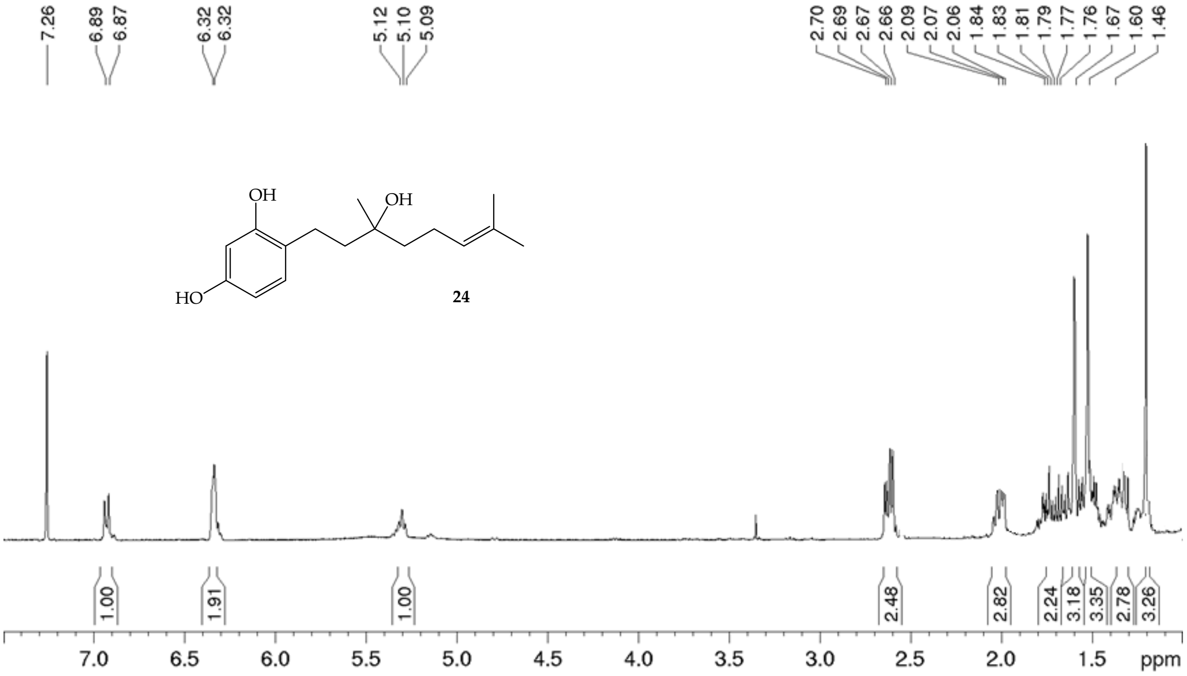

^{13}C-NMR

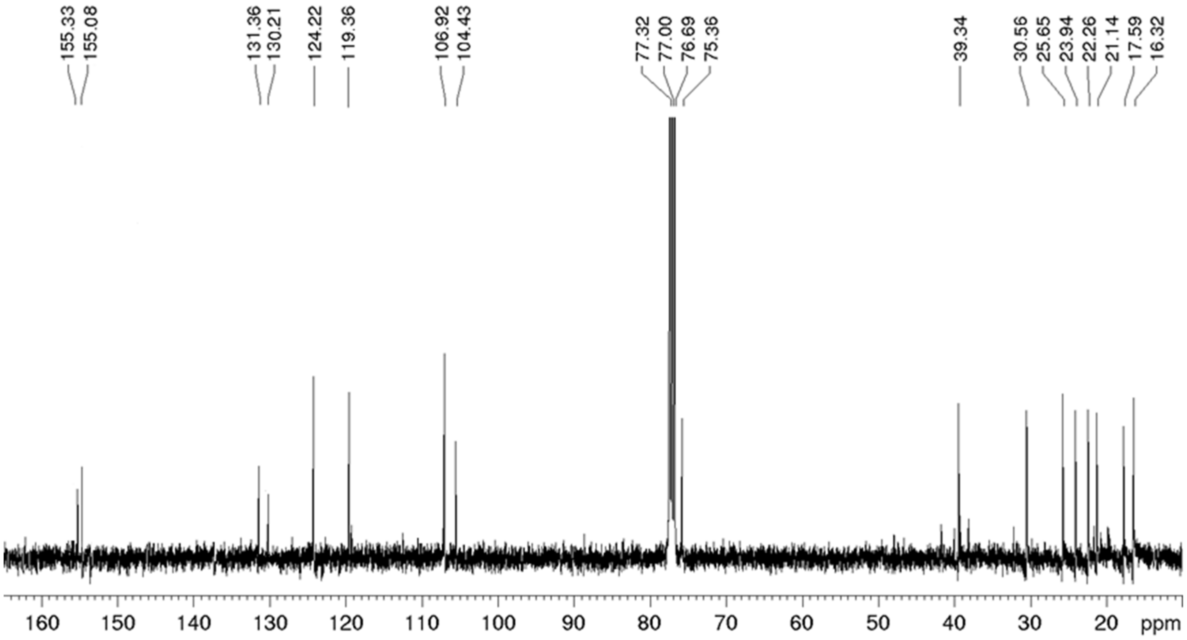

Figure S4: NMR spectra of 4-(3-hydroxy-3,7-dimethyloct-6-en-1-yl)benzene-1,3-diol (**24**)

^{1}H-NMR

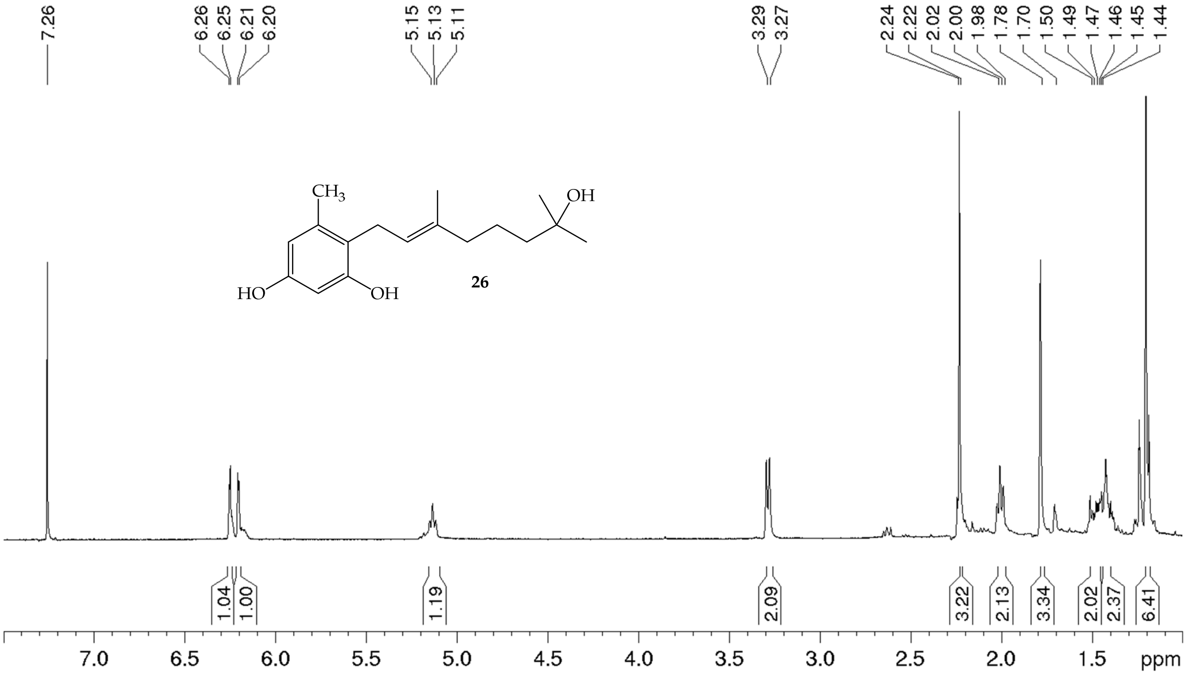

^{13}C-NMR

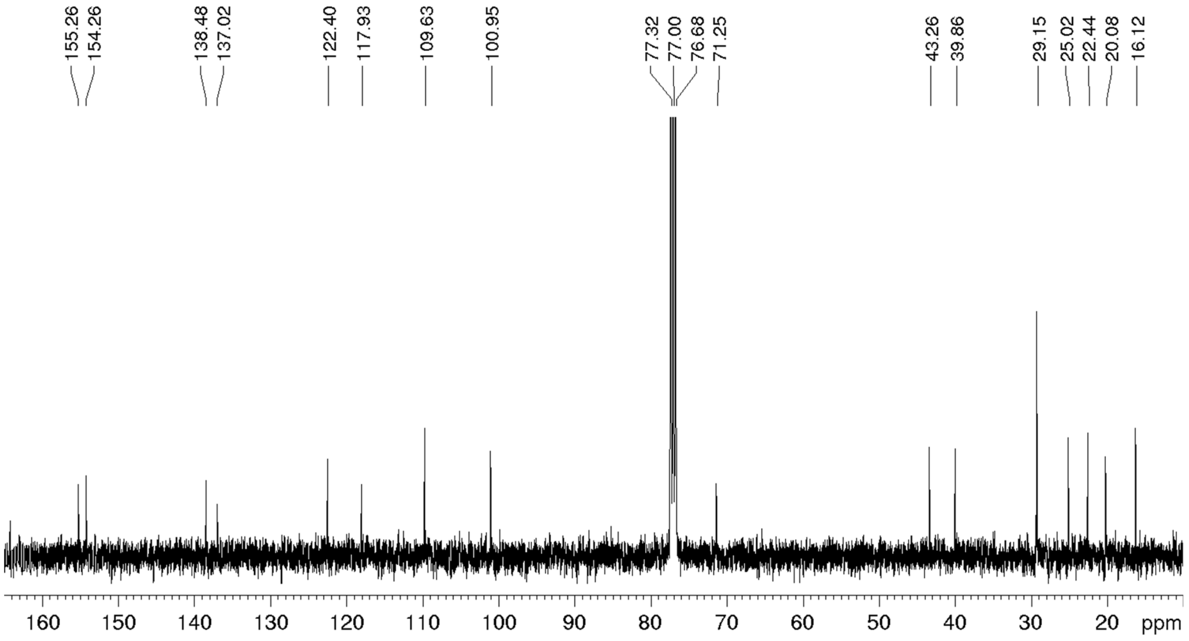

Figure S5: NMR spectra of (E)-4-(7-hydroxy-3,7-dimethyloct-2-en-1-yl)-5-methylbenzene-1,3-diol (**26**)

^{1}H-NMR

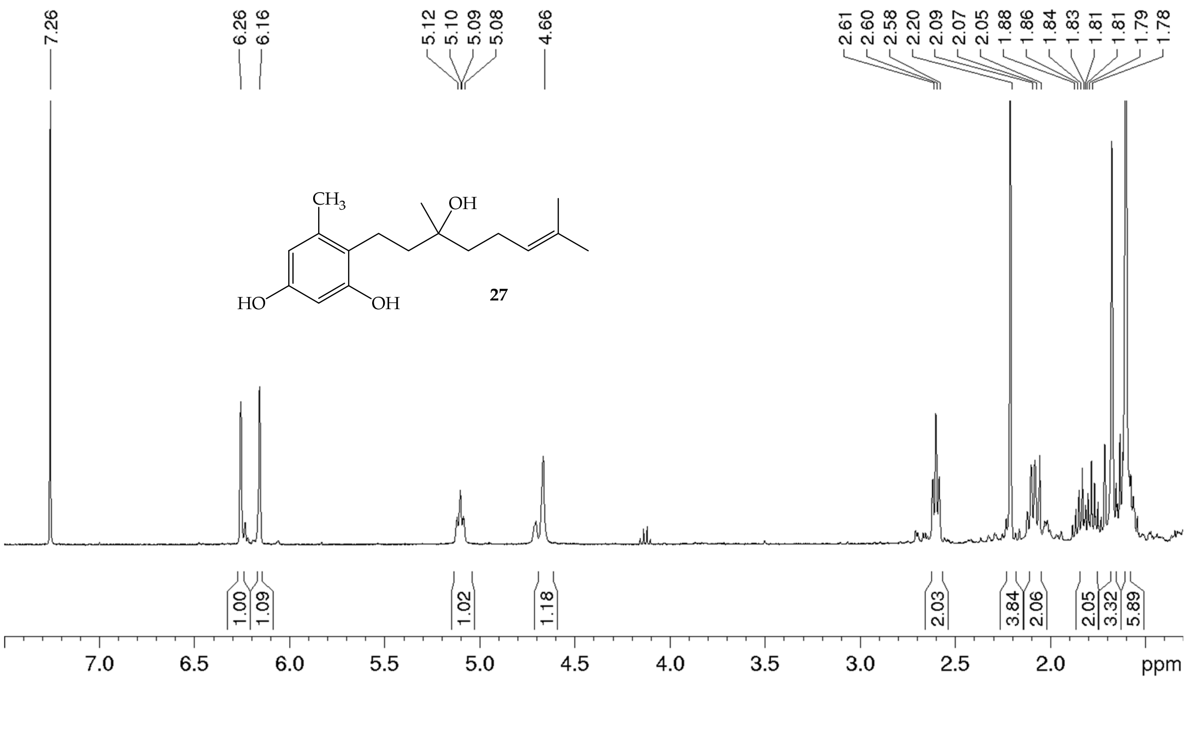

^{13}C-NMR

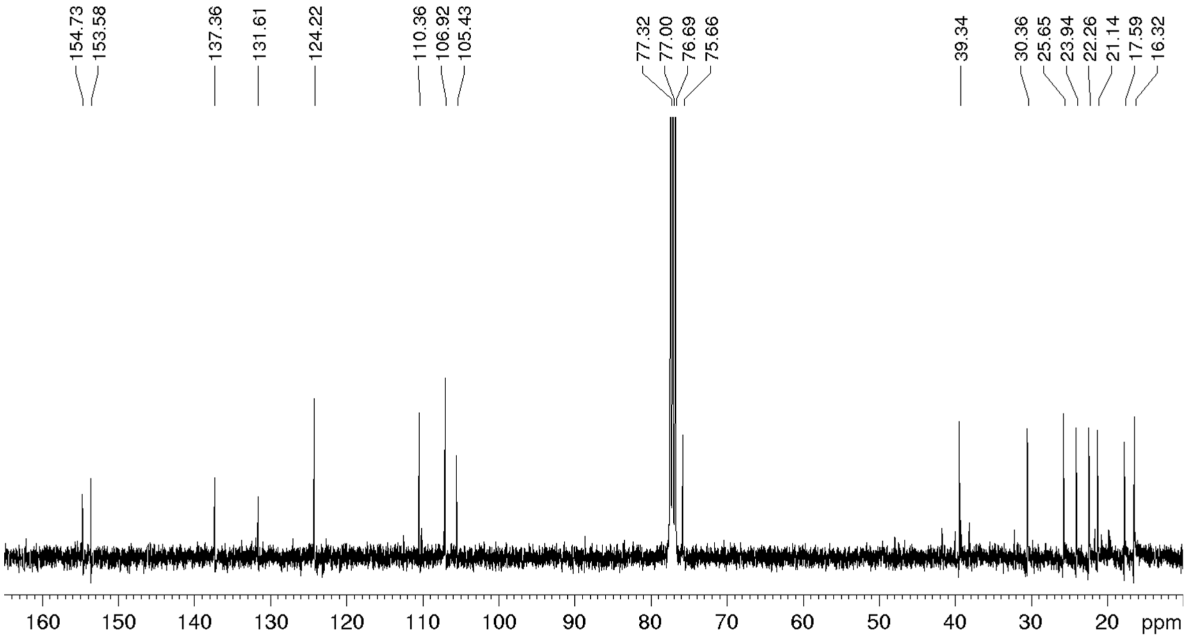

Figure S6: NMR spectra of 4-(3-hydroxy-3,7-dimethyloct-6-en-1-yl)-5-methylbenzene-1,3-diol (**27**)

^{1}H-NMR

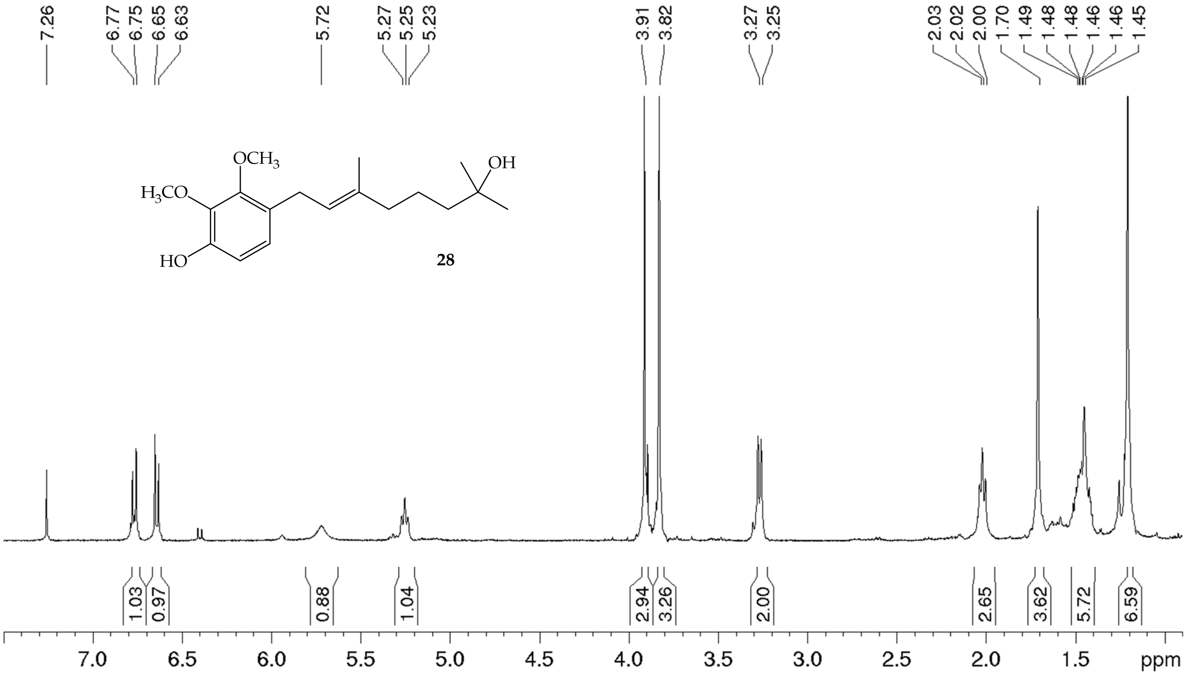

^{13}C-NMR

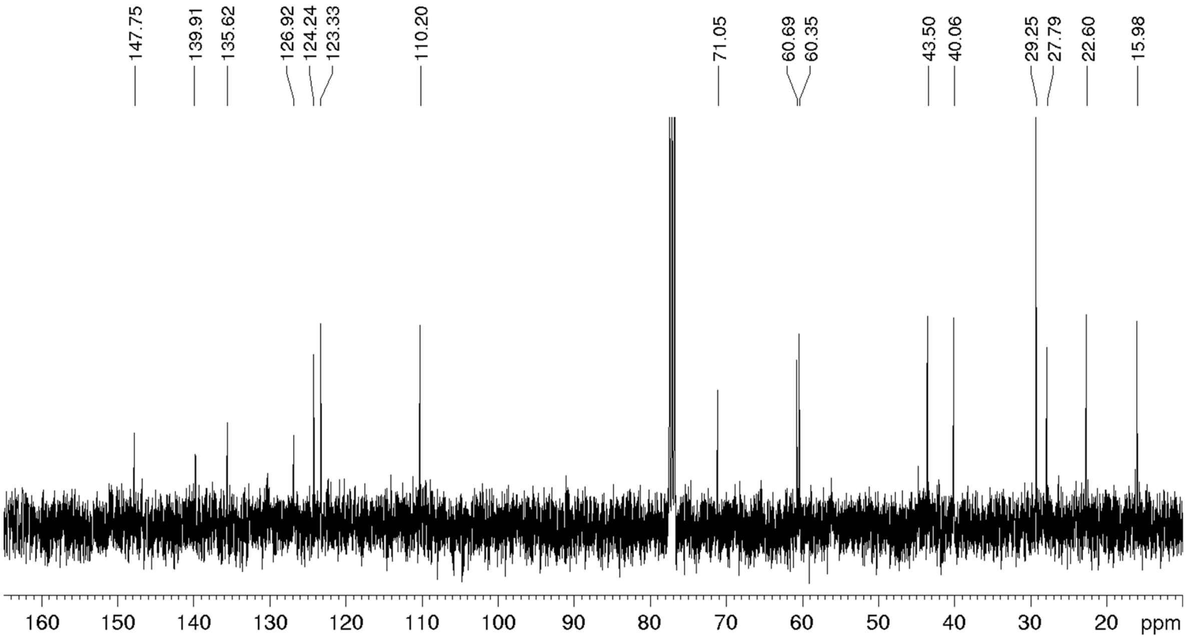

Figure S7: NMR spectra of (E)-4-(7-hydroxy-3,7-dimethyloct-2-en-1-yl)-2,3-dimethoxyphenol (**28**)

^{1}H-NMR

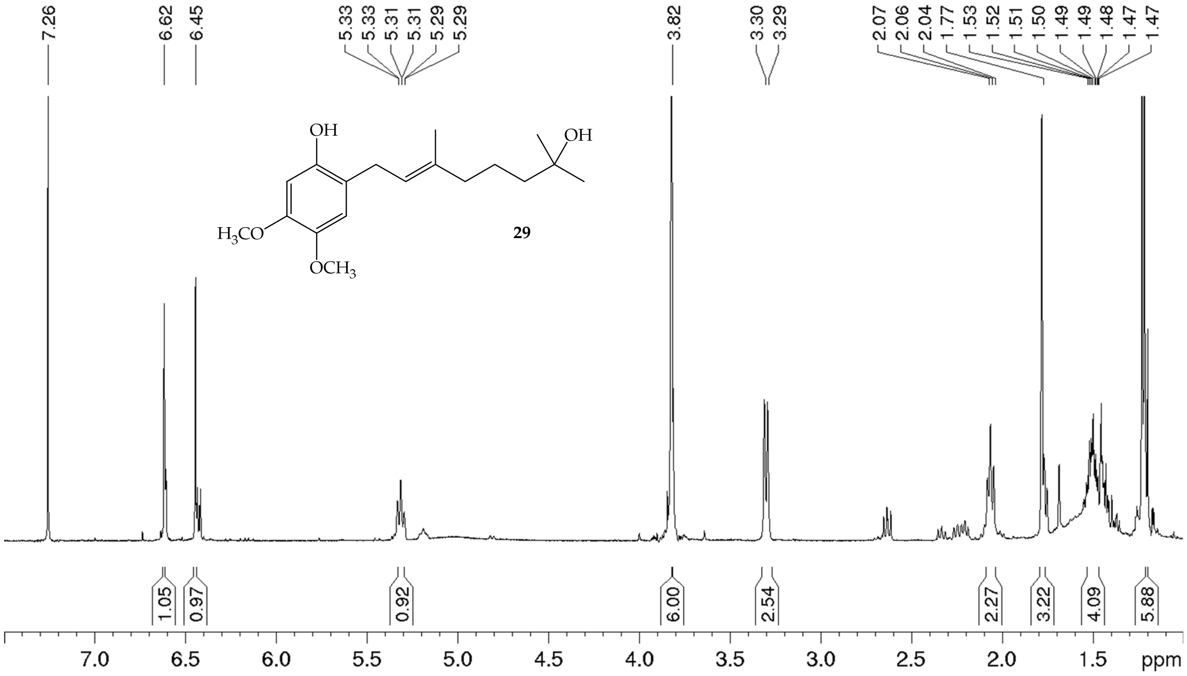

^{13}C-NMR

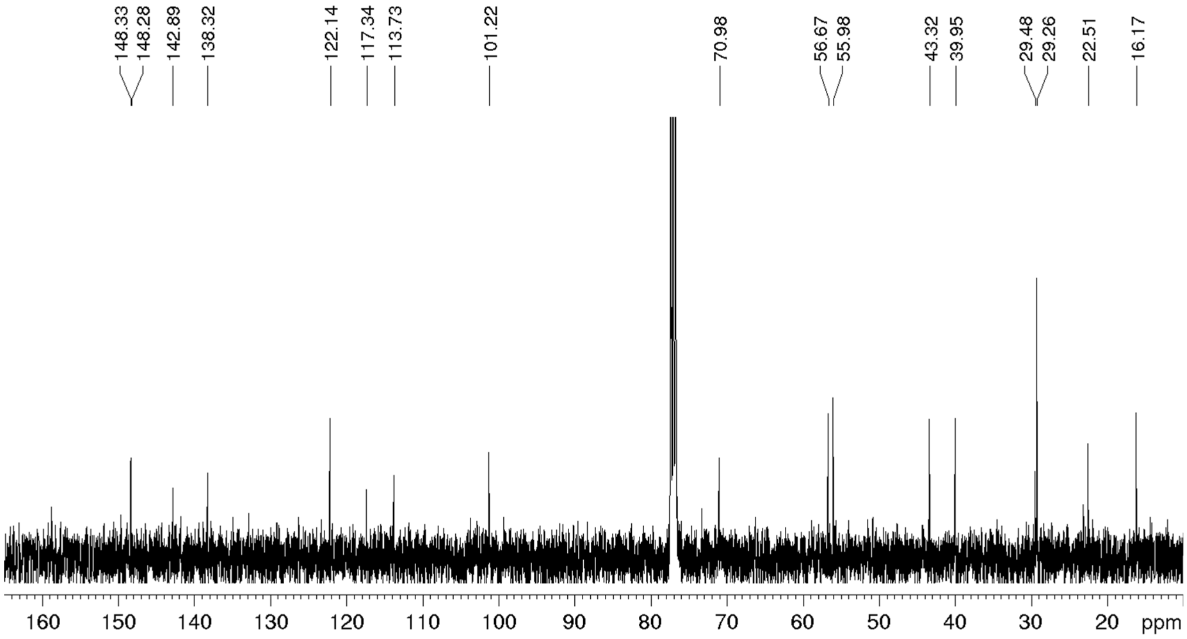

Figure S8: NMR spectra of (E)-2-(7-hydroxy-3,7-dimethyloct-2-en-1-yl)-4,5-dimethoxyphenol (**29**)

^{1}H-NMR

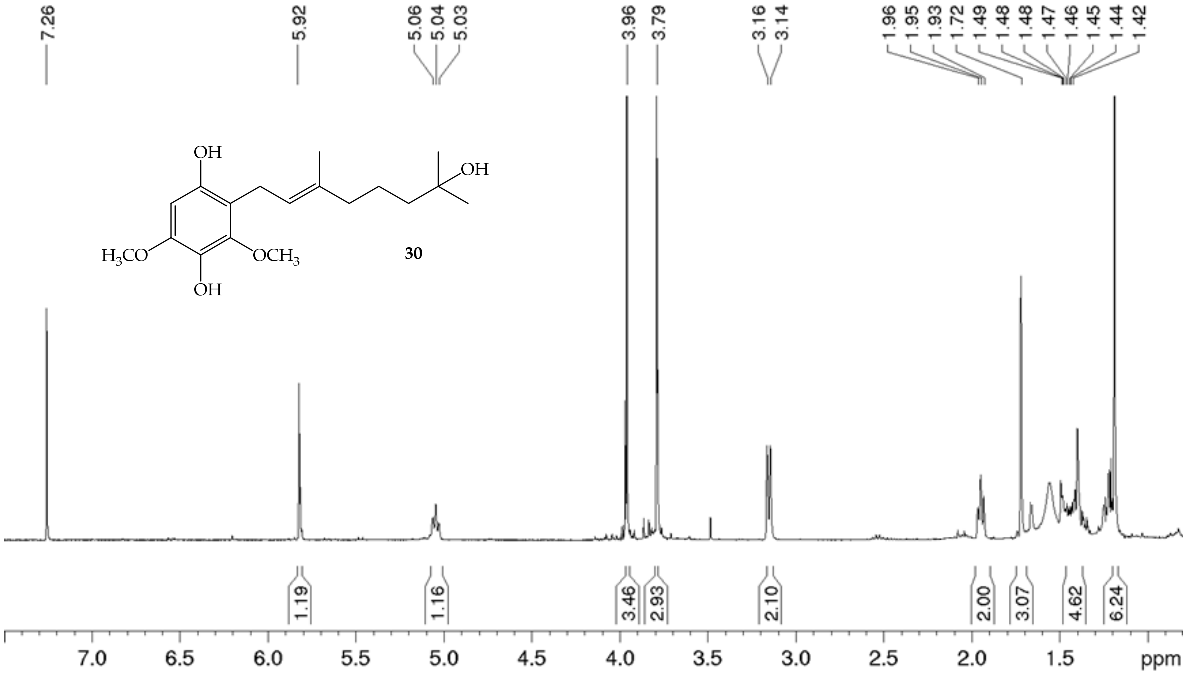

^{13}C-NMR

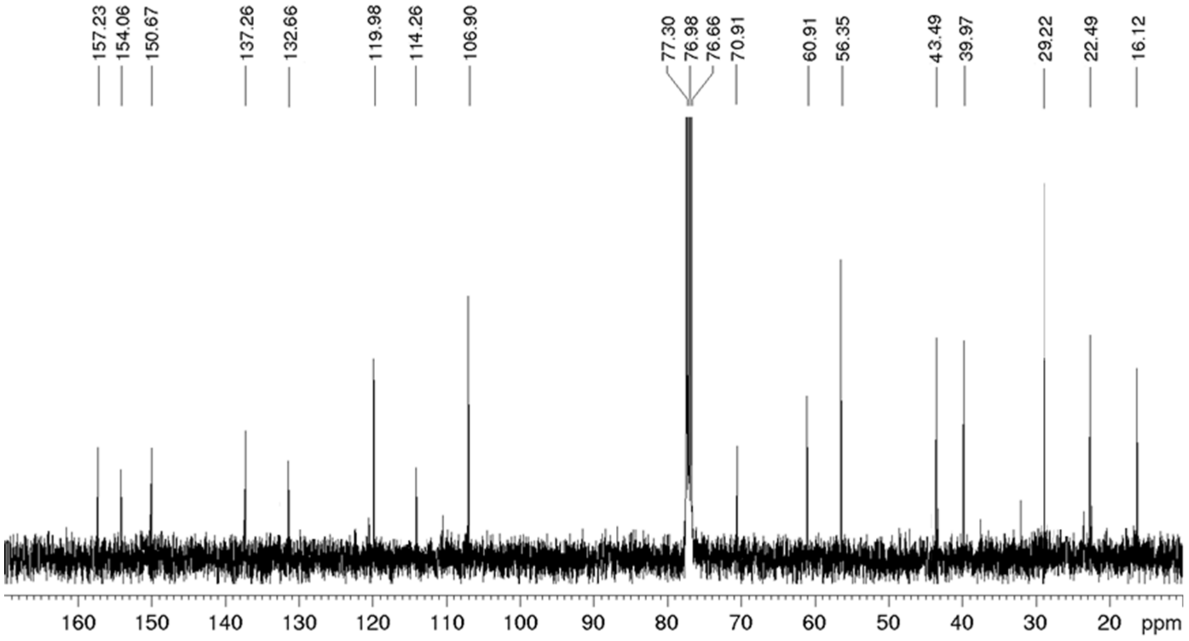

Figure S9: NMR spectra of (E)-2-(7-hydroxy-3,7-dimethyloct-2-en-1-yl)-3,5-dimethoxybenzene-1,4-diol (**30**)

^{1}H-NMR

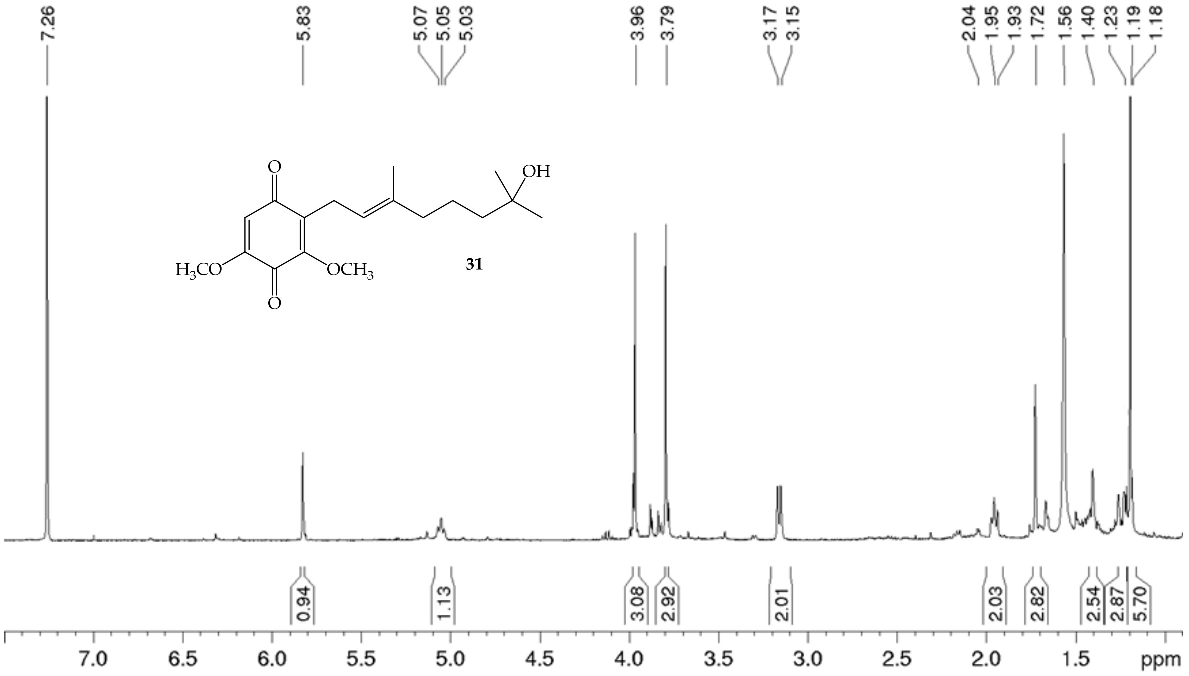

^{13}C-NMR

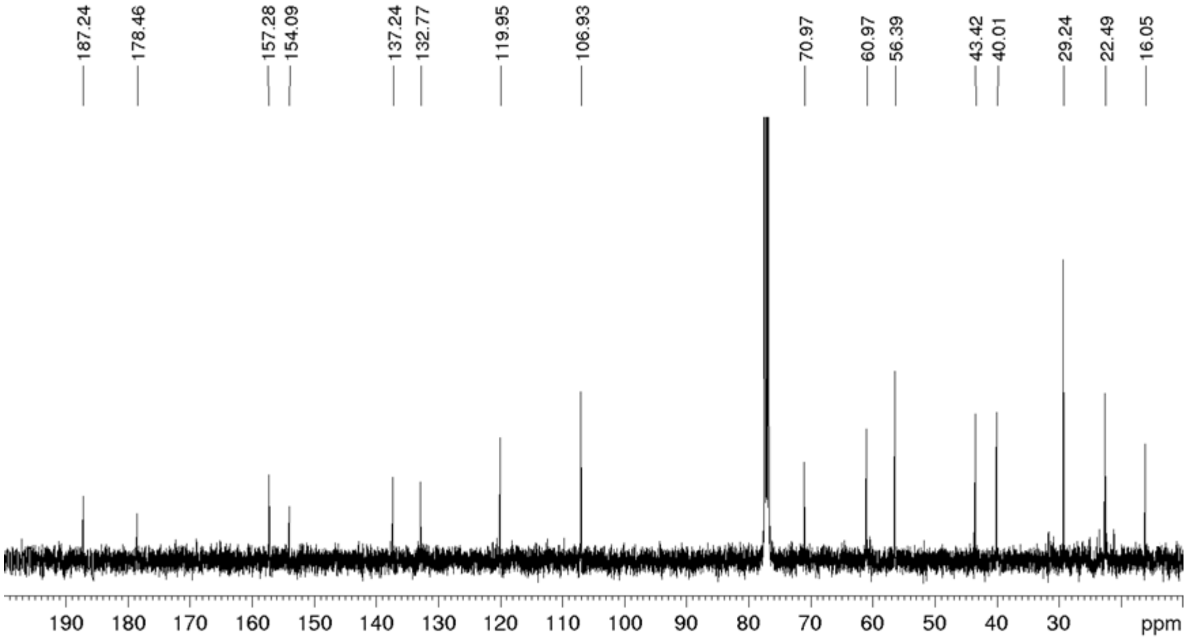

Figure S10: NMR spectra of (E)-2-(7-hydroxy-3,7-dimethyloct-2-en-1-yl)-3,5-dimethoxycyclohexa-2,5-diene-1,4-dione (**31**)

^{1}H-NMR

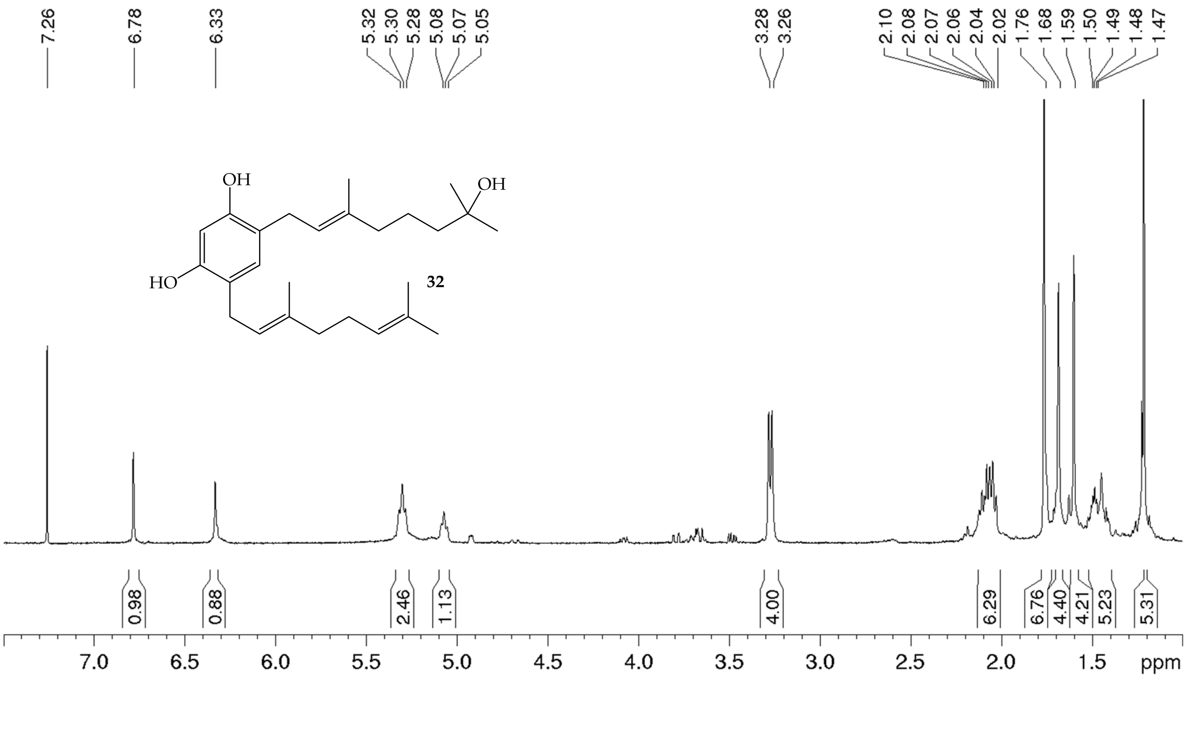

^{13}C-NMR

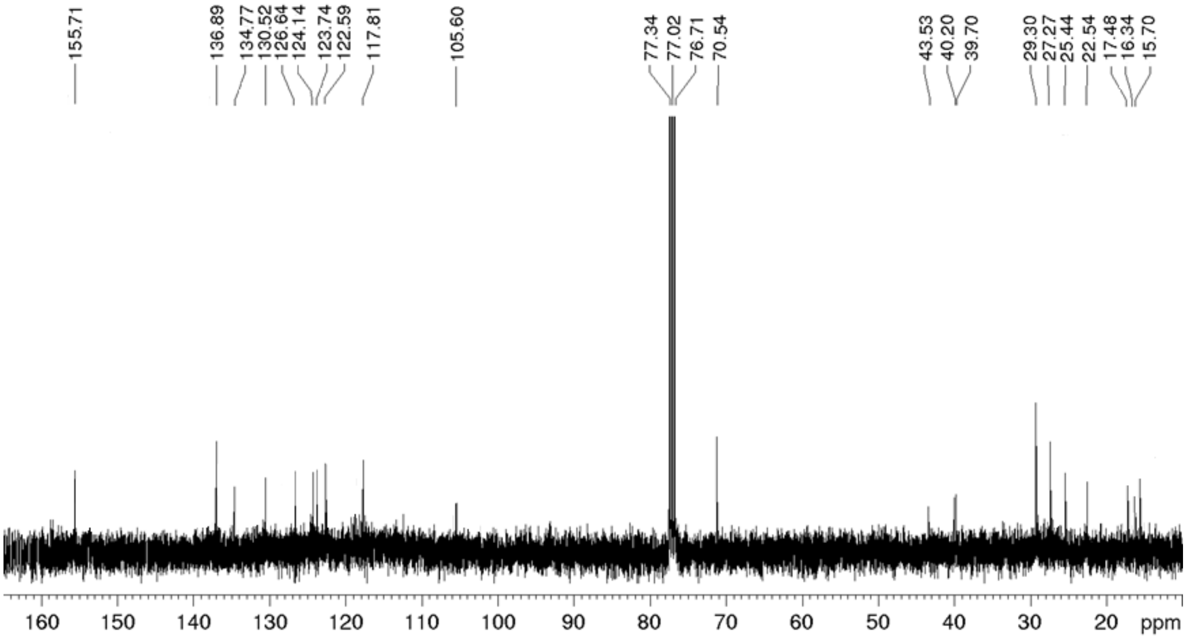

Figure S11: NMR spectra of 4-((E)-3,7-dimethylocta-2,6-dien-1-yl)-6-((E)-7-hydroxy-3,7-dimethyloct-2-en-1-yl)benzene-1,3-diol (**32**)

^{1}H-NMR

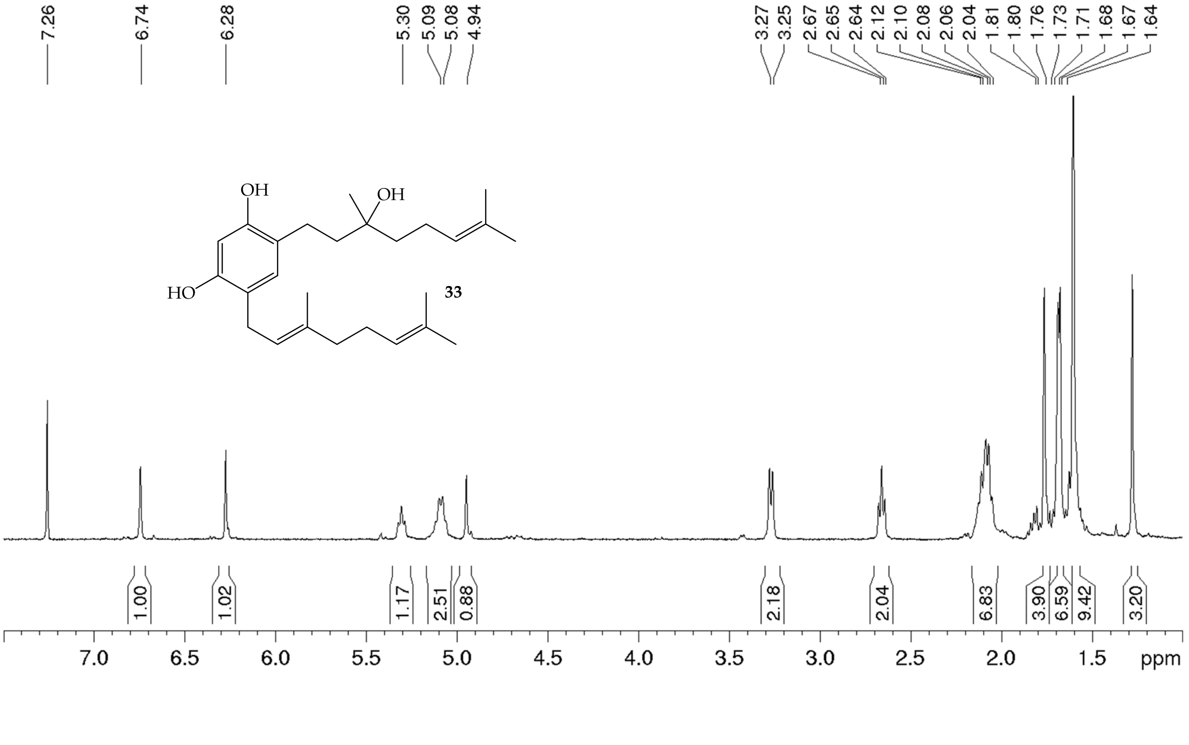

^{13}C-NMR

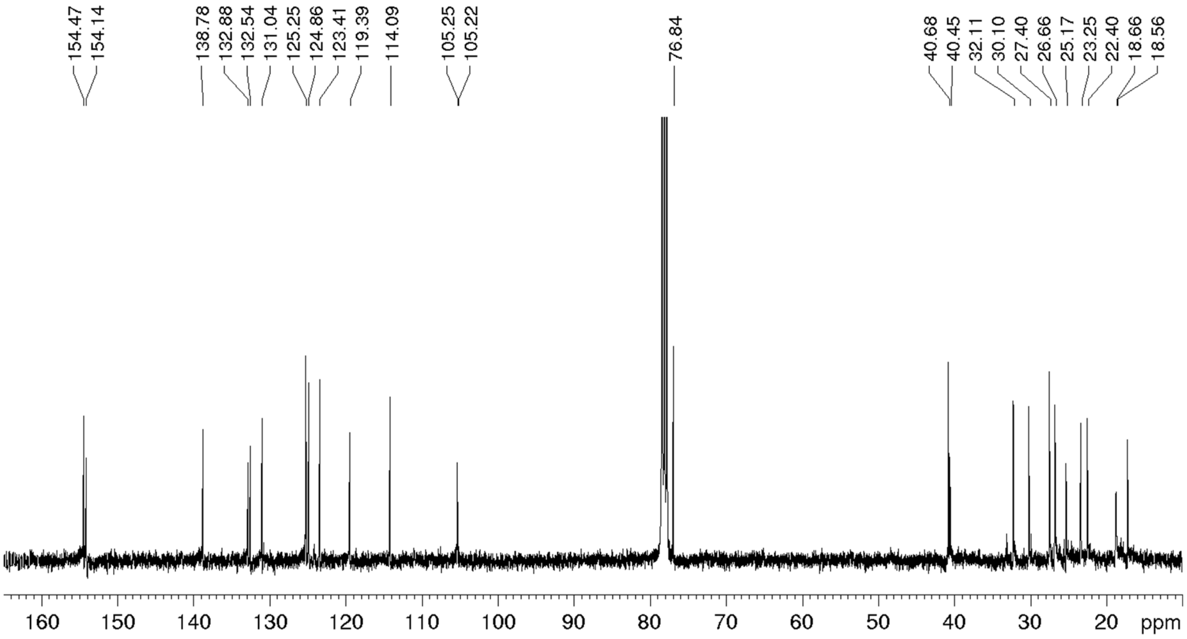

Figure S12: NMR spectra of (E)-4-(3,7-dimethylocta-2,6-dien-1-yl)-6-(3-hydroxy-3,7-dimethyloct-6-en-1-yl)benzene-1,3-diol (**33**)

^{1}H-NMR

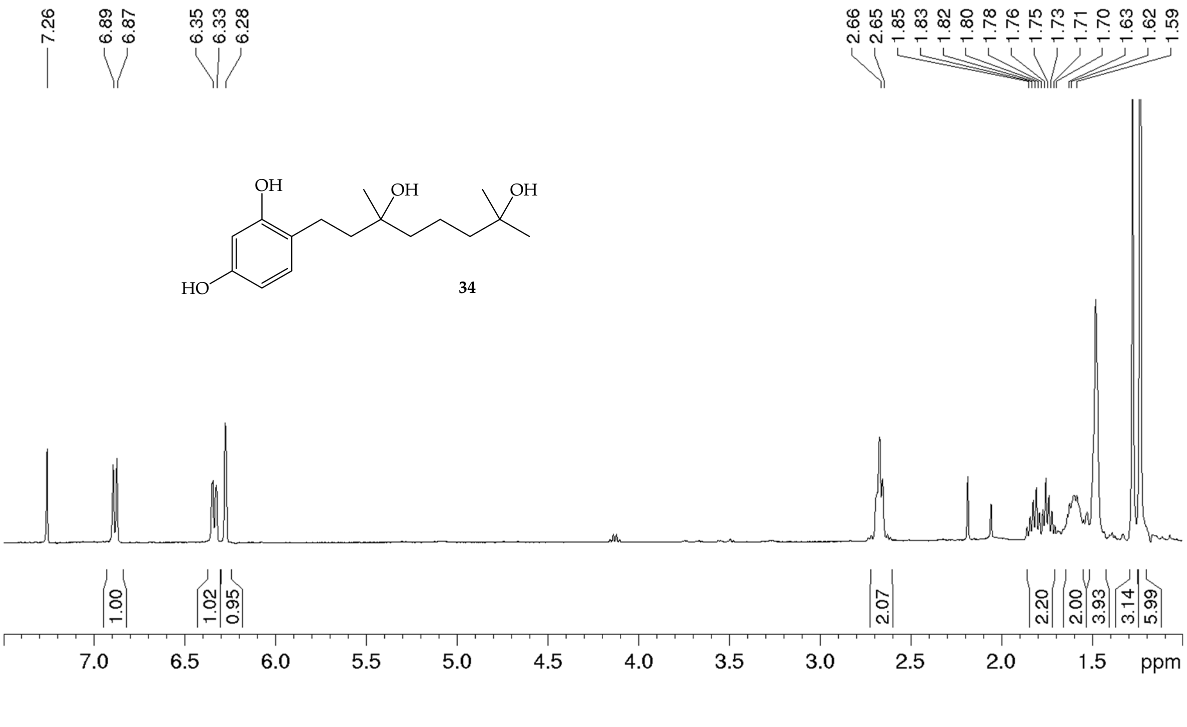

^{13}C-NMR

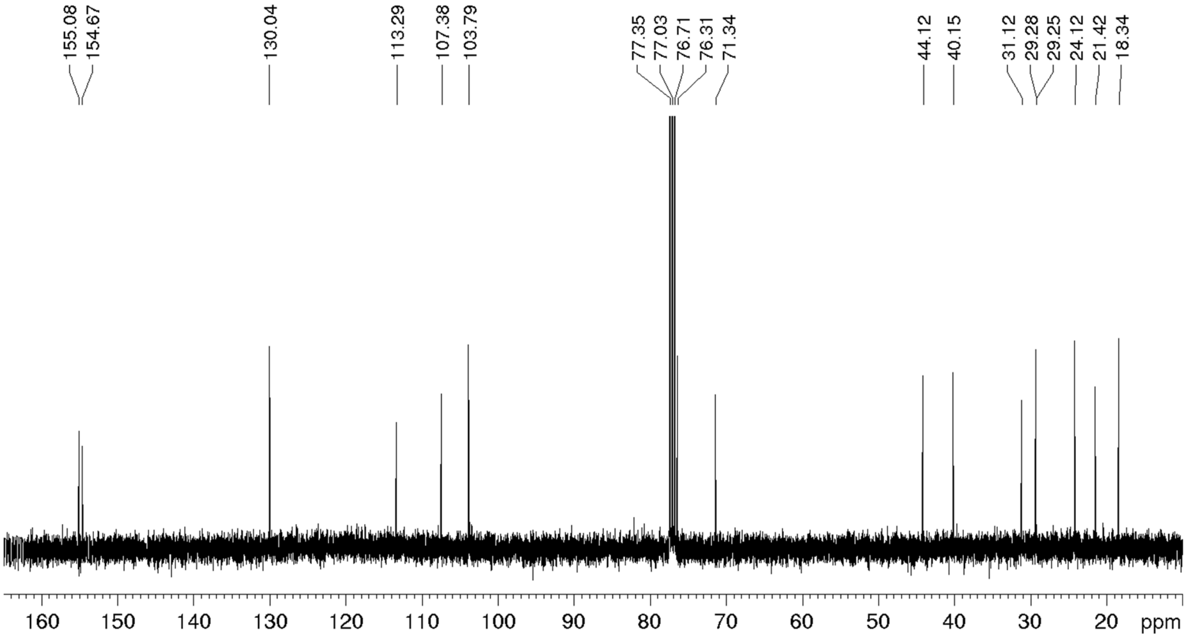

Figure S13: NMR spectra of (E)-4-(7-hydroxy-3,7-dimethyloct-2-en-1-yl)benzene-1,3-diol (23); and 4-(3,7-dihydroxy-3,7-dimethyloctyl)benzene-1,3-diol (**34**)

^{1}H-NMR

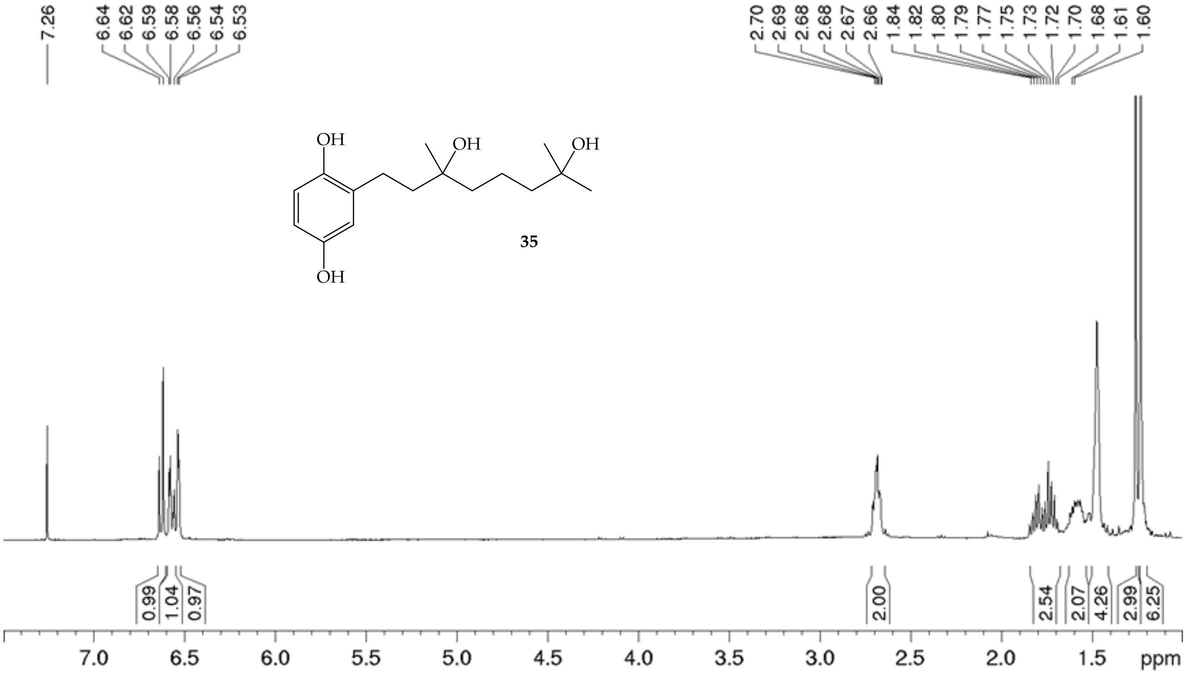

^{13}C-NMR

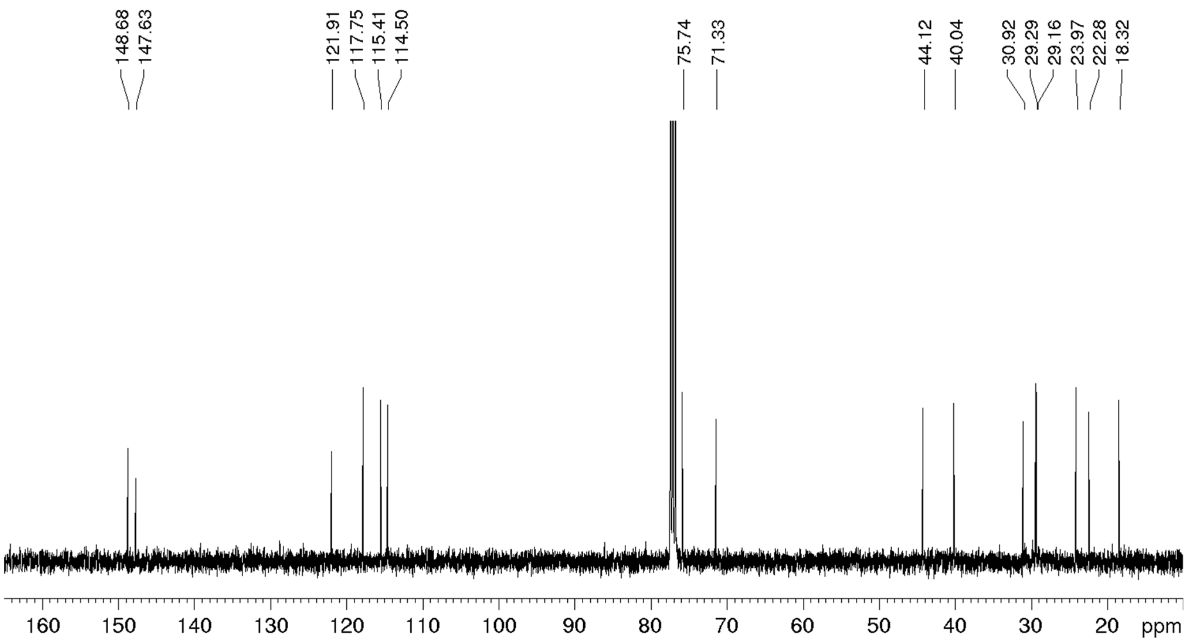

Figure S14: NMR spectra of 2-(3,7-dihydroxy-3,7-dimethyloctyl)benzene-1,4-diol (**35**)

^{1}H-NMR

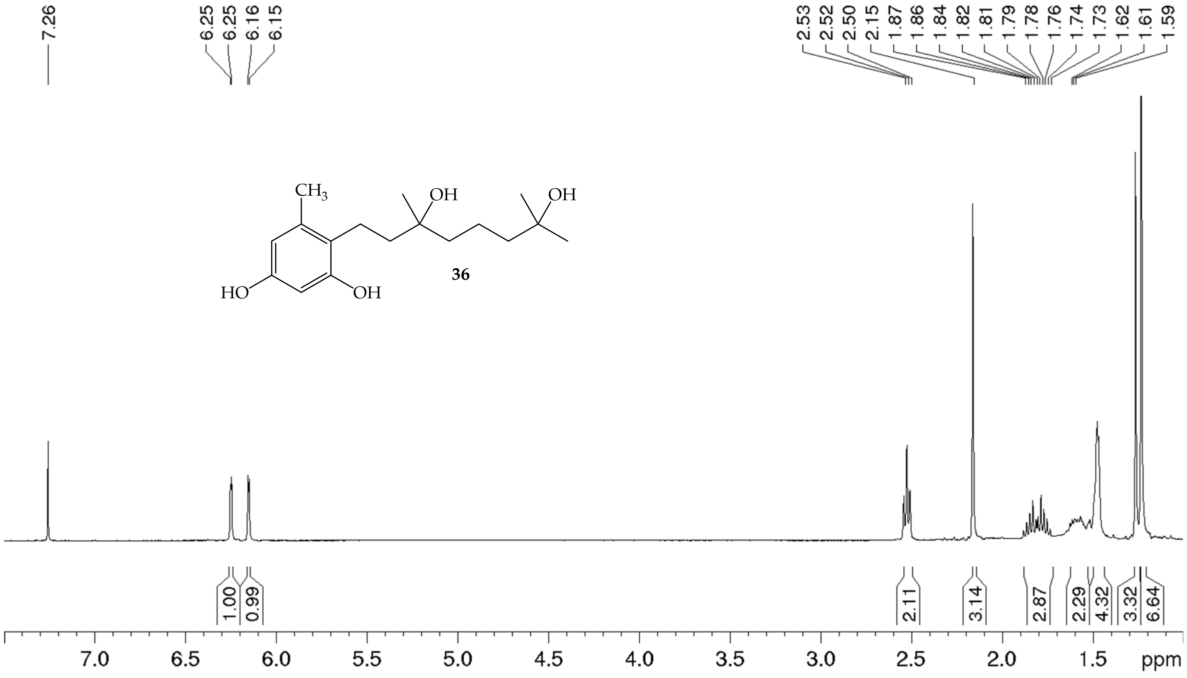

^{13}C-NMR

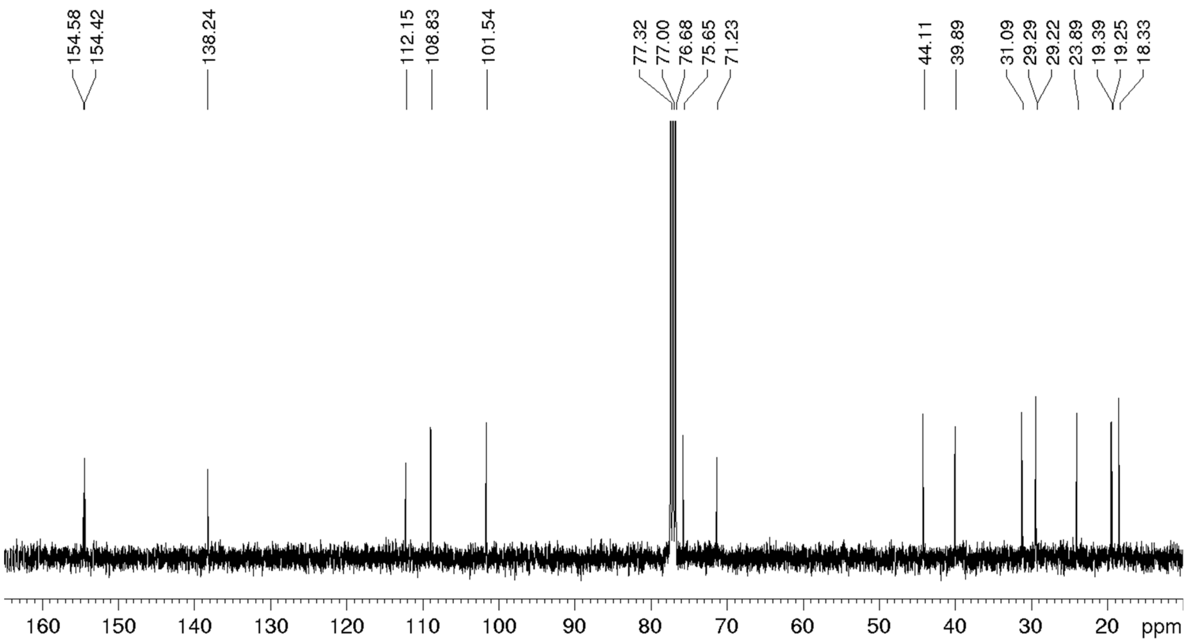

Figure S15: NMR spectra of 4-(3,7-dihydroxy-3,7-dimethyloctyl)-5-methylbenzene-1,3-diol (**36**)

^{1}H-NMR

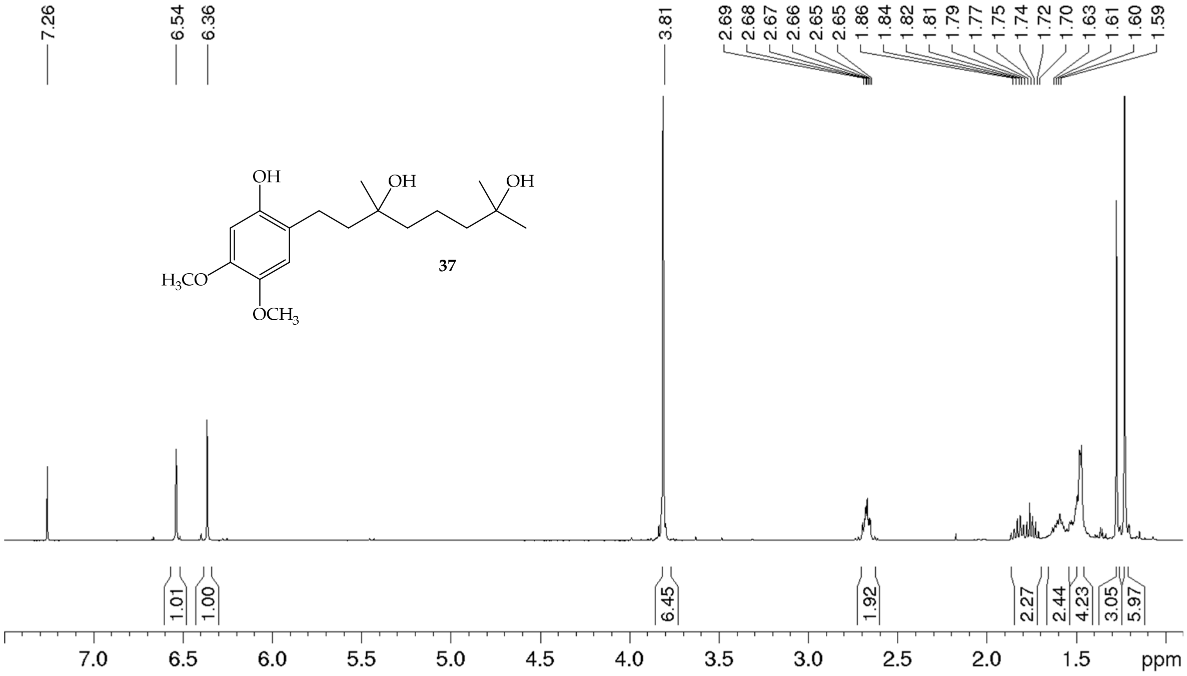

^{13}C-NMR

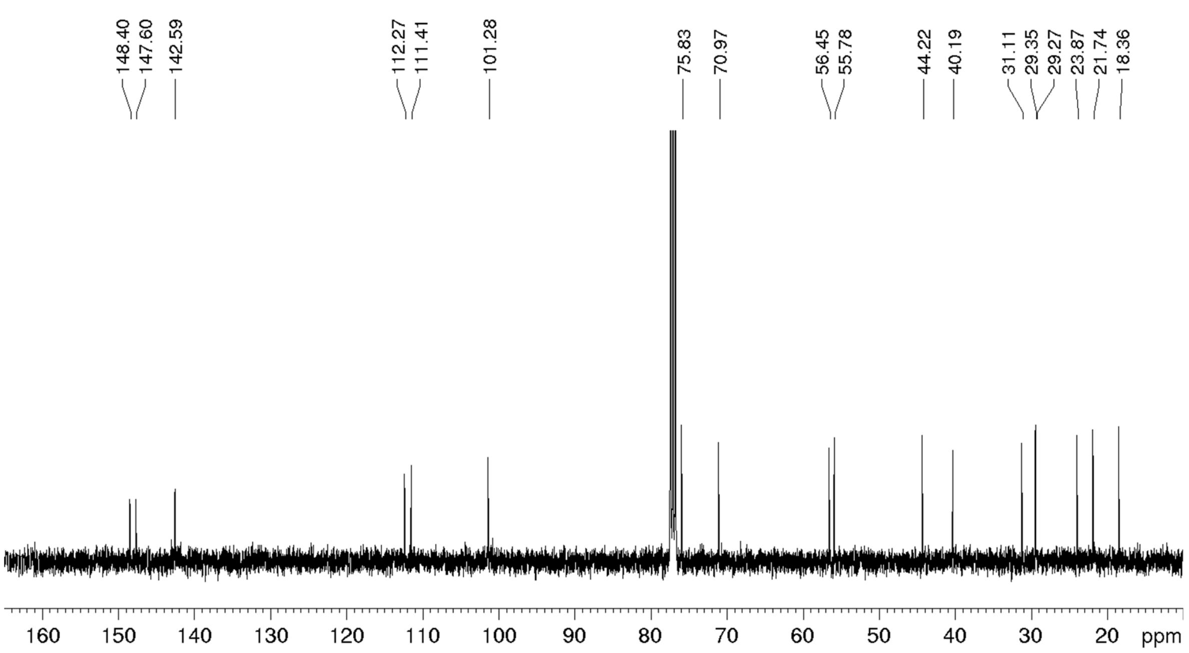

Figure S16: NMR spectra of 8-(2-hydroxy-4,5-dimethoxyphenyl)-2,6-dimethyloctane-2,6-diol (**37**)

¹H-NMR

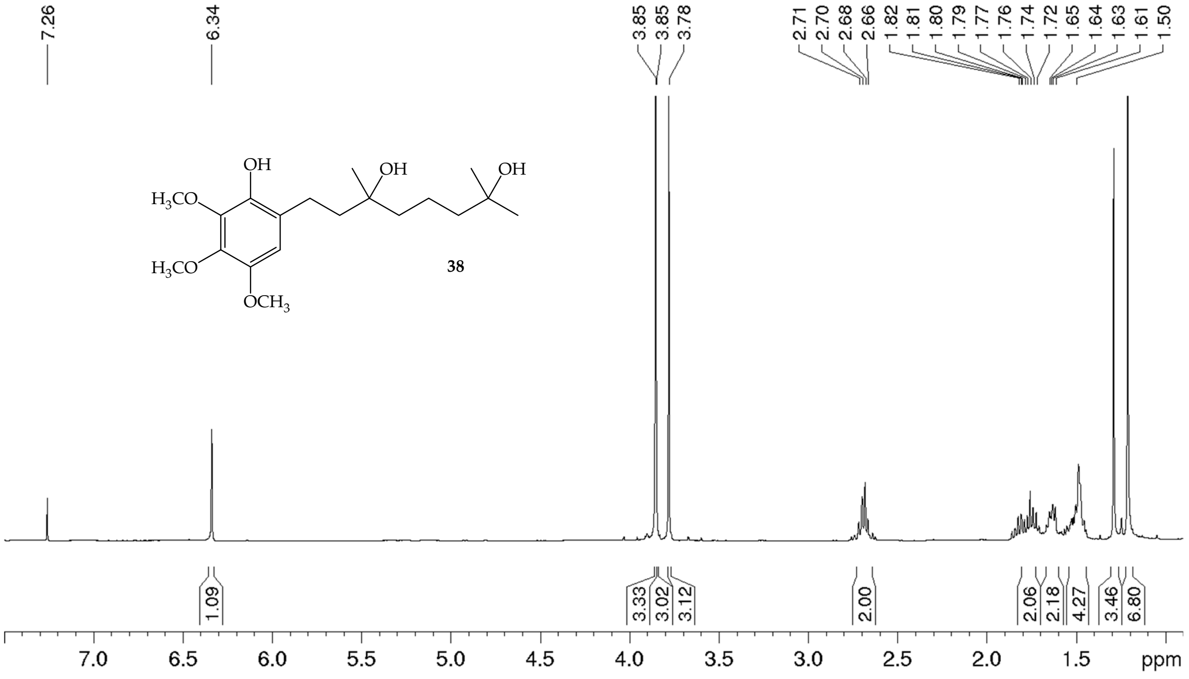

¹³C-NMR

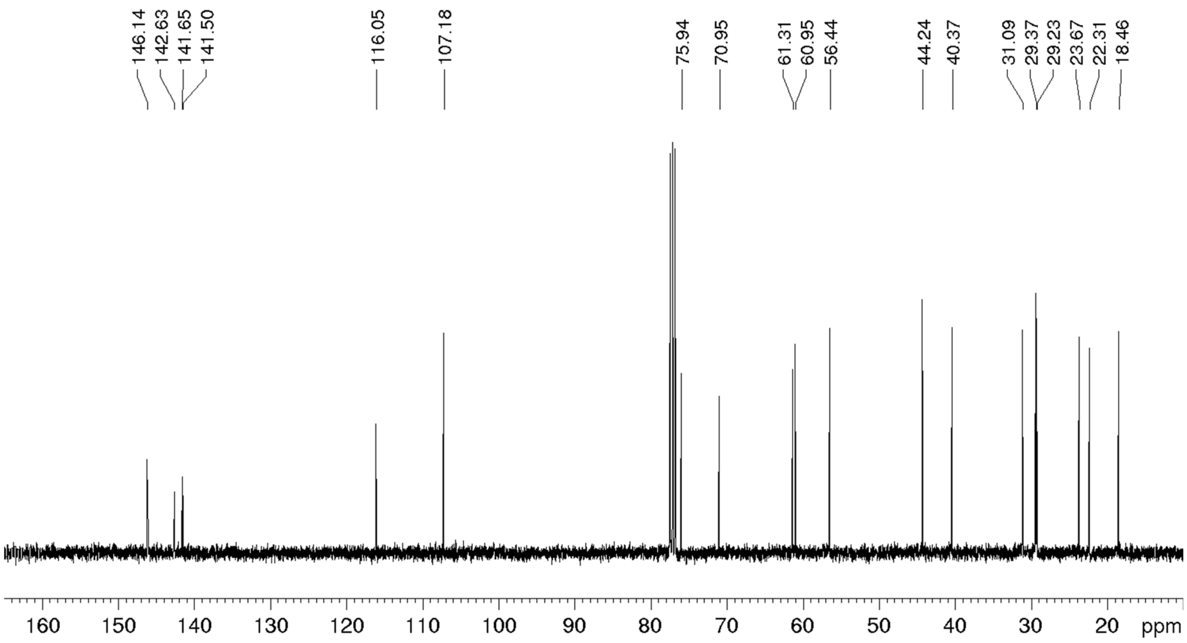

Figure S17: NMR spectra of 8-(2-hydroxy-3,4,5-trimethoxyphenyl)-2,6-dimethyloctane-2,6-diol (**38**)

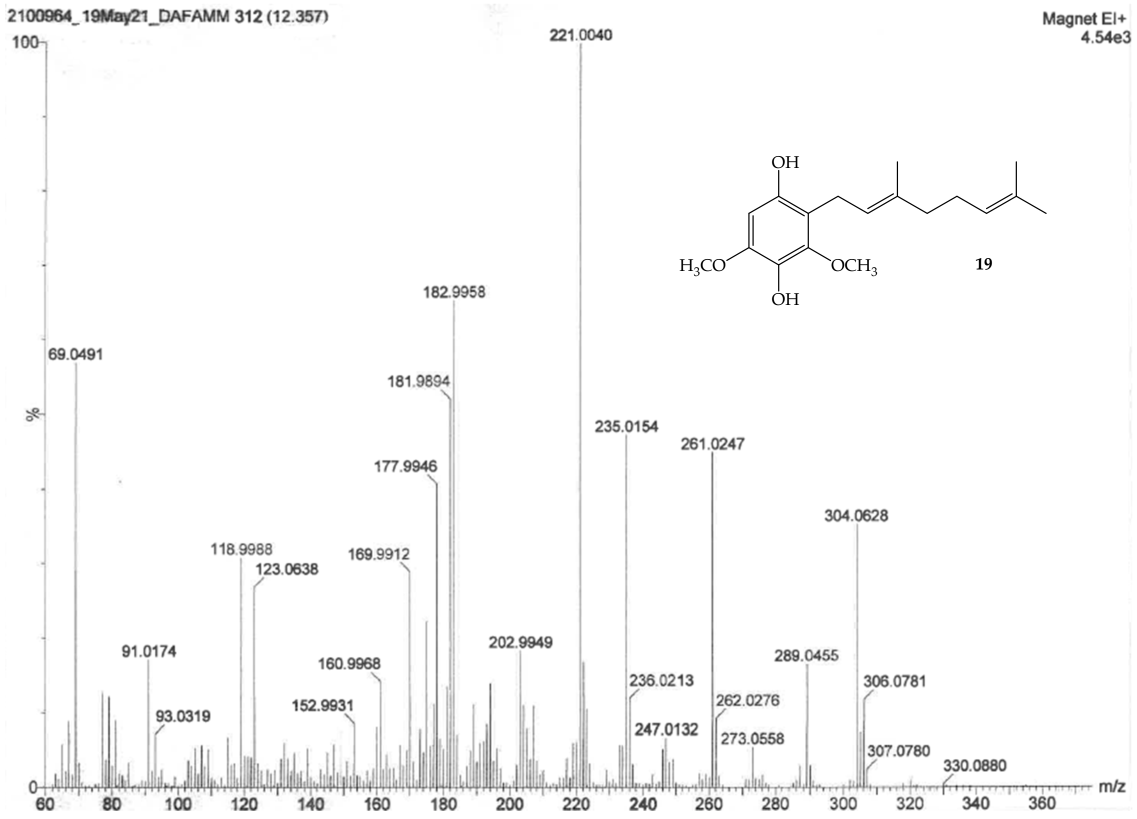

Figure S18: HRMS (EI$^+$) spectrum of (E)-2-(3,7-dimethylocta-2,6-dien-1-yl)-3,5-dimethoxybenzene-1,4-diol (**19**)

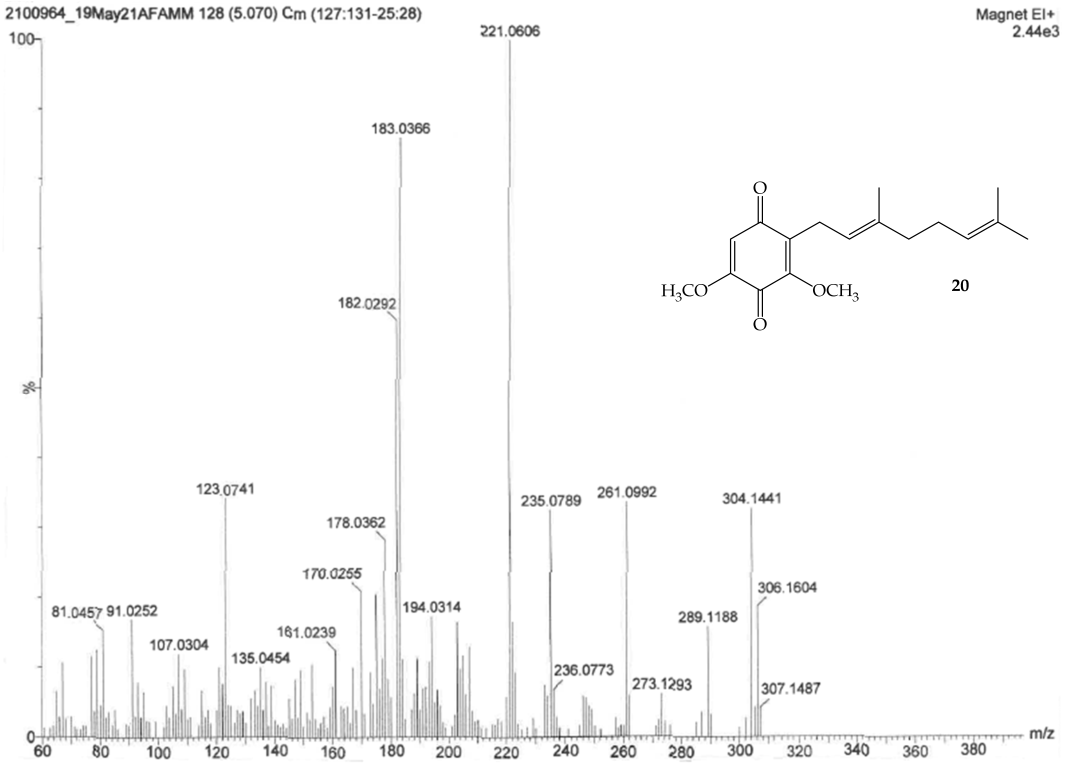

Figure S19: HRMS (EI+) spectrum of (E)-2-(3,7-dimethylocta-2,6-dien-1-yl)-3,5-dimethoxycyclohexa-2,5-diene-1,4-dione (**20**)

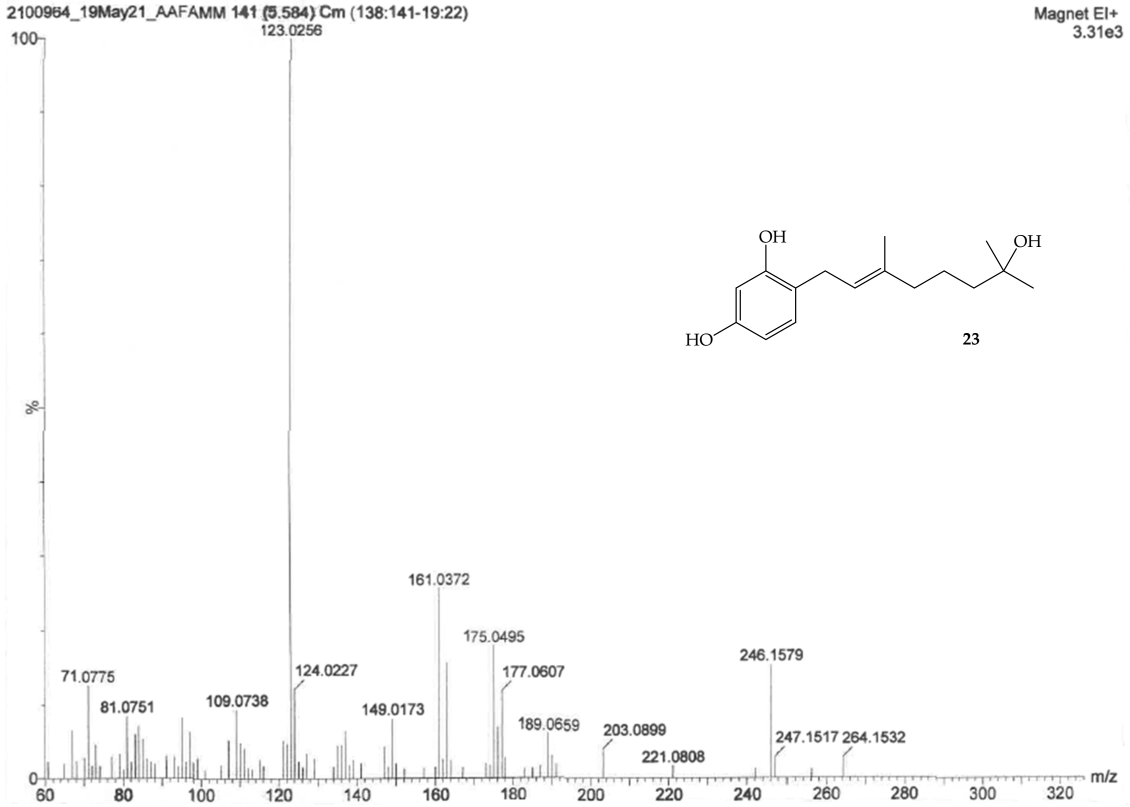

Figure S20: HRMS (EI+) spectrum of (E)-4-(7-hydroxy-3,7-dimethyloct-2-en-1-yl)benzene-1,3-diol (**23**)

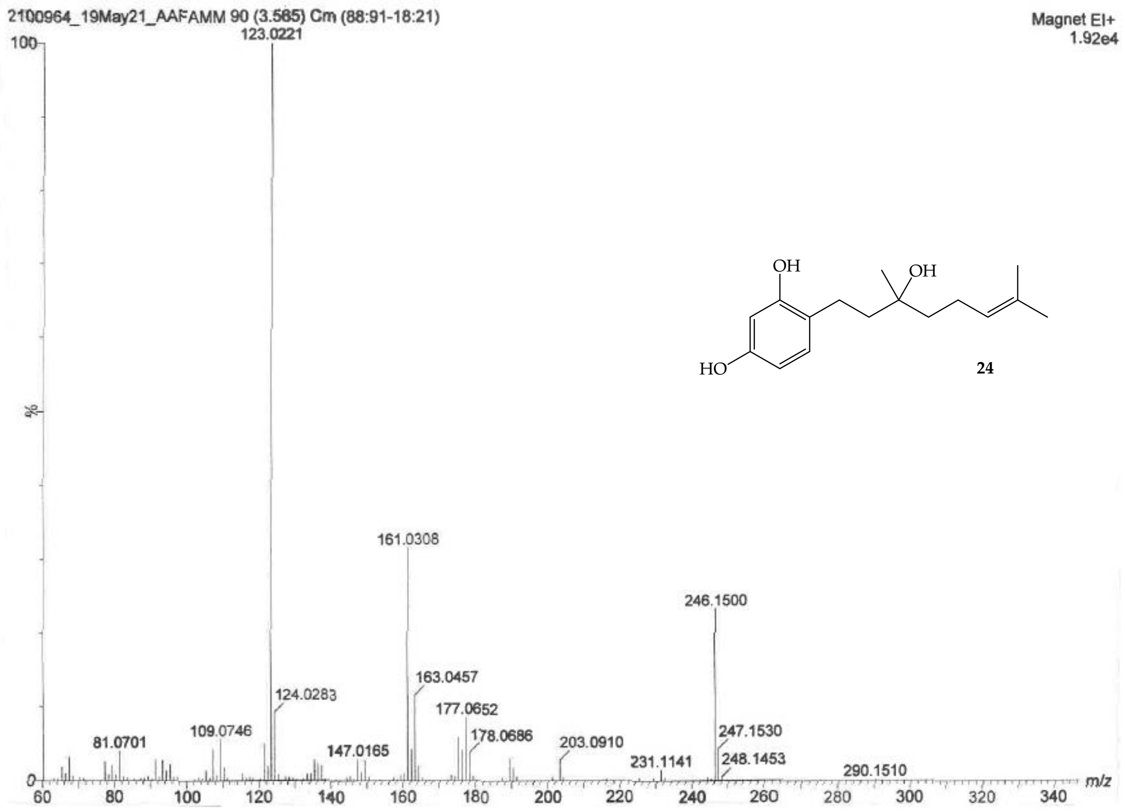

Figure S21: HRMS (EI+) spectrum of 4-(3-hydroxy-3,7-dimethyloct-6-en-1-yl)benzene-1,3-diol (**24**)

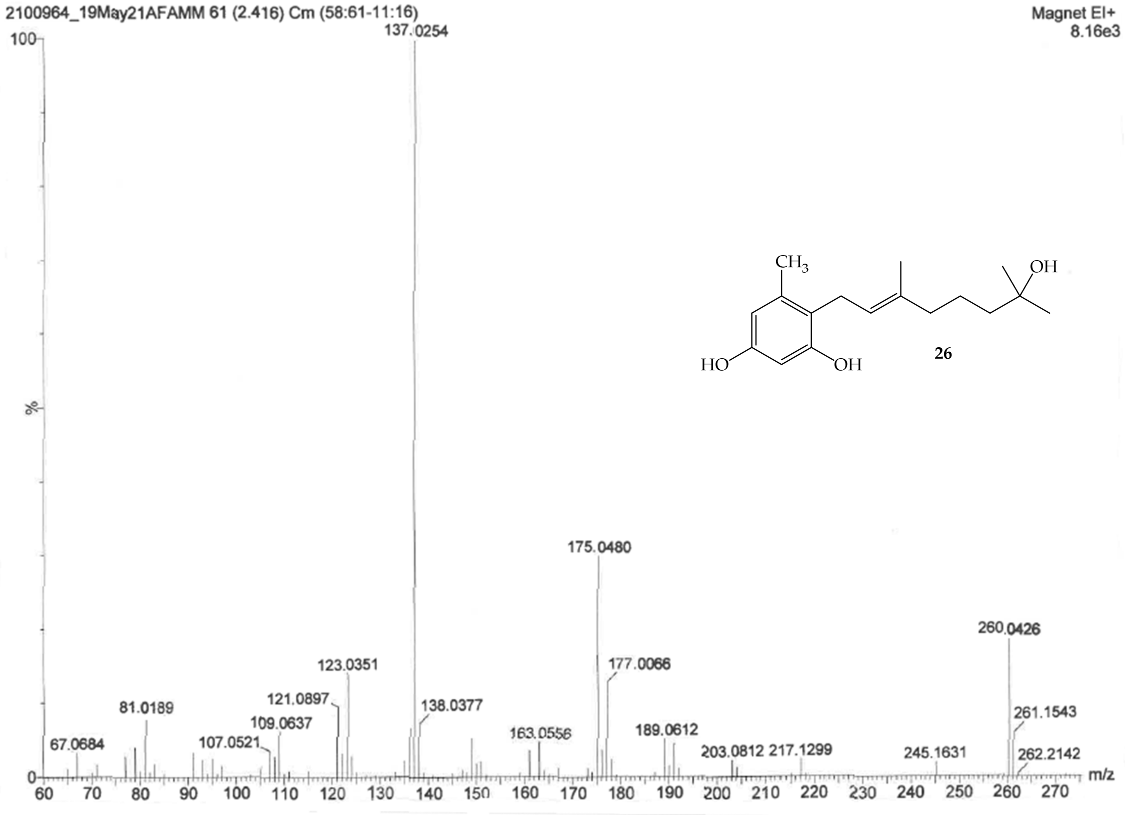

Figure S22: HRMS (EI+) spectrum of (E)-4-(7-hydroxy-3,7-dimethyloct-2-en-1-yl)-5-methylbenzene-1,3-diol (**26**)

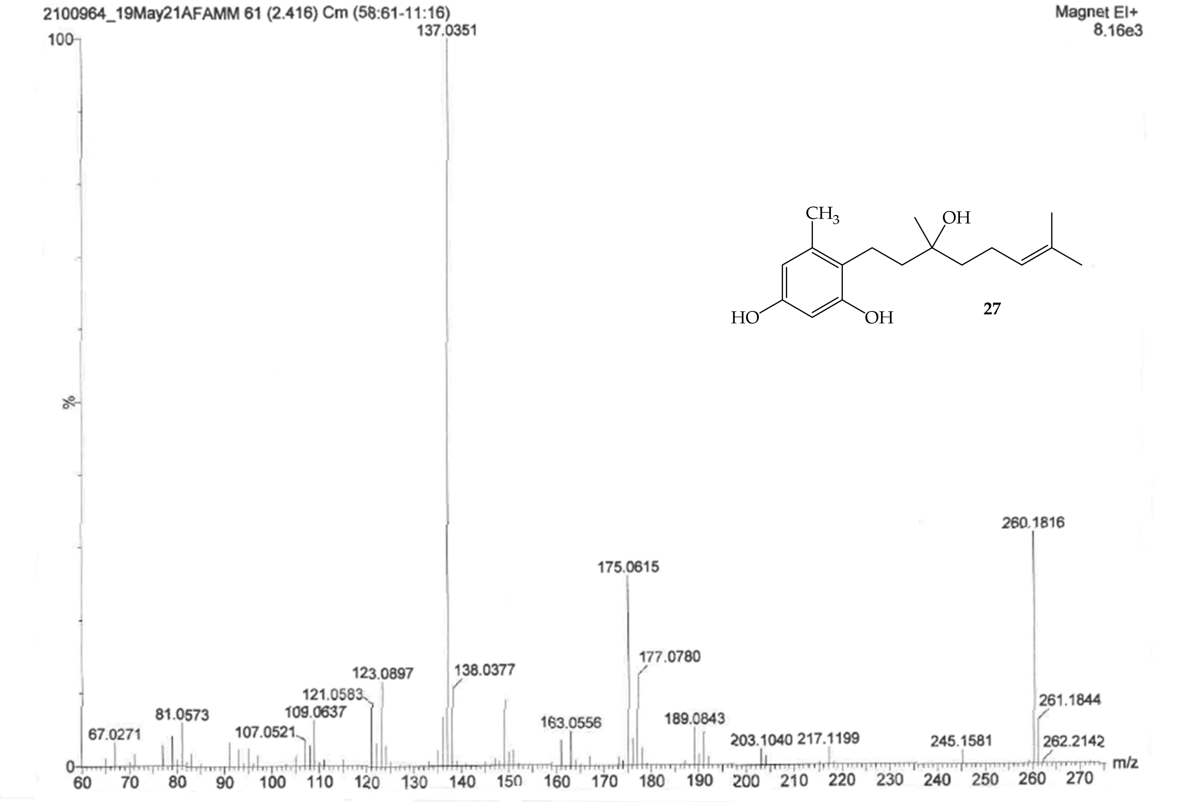

Figure S23: HRMS (EI+) spectrum of 4-(3-hydroxy-3,7-dimethyloct-6-en-1-yl)-5-methylbenzene-1,3-diol (**27**)

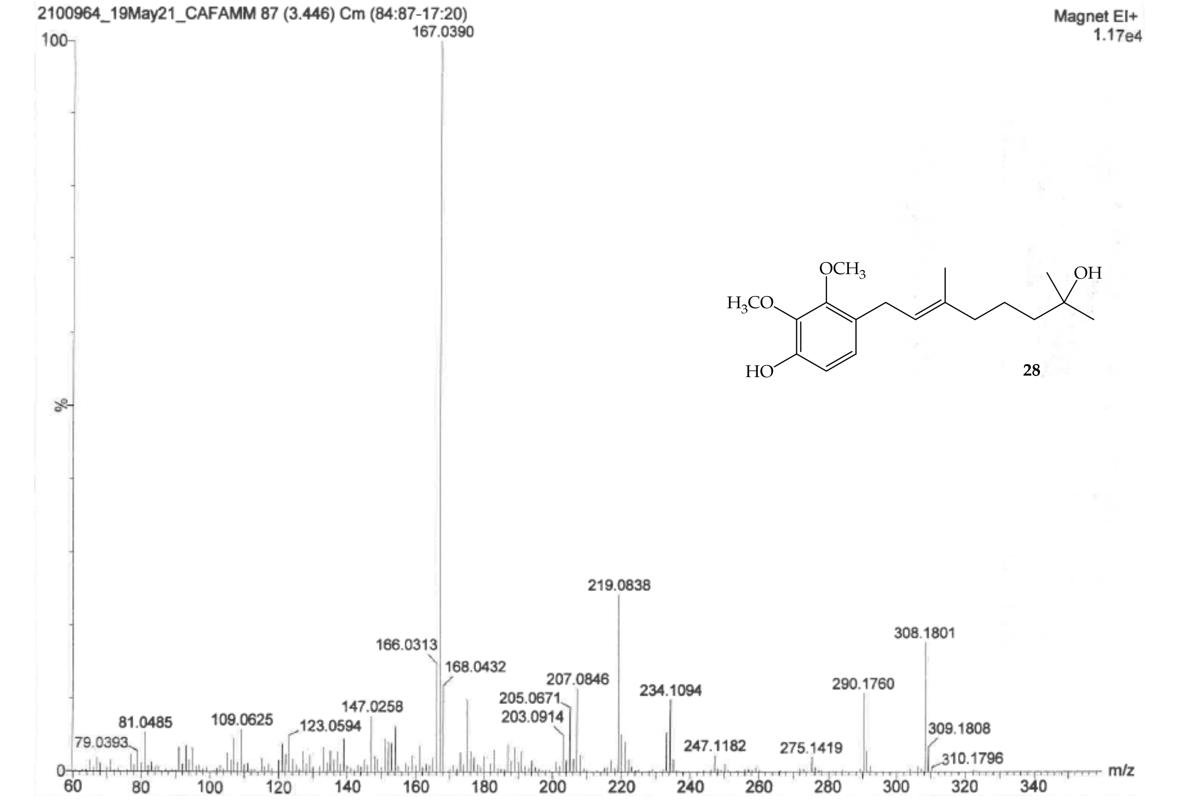

Figure S24: HRMS (EI+) spectrum of (E)-4-(7-hydroxy-3,7-dimethyloct-2-en-1-yl)-2,3-dimethoxyphenol (**28**)

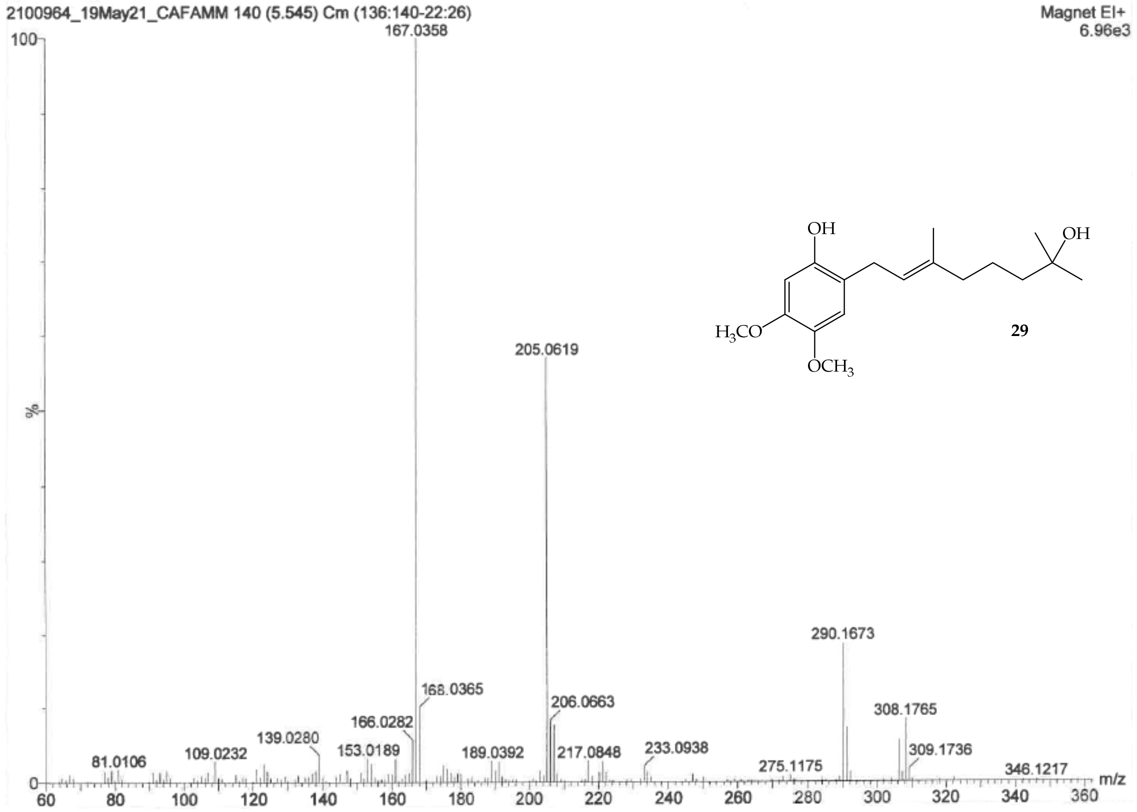

Figure S25: HRMS (EI$^+$) spectrum of (E)-2-(7-hydroxy-3,7-dimethyloct-2-en-1-yl)-4,5-dimethoxyphenol (**29**)

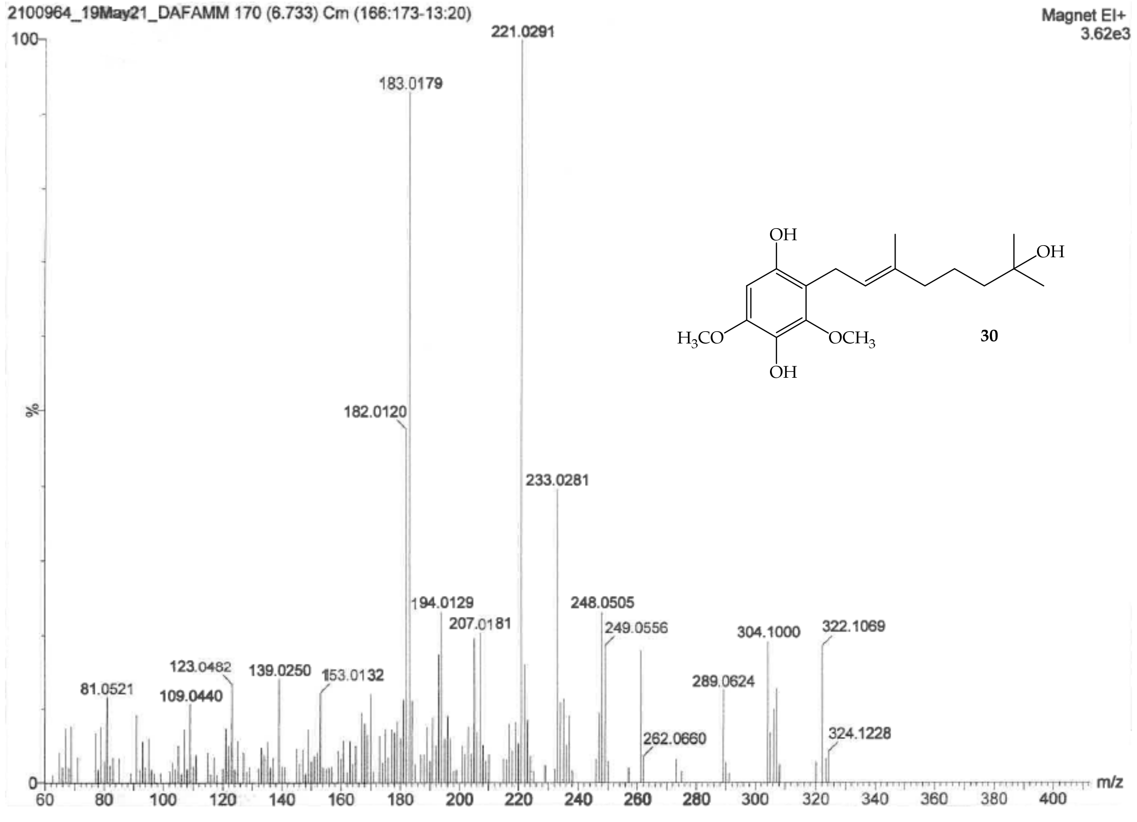

Figure S26: HRMS (EI$^+$) spectrum of (E)-2-(7-hydroxy-3,7-dimethyloct-2-en-1-yl)-3,5-dimethoxybenzene-1,4-diol (**30**)

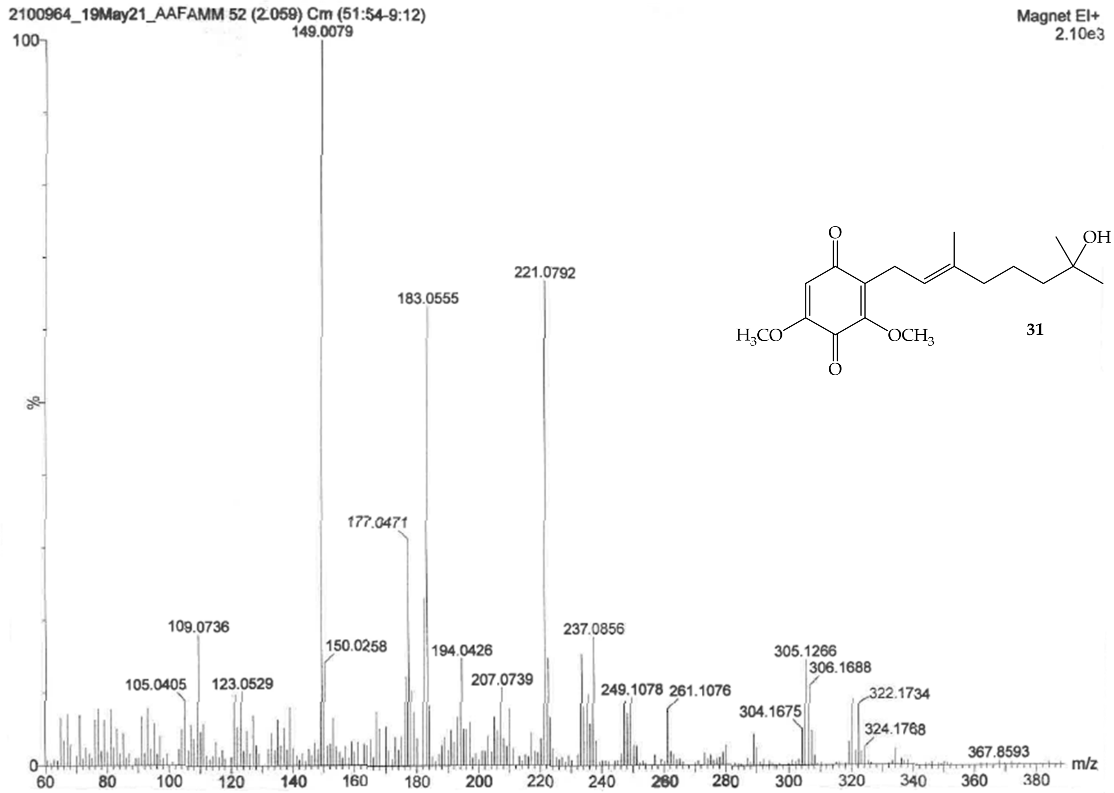

Figure S27: HRMS (EI+) spectrum of (E)-2-(7-hydroxy-3,7-dimethyloct-2-en-1-yl)-3,5-dimethoxycyclohexa-2,5-diene-1,4-dione (**31**)

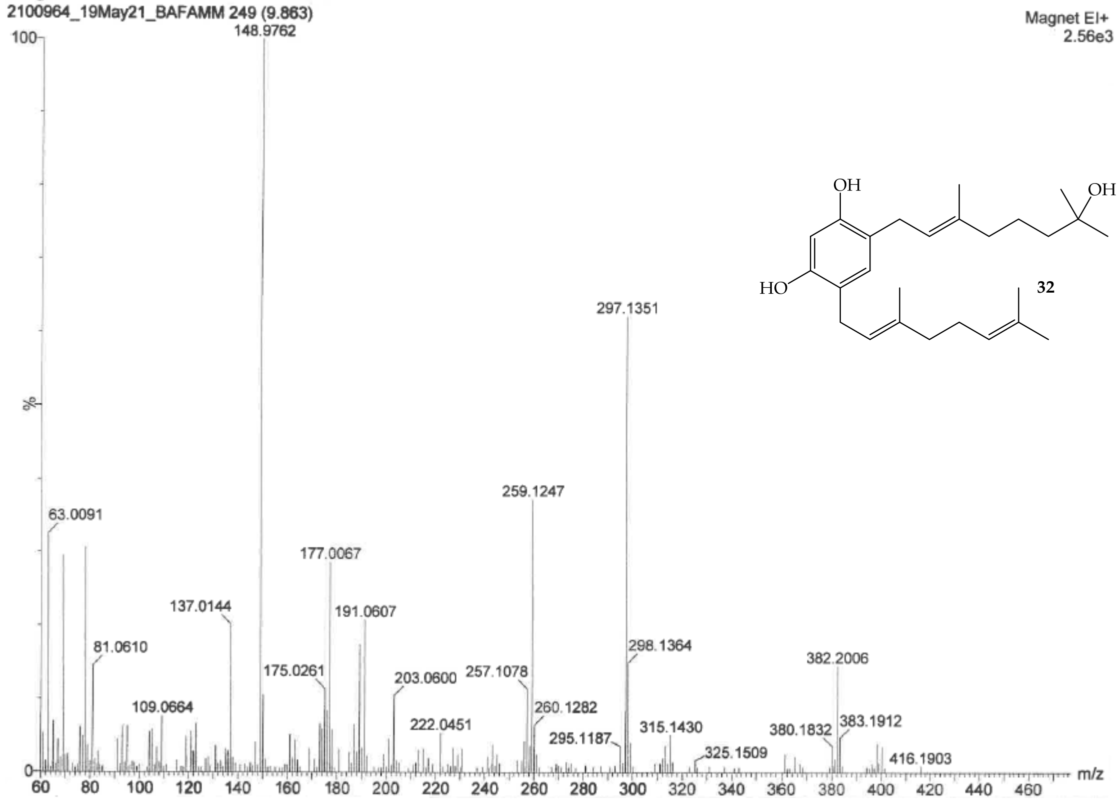

Figure S28: HRMS (EI+) spectrum of 4-((E)-3,7-dimethylocta-2,6-dien-1-yl)-6-((E)-7-hydroxy-3,7-dimethyloct-2-en-1-yl)benzene-1,3-diol (**32**)

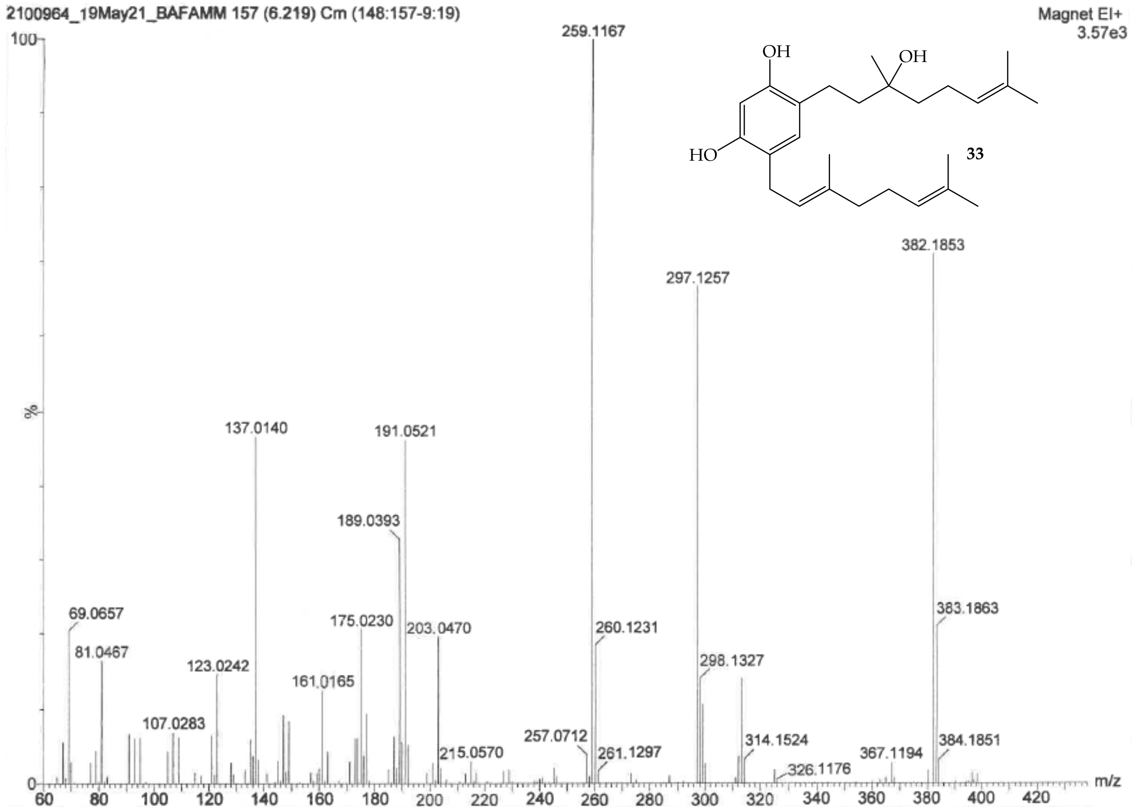

Figure S29: HRMS (EI$^+$) spectrum of (E)-4-(3,7-dimethylocta-2,6-dien-1-yl)-6-(3-hydroxy-3,7-dimethyloct-6-en-1-yl)benzene-1,3-diol (**33**)

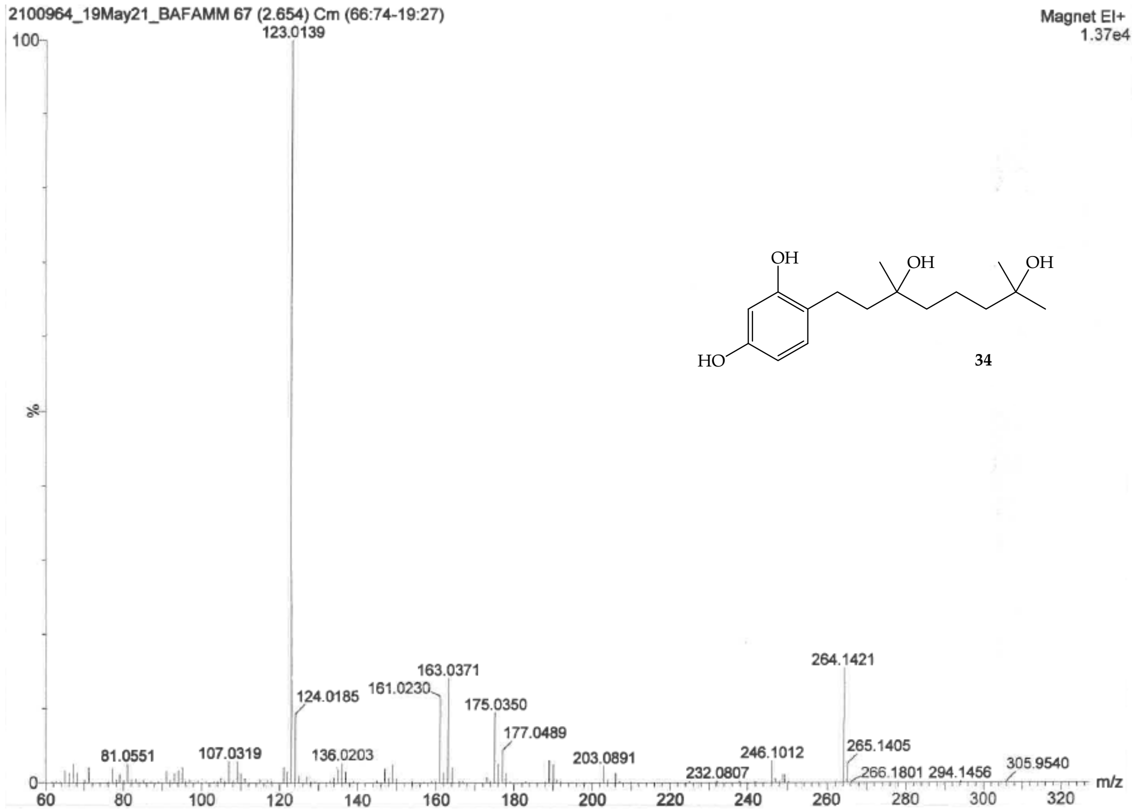

Figure S30: HRMS (EI$^+$) spectrum of 4-(3,7-dihydroxy-3,7-dimethyloctyl)benzene-1,3-diol (**34**)

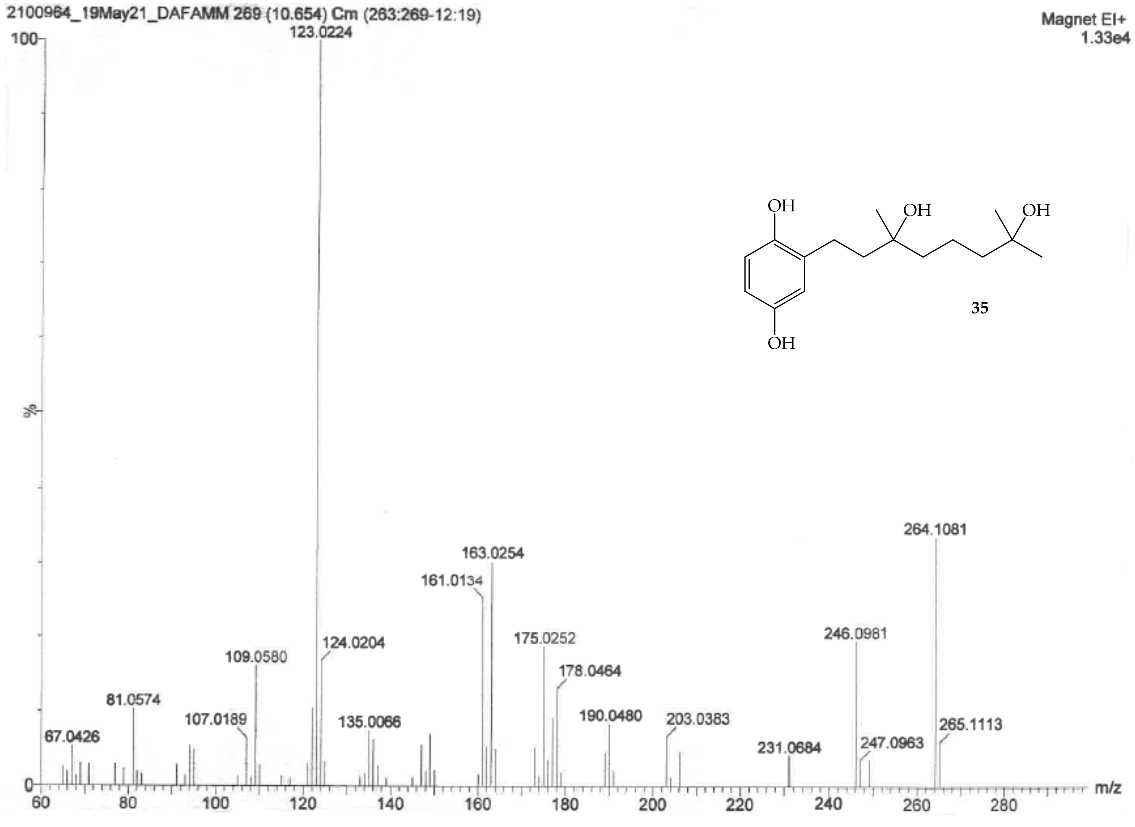

Figure S31: HRMS (EI+) spectrum of 2-(3,7-dihydroxy-3,7-dimethyloctyl)benzene-1,4-diol (**35**)

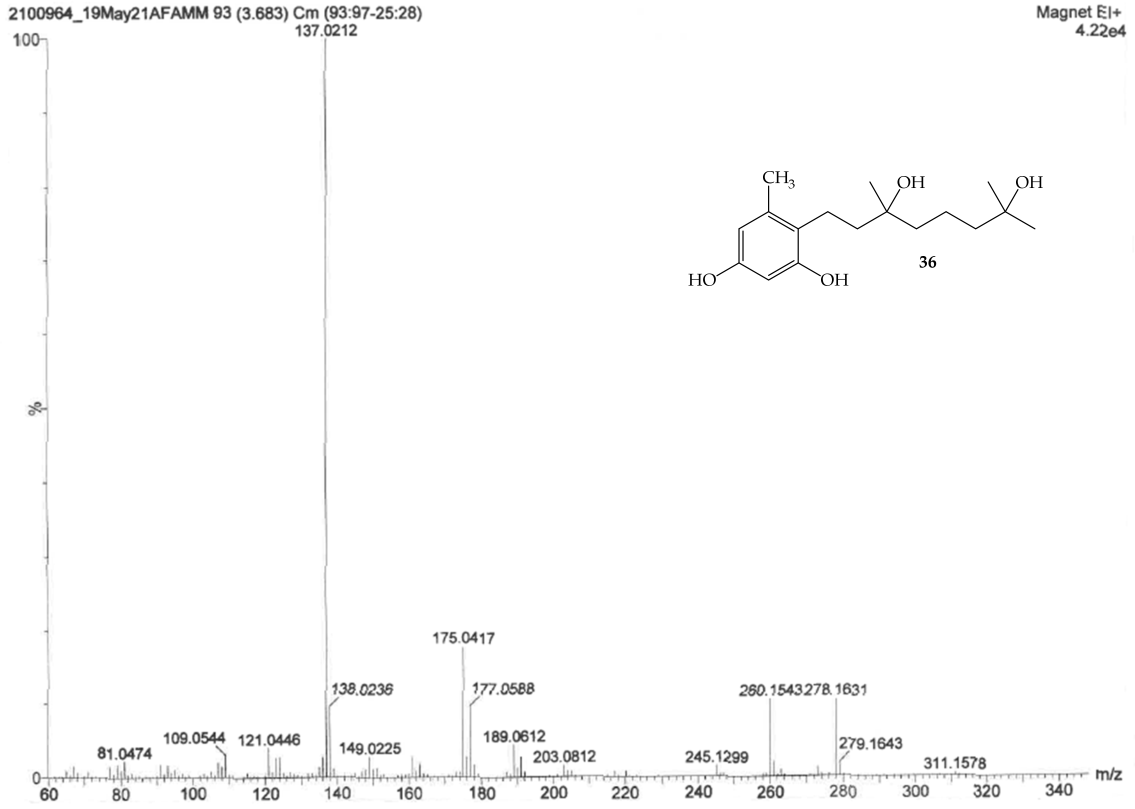

Figure S32: HRMS (EI+) spectrum of 4-(3,7-dihydroxy-3,7-dimethyloctyl)-5-methylbenzene-1,3-diol (**36**)

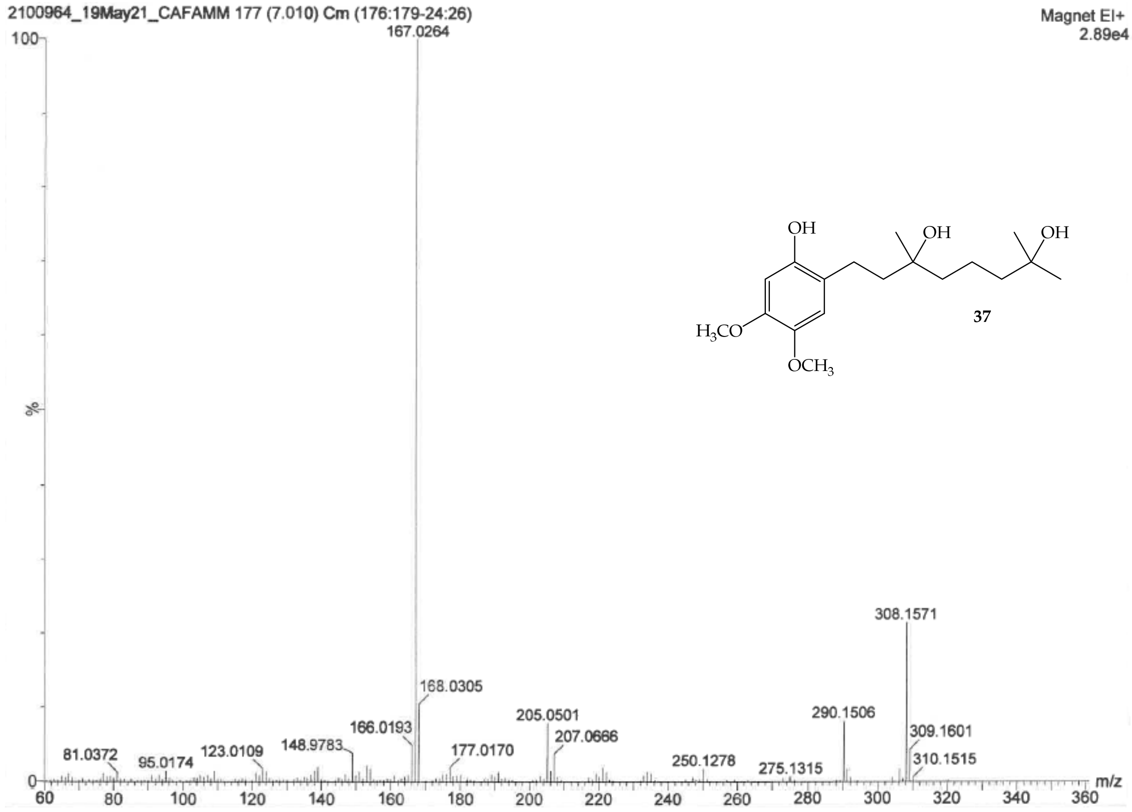

Figure S33: HRMS (EI$^+$) spectrum of 8-(2-hydroxy-4,5-dimethoxyphenyl)-2,6-dimethyloctane-2,6-diol (**37**)

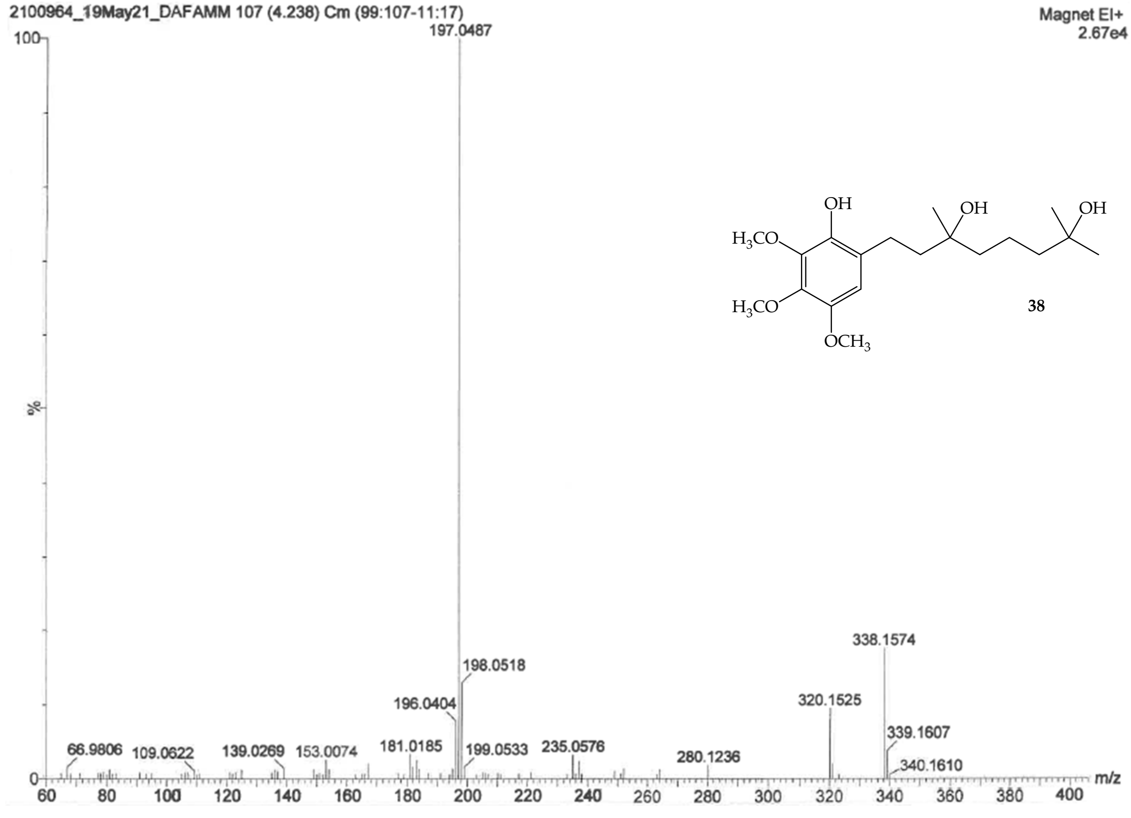

Figure S34: HRMS (EI$^+$) spectrum of 8-(2-hydroxy-3,4,5-trimethoxyphenyl)-2,6-dimethyloctane-2,6-diol (**38**)

© 2021 by the authors. Submitted for possible open access publication under the terms and conditions of the Creative Commons Attribution (CC BY) license (http://creativecommons.org/licenses/by/4.0/).
